# Inhibition of cytotoxic self-assembly of HEWL through promoting fibrillation by new synthesized α-hydroxycarbamoylphosphinic acids†

**DOI:** 10.1039/d4ra02969k

**Published:** 2024-10-01

**Authors:** Mohsen Mahdavimehr, Babak Kaboudin, Saied Alaie, Farimah Tondkar, Zahra Mahmoudi Eshkaftaki, Mohammad-Bagher Ebrahim-Habibi, Mojtaba Ghashghaee, Elham Tahmasebi, Tianjian Zhang, Yanlong Gu, Ali Akbar Meratan

**Affiliations:** a Department of Biological Sciences, Institute for Advanced Studies in Basic Sciences (IASBS) Zanjan 45137-66731 Iran a.meratan@iasbs.ac.ir a.meratan@gmail.com; b Department of Chemistry, Institute for Advanced Studies in Basic Sciences (IASBS) Zanjan 45137-66731 Iran kaboudin@gmail.com; c Microbiology and Biotechnology Group, Research Institute of Petroleum Industry Tehran Iran; d School of Chemistry and Chemical Engineering, Huazhong University of Science & Technology Wuhan 430074 China

## Abstract

The main objective of the present study is to investigate the potency of new synthesized hydroxycarbamoyl phosphinic acid derivatives in modulating cytotoxic fibrillogenesis of hen egg white lysozyme (HEWL), as a common model in protein aggregation studies. Hydroxycarbamoyl phosphinic acid derivatives were prepared by the reaction of α-hydroxyalkylphosphinic acids with isocyanates (or isothiocyanates) in the presence of trimethylsilyl chloride (TMSCl). The designed process involves the condensation reaction leading to formation of new C sp^2^–P bond formation. The synthesis and purity of novel designed compounds were confirmed by NMR, LC-MS, and HPLC techniques. A range of experiments, including thioflavin T (ThT) and 8-anilino-1-naphthalenesulfonic acid (ANS) fluorescence assays, Congo red binding measurement, atomic force microscopy imaging, MTT-based cell viability and hemolysis assays were employed to investigate anti-amyloidogenic effects of tested compounds. The obtained results demonstrate that these compounds are able to significantly modulate the self-assembly process of HEWL *via* shortening of nucleation phase leading to the acceleration of fibrillation and appearance of very large and thick fibrils with decreased surface hydrophobicity and cytotoxicity. Based on ANS binding data, we suggest that increased exposure of hydrophobic patches of oligomeric species is the possible mechanism by which tested compounds promote self-assembly process of HEWL. Fluorescence anisotropy and molecular docking studies indicate the interaction of both synthesized compounds with HEWL, and more specifically with residues that are situated in the highly aggregation-prone β-domain region of protein. This study unveils the potential of hydroxyalkylphosphinic acids as modulators of amyloid fibrillation highlighting these compounds as a promising approach for targeting protein aggregates associated with neurodegenerative diseases.

## Abstract 2

New synthesized α-hydroxycarbamoylphosphinic acids are potent modulator of HEWL amyloid fibrillation. Shortening of the nucleation phase of HEWL leading to the acceleration of fibrillation is the proposed mechanism of action of these compounds.

## Graphical abstract

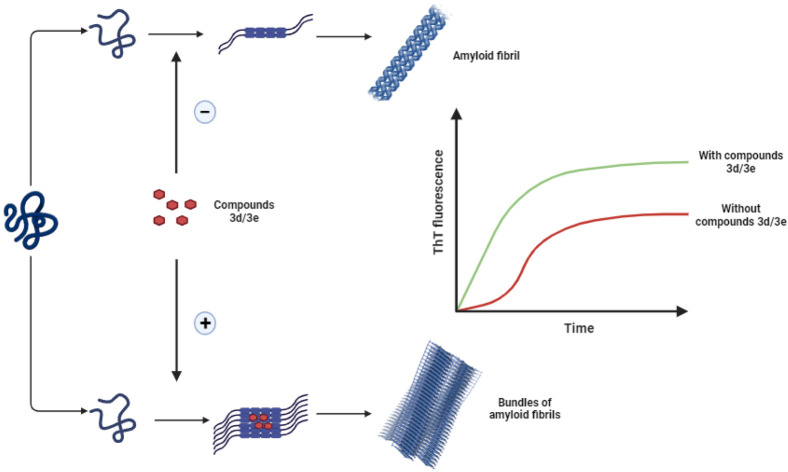

## Introduction

Neurodegenerative diseases refer to an array of human disorders resulting from misfolding and aberrant assembly of different peptides/proteins into fibrillar structures known as amyloid fibrils. Protein aggregation leading to fibril formation has been suggested to be associated with various neurodegenerative diseases such as Alzheimer's disease (AD), Parkinson's disease (PD), Huntington's disease (HD), and prion diseases that are caused by the aggregation of amyloid-beta (Aβ), α-synuclein, polyglutamine, and prion proteins, respectively.^1–5^ Amyloid fibrillation is a complicate process that involves the misfolding of monomeric species followed by the formation of oligomers and protofibrils, and finally growth of these transient heterogenous species into matures amyloid fibrils.^6^ Significant evidence indicates that transient soluble oligomers and protofibrils, emerging in the initial stages of amyloid fibrillation, are main species causing neurotoxicity,^7^ mostly by disrupting membrane integrity, affecting mitochondrial functionality and causing oxidative stress, eventually resulting in cell damage and death. For example, the toxic effects of Aβ in AD and α-synuclein in PD are primarily linked to their oligomeric forms.^8,9^

Regarding the hen egg white lysozyme (HEWL), recent studies have revealed that the oligomeric intermediates, but not monomers nor protofilaments, formed in the course of amyloid fibrillation can cause cell death in a variety of cell lines, including primary neuronal cells and fibroblasts, as well as the neuroblastoma IMR-32 cell line.^10–13^ In contrast, mature amyloid fibrils are comparatively less harmful or even non-toxic to the neuronal cells.^14^ Considering soluble oligomers as the most toxic species, reducing the prevalence of these species through blocking amyloid fibrillation process at early stages or accelerating assembly process *via* shortening of nucleation phase might decrease their neurotoxicity.^15–17^ Several studies indicate the potency of small molecules in manipulating the fibrillogenesis of various peptides and proteins through different mechanisms such as accelerating fibrillogenesis, stabilizing oligomers and protofibrils, and blocking the formation of amyloid fibrils. Thus, these compounds can be suitable tools for modulating amyloid fibril formation *via* interfering with various stages of amyloid fibrillation. Among them, organic compounds have attracted considerable attention and have reported to influence amyloid fibrillation of many proteins *via* different mechanisms.^18–23^

Phosphinic peptides and pseudopeptides are interesting compounds that show a variety of important and applicable properties in biological systems.^24^ α-Functionalized phosphinic acids such as α-hydroxyphosphinic and α-aminophosphinic acids are interesting class of compounds possessing broad biological activities.^25–27^ These compounds are useful intermediates for the synthesis of α-hydroxyphosphinyl peptides with inhibitory activity against rennin.^28^ Furthermore, the conjunction of two functional groups on the phosphinic acids affords powerful inhibitors of HIV-1 protease and human aminopeptidase N.^29^ In contrast to many reports regarding effects of phosphinic acids derivatives, relatively few studies are performed on the biological properties of α-hydroxyphosphinates.^30^ The results obtained by several studies show phosphonates derivatives as potential acetylcholinesterase and Aβ aggregation inhibitors, suggesting these compounds as anti-Alzheimer agents.^31–33^ However, most of these studies are relating to cytotoxicity, antioxidant, and the potential of these compounds to inhibit acetylcholinesterase activity, as a marker of AD.^33–38^ For instance, the results reported by El-Sayed *et al.*^33^ regarding the effect of some new phosphazine and phosphazide derivatives on metal-induced aggregation of Aβ, evaluated by thioflavin T (ThT) fluorescence assay, is the only study relating to the anti-aggregation effects of these compounds, without providing any possible mechanism by which these components may modulate the amyloid fibrillation of Aβ. As part of our ongoing research to develop the synthesis and biological applications of novel organophosphonate esters,^31,34–36^ in the present study, we have synthesized some novel hydroxycarbamoyl phosphinic acids by the reaction of 1-hydroxyalkylphosphinic acids with isocyanates in the presence of TMSCl/Et_3_N. The success synthesis and purity of compounds were confirmed by Nuclear Magnetic Resonance (NMR), Liquid Chromatography-Mass Spectrometry (LC-MS), and high-performance liquid chromatography (HPLC). This was followed by investigating the anti-aggregation activity and the possible mechanism by which these new synthesized hydroxycarbamoyl phosphinic acid derivatives modulate the assembly process of HEWL *in vitro*. Based on ThT, 1-anilino-naphthalene 8-sulfonate (ANS), and atomic force microscopy (AFM) images we propose that promoting the formation of oligomers with solvent-exposed hydrophobic regions is the mechanism by which these compounds accelerate the HEWL assembly process and lead to the formation of aggregated species with decreased surface hydrophobicity and mitigated cytotoxicity and hemolytic activity. Taken together, our results indicate the capacity of these compounds to act as effective modulators of protein fibrillation highlighting their potential as possible candidates in searching for new anti-aggregation drugs.

## Materials and methods

### Materials

NMR spectra were obtained with a 400 MHz Bruker Avance instrument with the chemical shifts being reported as *δ* ppm and couplings expressed in Hertz. The chemical shift data for each signal on ^1^H NMR are given in units of *δ* relative to DMSO-d_6_. For ^13^C NMR spectra, the chemical shifts in DMSO are recorded relative to the DMSO-d_6_ resonance (*δ* = 40.45 ppm). Silica gel column chromatography was carried out with Silica gel 100 (Merck No. 10184). Merck Silica-gel 60 F_254_ plates (No. 5744) were used for the preparative TLC. Melting points are uncorrected. HEWL, ThT, Congo red (CR), ANS, Nile red (NR), and 3-(4,5-dimethyl tiazol-2yl)-2,5 diphenyl tetrazolium bromide (MTT) were purchased from Sigma (St. Louis, MO, USA). SH-SY5Y cells were a gift from Dr Karima (Shahid Beheshti University of Medical Sciences, Tehran, Iran). The cell culture medium (DMEM-F12), fetal bovine serum (FBS), and penicillin–streptomycin antibiotics were purchased from Gibco BRL (Life Technology, Paisley, Scotland). All other chemicals were obtained from Merck (Darmstadt, Germany) and were reagent grade.

### Methods

#### *General procedure for* the synthesis of 1-hydroxycarbamoyl **phosph**inic acids 3

A mixture of trimethylsilyl chloride (TMSCl, 6 mmol, 0.75 mL) and triethyl amine (Et_3_N, 6 mmol, 0.83 mL) was added dropwise to a suspension of 1-hydroxyalkylphosphinic acid (1 mmol) [1-hydroxyalkylphosphinic acids 1 were obtained in gram scale from the reaction of aldehyde and hypophosphorus acid at reflux ethanol for 48 h, according to ref. 41 and 42] in toluene (5–8 mL) under argon and the mixture was stirred at 0 °C for 3 h. Isocyanate (2.0 mmol) was added dropwise to the reaction mixture and the mixture was stirred at room temperature for 18–22 h. MeOH (10 mL) was added to this mixture and the mixture was stirred at reflux for 1 h. After evaporation of the solvent, NaHCO_3_ (5%, 20 mL) was added to the solid residue and the mixture was washed with EtOAc (3 × 10 mL). The aqueous phase neutralized by HCl (5%) and the crude product was extracted with EtOAc (3 × 10 mL). The organic phase dried over anhydrous Na_2_SO_4_ and the solvent was evaporated. The compound 3 was obtained as a colorless solid that was further purified by flash chromatography (CHCl_3_/MeOH = 10 : 1) and pure compound was obtained 40–67% yield. All products gave satisfactory spectral data in accordance with the assigned structures.

### HPLC analysis

Separation and determination of the compounds were carried out on a Knauer HPLC instrument consisting of a pump model Azura P6.1L and a Knauer UV-Vis detector model UVD 2.1L using a Nucleosil-100C18 analytical column (125 mm × 4.0 mm, 5 μm particle size) from Knauer (GmbH, Germany) at 25 °C. Samples were analyzed using a gradient elution with acetonitrile (A) and 1% (v/v) aqueous acetic acid solution (B) as follows: 1% A (0–4 min), 1–15% A (4–10 min), and 15% A (15–25 min) at a flow rate of 0.9 mL min^−1^. The detections were carried out at 280 nm.

#### 1-Hydroxy(phenyl)methyl(phenylcarbamoyl)phosphinic acid (3a)

White crystalline solid: mp 202–203 °C; ^1^H NMR (DMSO-d_6_, 400 MHz): 5.19 (1H, d, *J* = 7.6 Hz), 4.1–5.1(1H, br, OH), 7.15 (1H, m), 7.35 (5H, m), 7.46 (2H, m), 7.79 (2H, m), 10.33 (1H, –NH); ^31^P NMR (DMSO-d_6_-162.0 MHz): 22.43 ppm; ^13^C NMR (DMSO-d_6_-100.6 MHz): 69.4 (*J*_PC_ = 113.7 Hz), 120.92 (d, *J*_PC_ = 10.1 Hz), 124.9, 129.14, 138.19 (d, *J*_PC_ = 10.6 Hz), 170.0 (d, *J*_PC_ = 147.8 Hz). FT IR (KBr) *ν* (cm^−1^): 3435, 3324, 1630, 1116 (P=O), 984; HRMS(ESI): calcd for C_14_H_13_NO_4_P [M − H]: 290.0582, found: 290.0588.

#### 1-Hydroxy(phenyl)methyl(phenylcarbamothioyl)phosphinic acid (3b)

White crystalline solid: mp 158–159 °C; ^1^H NMR (DMSO-d_6_, 400 MHz): 5.45 (1H, *J* = 7.6), 4.1–5.1 (1H, br, OH), 7.28–7.34 (4H, m), 7.41–7.48 (4H, m), 7.85 (2H, d, *J* = 8.0 Hz), 11.65 (–NH, s); ^31^P NMR (DMSO-d_6_-162.0 MHz): 22.26 ppm; ^13^C NMR (DMSO-d_6_-100.6 MHz): 69.5 (d, *J*_PC_ = 123.7 Hz), 124.0 (d, *J*_PC_ = 14.1 Hz), 127.33, 129.02, 138.42, 139.1 (d, *J*_PC_ = 11.1 Hz), 199.7 (d, *J*_PC_ = 106.6 Hz); FT IR (KBr) *ν* (cm^−1^): 3793, 3426, 3268, 1596, 1367, 984 (P=O); HRMS(ESI): calcd for C_14_H_13_NO_3_PS [M − H]: 306.0354, found: 306.0359.

#### 1-Hydroxy(2-chlorophenyl)methyl(phenylcarbamoyl)phosphinic acid (3c)

White crystalline solid: mp 175–176 °C; ^1^H NMR (DMSO-d_6_, 400 MHz): 5.39 (1H, d, *J* = 7.2 Hz), 6.15–6.84 (1H, br, OH), 7.16 (1H, d, *J* = 6.4 Hz), 7.34–7.56 (5H, m), 7.74–7.81 (3H, m), 10.40 (1H, –NH); ^31^P NMR (DMSO-d_6_-162.0 MHz): 20.26 ppm; ^13^C NMR (DMSO-d_6_-100.6 MHz): 65.7 (d, *J*_PC_ = 118.7 Hz), 120.7, 134.7 (d, *J*_PC_ = 7.0 Hz), 135.5, 137.4 (d, *J*_PC_ = 5.0 Hz), 169.8 (d, *J*_PC_ = 148.8 Hz); FT IR (KBr) *ν* (cm^−1^): 3770, 3368, 1631, 1029 (P=O), 805; HRMS(ESI): calcd for C_14_H_12_ClNO_4_P [M − H]: 324.0192, found: 324.0198.

#### 1-Hydroxy(4-chlorophenyl)methyl(cyclohexylcarbamoyl)phosphinic acid (3d)

White crystalline solid: mp 181–183 °C; ^1^H NMR (DMSO-d_6_, 400 MHz): 1.24–1.28 (2H, m), 1.30–1.37 (4H, m), 1.50–1.70 (4H, m), 3.60–3.75 (1H, br), 5.09 (1H, d, *J* = 8.0 Hz), 5.83–6.63 (1H, br), 7.43 (4H, s), 8.19 (1H, d, *J* = 8.0 Hz); ^31^P NMR (DMSO-d_6_-162.0 MHz): 19.52 ppm; ^13^C NMR (DMSO-d_6_-100.6 MHz): 25.5 (d, *J*_PC_ = 26.2 Hz), 32.3 (d, *J*_PC_ = 3.0 Hz), 48.1 (d, *J*_PC_ = 4.0 Hz), 69.1 (d, *J*_PC_ = 112.7 Hz), 128.1 (d, *J*_PC_ = 2.0 Hz), 129.7 (d, *J*_PC_ = 5.0 Hz), 132.3, 137.5, 169.8 (d, *J*_PC_ = 146 Hz); FT IR (KBr) *ν* (cm^−1^): 3416, 2932, 2850, 1619, 1035 (P=O), 823. HRMS(ESI): calcd for C_14_H_18_ClNO_4_P [M − H]: 330.0662, found: 330.0667.

#### 1-Hydroxy(4-chlorophenyl)methyl(phenylcarbamothioyl)phosphinic acid (3e)

White crystalline solid: mp 153–154 °C; ^1^H NMR (DMSO-d_6_, 400 MHz): 5.04 (1H, d, *J* = 10.8 Hz), 5.95–6.84 (1H, br, OH), 7.22–7.30 (3H, m), 7.39–7.44 (4H, m), 8.00 (2H, d, *J* = 7.6 Hz), 10.93–11.98 (–NH, br); ^31^P NMR (DMSO-d_6_-162.0 MHz): 16.92 ppm; ^13^C NMR (DMSO-d_6_-100.6 MHz): 70.6 (d, *J*_PC_ = 116.7 Hz), 122.27, 131.39, 139.0 (d, *J*_PC_ = 10.0 Hz), 139.4, 206.8 (d, *J*_PC_ = 98.6 Hz); FT IR (KBr) *ν* (cm^−1^): 3424, 3266, 1376, 1076 (P=O), 807; HRMS(ESI): calcd for C_14_H_12_ClNO_3_PS [M − H]: 339.9964, found: 339.9969.

#### 1-Hydroxy(4-chlorophenyl)methyl(benzylcarbamoyl)phosphinic acid (3f)

White crystalline solid: mp 195–196 °C; ^1^H NMR (DMSO-d_6_, 400 MHz): 4.28–4.32 (2H, m), 5.10 (1H, d, *J* = 8.0 Hz), 7.21–7.26 (3H, m), 7.29–7.33 (2H, m), 7.38–7.42 (4H, m), 9.05 (2H, t, *J* = 6.0 Hz); ^31^P NMR (DMSO-d_6_-162.0 MHz): 20.18 ppm; ^13^C NMR (DMSO-d_6_-100.6 MHz): 45.7, 68.9 (d, *J*_PC_ = 11.6 Hz), 128.6, 132.4, 137.3, 139.1, 170.2 (d, *J*_PC_ = 149.9 Hz); FT IR (KBr) *ν* (cm^−1^): 3254, 3058, 1642, 1058 (P=O), 807; HRMS(ESI): calcd for C_15_H_14_ClNO_4_P [M − H]: 338.0349, found: 338.0354.

#### 1-Hydroxy(4-methoxyphenyl)methyl(phenylcarbamothioyl)phosphinic acid (3g)

Yellow crystalline solid: mp 145–146 °C; ^1^H NMR (DMSO-d_6_, 400 MHz): 3.76 (3H, s), 5.04 (1H, d, *J* = 8.0 Hz), 5.74–6.64 (1H, OH), 6.93 (2H, d, *J* = 8.4 Hz), 7.30 (1H, t, *J* = 4.0 Hz), 7.38–7.45 (4H, m), 7.84 (2H, d, *J* = 8.0 Hz), 11.63 (1H, d, *J* = 8.0 Hz); ^31^P NMR (DMSO-d_6_-162.0 MHz): 22.37 ppm; ^13^C NMR (DMSO-d_6_-100.6 MHz): 55.5 (3H, s), 69.2 (d, *J*_PC_ = 123.5 Hz), 113.78, 123.35, 127.65, 138.5 (d, *J* = 11.0 Hz), 159.2 (d, *J*_PC_ = 2.0 Hz), 199.7 (d, *J*_PC_ = 104.6 Hz); FT IR (KBr) *ν* (cm^−1^): 3444, 3268, 1509, 1030 (P=O), 804; HRMS(ESI): calcd for C_15_H_15_NO_4_PS [M − H]: 336.0459, found: 336.0465.

#### 1-Hydroxy(4-methoxyphenyl)methyl(cyclohexylcarbamoyl)phosphinic acid (3h)

White crystalline solid: mp 154–155 °C; ^1^H NMR (DMSO-d_6_, 400 MHz): 1.24–1.28 (1H, m), 1.30–1.38 (4H, m), 1.55–1.67 (5H, m), 3.54–3.69 (1H, br), 3.75 (3H, s), 4.98 (1H, d, *J* = 7.2 Hz), 5.83–6.63 (1H, br), 6.90 (2H, d, *J* = 8.4 Hz), 7.31 (2H, d, *J* = 7.2 Hz), 8.13 (1H, d, *J* = 8.4 Hz); ^31^P NMR (DMSO-d_6_-162.0 MHz): 20.44 ppm; ^13^C NMR (DMSO-d_6_-100.6 MHz): 25.3 (d, *J*_PC_ = 26.2 Hz), 32.3 (d, *J*_PC_ = 3.0 Hz), 48.0 (d, *J*_PC_ = 4.0 Hz), 55.5, 69.1 (d, *J*_PC_ = 112.7 Hz), 113.7, 129.3 (d, *J*_PC_ = 5.0 Hz), 129.9, 159.2 (d, *J*_PC_ = 3.0 Hz), 169.4 (d, *J*_PC_ = 145.9 Hz); FT IR (KBr) *ν* (cm^−1^): 3416, 2932, 2850, 1619, 1035 (P=O), 823; HRMS(ESI): calcd for C_15_H_21_NO_5_P [M − H]: 326.1157, found: 326.1163.

#### 1-Hydroxy(4-methoxyphenyl)methyl(2-phenylethylcarbamothioyl)phosphinic acid (3i)

Yellow crystalline solid: mp 133–134 °C; ^1^H NMR (DMSO-d_6_, 400 MHz): 2.74–2.96 (2H, m), 3.74 (3H, s), 3.78–3.92 (2H, m), 5.33 (1H, *J* = 7.6 Hz), 5.74–6.36 (OH, br), 6.90 (2H, d, *J* = 8.4 Hz), 7.23 (2H, t, *J* = 7.6 Hz), 7.29–7.36 (5H, m), 10.42 (–NH, d, *J* = 7.2 Hz); ^31^P NMR (DMSO-d_6_-162.0 MHz): 22.00 ppm; ^13^C NMR (DMSO-d_6_-100.6 MHz): 33.04, 46.4 (d, *J*_PC_ = 5.0 Hz), 55.54, 69.0 (d, *J*_PC_ = 124.7 Hz), 113.65, 126.78, 128.9 (d, *J*_PC_ = 5.0 Hz), 129.6 (d, *J*_PC_ = 5.0 Hz), 130.2, 139.2, 159.22, 199.6 (d, *J*_PC_ = 105.6 Hz); FT IR (KBr) *ν* (cm^−1^): 3424, 3286, 2940, 1512, 1253 (P=O), 954; HRMS(ESI): calcd for C_17_H_19_NO_5_P [M − H]: 348.1001, found: 348.1006.

#### 1-Hydroxyheptyl(phenylcarbamoyl)phosphinic acid (3j)

White crystalline solid: mp 137–138 °C; ^1^H NMR (DMSO-d_6_, 400 MHz): 1.18–1.52 (9H, m), 1.55–1.63 (4H, m), 3.94 (1H, d, *J* = 8.8 Hz), 4.54–6.43 (OH, br), 7.13 (1H, tr, *J* = 6.8 Hz), 7.34 (2H, tr, *J* = 7.6 Hz), 7.77 (2H, d, *J* = 8.0 Hz), 10.25 (NH, s); ^31^P NMR (DMSO-d_6_-162.0 MHz): 23.90 ppm; ^13^C NMR (DMSO-d_6_-100.6 MHz): 22.4, 25.4 (tr, *J*_PC_ = 17.1 Hz), 28.7 (d, *J*_PC_ = 10.1 Hz), 29.4, 29.9, 31.5 (d, *J*_PC_ = 7.0 Hz), 66.9 (d, *J*_PC_ = 115.7 Hz), 120.7, 125.38, 129.31, 137.6 (d, *J*_PC_ = 10.0 Hz), 169.8 (d, *J*_PC_ = 142.8 Hz); FT IR (KBr) *ν* (cm^−1^): 3328, 3261, 2925, 1684, 1071 (P=O), 748; HRMS(ESI): calcd for C_14_H_21_NO_4_P [M − H]: 298.1208, found: 298.1214.

#### 1-Hydroxyheptyl(phenylcarbamothioyl)phosphinic acid (3k)

White crystalline solid: mp 124–125 °C; ^1^H NMR (DMSO-d_6_, 400 MHz): 1.26–1.48 (10H, br), 1.54–1.71 (3H, br), 3.34–4.24 (1H, br), 7.21–7.32 (1H, br), 7.35–7.50 (2H, br), 7.80–7.92 (2H, br), 11.61–11.74 (1H, br); ^31^P NMR (DMSO-d_6_-162.0 MHz): 25.17 ppm; ^13^C NMR (DMSO-d_6_-100.6 MHz): 25.5, 28.7, 29.4, 66.6 (d, ^1^*J*_PC_ = 123.7 Hz), 123.4, 127.6, 129.2, 199.0 (d, *J*_PC_ = 103.6 Hz); FT IR (KBr) *ν* (cm^−1^): 3390, 3282, 2924, 1596, 1033 (P=O), 976; HRMS(ESI): calcd for C_14_H_21_NO_3_PS [M − H]: 314.0980, found: 314.0985.

### Sample preparation and HEWL fibrillation induction

The concentration of HEWL was determined by measuring the absorbance of protein at 280 nm using the extinction coefficient (*ε*^1mg/ml^) of 2.63.^39^ Compounds 3d and 3e were dissolved in dimethylsulfoxide (DMSO) to create concentrated stock solutions and stored at −20 °C for subsequent experiments. The incubating solutions had a final DMSO concentration from 0.02% to 0.2% (v/v). HEWL amyloid fibrils were produced based on our previous report with some modifications.^40^ Aliquots of HEWL, prepared at a final concentration of 100 μM in 50 mM glycine buffer (pH 2.2), were incubated alone or with increasing concentrations of compounds 3d and 3e at 57 °C for a period of 6 days, while being stirred at 500 rpm. The molar ratios of tested compounds to protein used in this study were 0 : 1, 0.1 : 1, 0.5 : 1 and 1 : 1. The concentration of oligomers/protofibrils is given in terms of the monomer's concentration throughout the article. Moreover, the concentration of amyloid fibrils was determined by subjecting the incubated samples to centrifugation at 21 000 g for 1 h, and then calculating the protein concentration in the resulting supernatant and subtracting it from the initial protein concentration.^41^

### Amyloid fibril detection and characterization

#### ThT fluorescence assay

To determine HEWL fibrillogenesis and evaluate possible modulating effects of tested compounds, incubated samples were added to ThT solution (made from a 5 mM ThT stock solution in 25 mM sodium phosphate, pH 6.5, filtered through a 0.22 μm filter paper). The final concentration of protein and ThT was 2 μM and 10 μM, respectively. After thorough mixing, the mixtures were incubated for 5 min and the fluorescence emission spectra of samples were recorded using excitation at 440 nm and emission at 485 nm. The excitation and emission slit widths were set at 5 nm and 10 nm, respectively.

#### ANS fluorescence assay

For ANS fluorescence measurements, aliquots of protein solutions were removed at various time intervals and diluted to a final concentration of 2 μM using deionized water containing 20 μM ANS. The samples were then excited at 350 nm and the resulting emission spectra were recorded from 360 to 600 nm.

#### Fluorescence anisotropy measurement

We employed Nile red (NR) fluorescence anisotropy to investigate the interaction of tested compounds with HEWL as described previously.^40^ Briefly, aliquots of 50 μM protein, prepared in 50 mM glycine buffer (pH 2.2), were incubated without or with 1, 5, and 10 μM compounds 3d and 3e for 1 min. Then, 50 μM NR was added to the mixtures and the maximum emission of samples at 641 nm was collected using an excitation wavelength of 555 nm. All measurements were performed at room temperature. The steady-state anisotropy was calculated according to Nigen *et al.*^42^ All fluorescence experiments were carried out on a Cary Eclipse VARIAN fluorescence spectrophotometer (Mulgrave, Australia).

#### Congo red binding assay

Congo red (CR) binding assay was employed as an alternative assay for amyloid fibril detection. Aliquots of incubated solutions containing different concentrations of compounds 3d and 3e were mixed with 20 μM CR solution (made from a 5 mM CR stock solution in 5 mM potassium phosphate and 0.15 M sodium chloride buffer, pH 7.4). The HEWL final concentration was 2.5 μM. The mixtures were then incubated at room temperature for 30 min followed by recording the absorbance spectra of samples between 400 and 600 nm.

#### Atomic force microscopy

To study the effects of compounds 3d and 3e on the morphology of HEWL aggregates, incubated samples were diluted 50-fold by deionized water and 10 μL of diluted samples were put on clean mica and allowed to dry at room temperature. Images were taken using quantitative Atomic Force Microscopy (ARA-AFM) in non-contact mode, and imager software was used to process the images.

### Cell culture and MTT assay

The human neuroblastoma SH-SY5Y cells were cultured in DMEM-F12 medium as reported previously.^43^ The culture medium was replaced three times a week. The cells were seeded into a 96-well plate at a density of 2 × 10^4^ cells per well and the medium was changed prior to incubation with HEWL aggregates. To evaluate possible cytotoxicity of tested compounds, cells were exposed to increasing concentrations (0–200 μM) of compounds 3d and 3e, followed by incubation for 24 h. To assess the effect of compounds against cytotoxicity induced by HEWL aggregates, cells were treated with protein aggregates aged alone or in the presence of various concentrations of tested compounds for 24 h. Cells treated with 50 mM glycine buffer (pH 2.2) were used as control. The conventional MTT reduction assay was used to evaluate viability of cells. Briefly, 10 μL of MTT stock solution (5 mg mL^−1^ in PBS) was added to each well and incubated for 4 h. The solutions were then aspirated and the cells were treated with DMSO for 15 min, followed by measuring absorbance at 570 nm using an ELISA reader (Expert 96, Asys Hitch, Ec Austria). The results were expressed as the percentage of MTT reduction compared to the control cells, with the assumption that the absorbance of control cells was 100%. All measurements were made in triplicates.

### Hemolysis assay

To evaluate possible hemolytic activity of new synthesized 3d and 3e compounds, the suspensions of erythrocyte (∼1% hematocrit) were treated with various concentrations (0–200 μM) of 3d or 3e compounds for 3 h at 37 °C while being gently stirred. Triton X-100 (at a final concentration of 0.1% v/v) was used as positive control. The samples were centrifuged at 1000*g* for 10 min and the absorbance of supernatants at 540 nm, as indicative of hemoglobin, was measured. The percentage of hemolysis was calculated as a fraction of Triton X-100.^44^ All measurements were carried out in triplicate. At the end of experiment, a drop of resultant pellets was deposited on a clean glass slide and air-dried. The images were acquired using an optical microscope (Nikon, Japan) at 20× magnification.

### Molecular docking study

For molecular docking calculations, the structures of synthesized compounds 3d and 3e were first drawn in ACD/Chemsketch (V. 2018.2.1)^45^ and then their pdb formats were generated using Online Open Babel Software.^46^ The crystal structure of HEWL monomer was obtained from the Protein Data Bank (PDB ID: 3WUN). Prior to docking, all water molecules were removed from the PDB file. Using Auto Dock tools 1.5.6 Program, all hydrogen atoms were also added to the protein, and Kollman and Gasteiger charges were used for the ligands and HEWL, respectively. Throughout the dockings, the protein molecule was set to be rigid while the ligand molecules were considered to be flexible. The grid volume was arranged big enough to cover the entire surface of the protein. A total of 100 runs were performed to more accurately find the most appropriate binding sites possessing the lowest binding energies (calculated using the Autodock scoring function). After verifying the residues lining the binding sites (Trp62, Trp63, and Trp108), they were set flexible for another 250 runs of molecular dockings to further define the role of protein residues involved in the interaction with ligands. Finally, the ligands' positions with the lowest binding energy scores were selected as the best binding modes to HEWL. The secondary-structure images were created using VMD 1.9.3.^47^

### Statistical analysis

The experiments were conducted two or three times with triplicate repetitions. The results are presented as a percentage relative to untreated control cells, where each value represents the average ± standard deviation (*n* = 3). The significant differences between the means of treated and untreated groups were calculated by unpaired Student's *t*-test and *p*-values less than 0.05 were considered significant. *p < 0.05 and **p < 0.01 were significantly different from control. ^#^*p* < 0.05 and ^##^*p* < 0.01 were significantly different from cells exposed solely to HEWL amyloid fibrils.

## Results and discussion

### Synthesis of hydroxycarbamoyl phosphinic acid derivatives

**Scheme 1:**
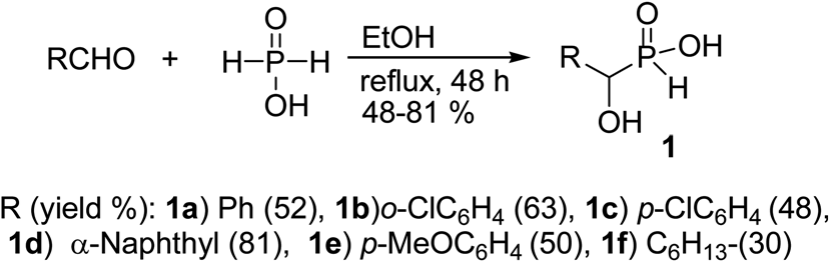
Synthesis of 1-hydroxyalkylphosphinic acids 1.

1-Hydroxyalkylphosphinic acids 1 were obtained in gram scale from the reaction of aldehyde and hypophosphorus acid at reflux ethanol for 48 h (Scheme 1), according to a literature procedure.^48^

**Scheme 2:**
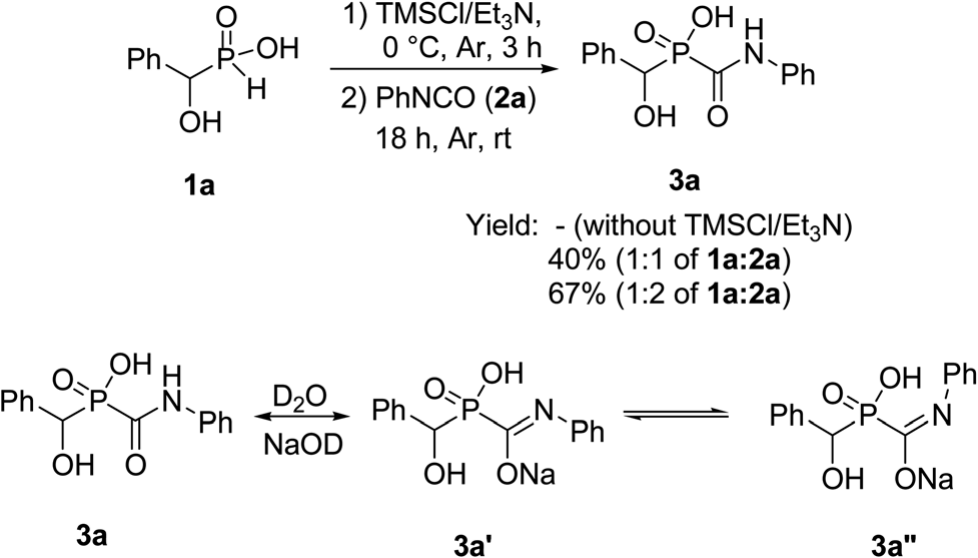
Synthesis of 1-hydroxcarbamoylphosphinic acids 3a.

Treatment of 1a with 2a was chosen as a model and the data of the conditions are shown in Scheme 2.

For the first attempt the reaction of 1a with 2a in toluene at room temperature or reflux condition failed to give compound 3a after 24 h. Treatment of 1a with 2a in the presence of TMCl/Et_3_N in toluene for 18 h at room temperature, gave 3a in 40% isolated yield. Reaction yield increased to 67% when the reaction was carried out with excess of the compound 2a. The ^31^P-NMR spectrum of this mixture in DMSO-d_6_ exhibited on peak at *δ* 22.43 ppm. The ^1^H-NMR spectrum exhibited one doublet at *δ* 5.19 ppm indicative of N-HC-P coupling and the ^13^C-NMR of the compound showed a doublets peak (^1^*J*_CP_ = 148 Hz) at 170 ppm due to the presence of C=O group that directly connected to phosphorus atom. It should be noted that when the compound NMR analysis carried out in D_2_O-NaOD, the ^31^P-NMR spectrum exhibited two peaks at *δ* 17.57 and 19.14 ppm due to two forms (3a′ and 3a′′ in Scheme 2). The ^1^H-NMR spectrum exhibited two doublets at *δ* 4.80–4.90 ppm indicative of N-HC-P coupling of two forms due to the presence of resonance in amide part. This process was successfully applied to other α-hydroxy-*H*-phosphinic acids (1) and isocyanates or isothiocynates (2) as summarized in Table 1. As shown in Table 1, the reaction of α-hydroxyalkylphosphinic acids (1a–1e) with isocyanates and isothiocyanates (2a–2f), in the presence of TMSCl/Et_3_N, afforded the corresponding 1-hydroxycarbamoyl (or thiocarbamoyl) phosphinic acid in moderate to good yields (3a–3k).

**Table 1:** Reaction of 1-hydroxyalkylphosphinic acids 1 with isocyanates and isothiocynates 2

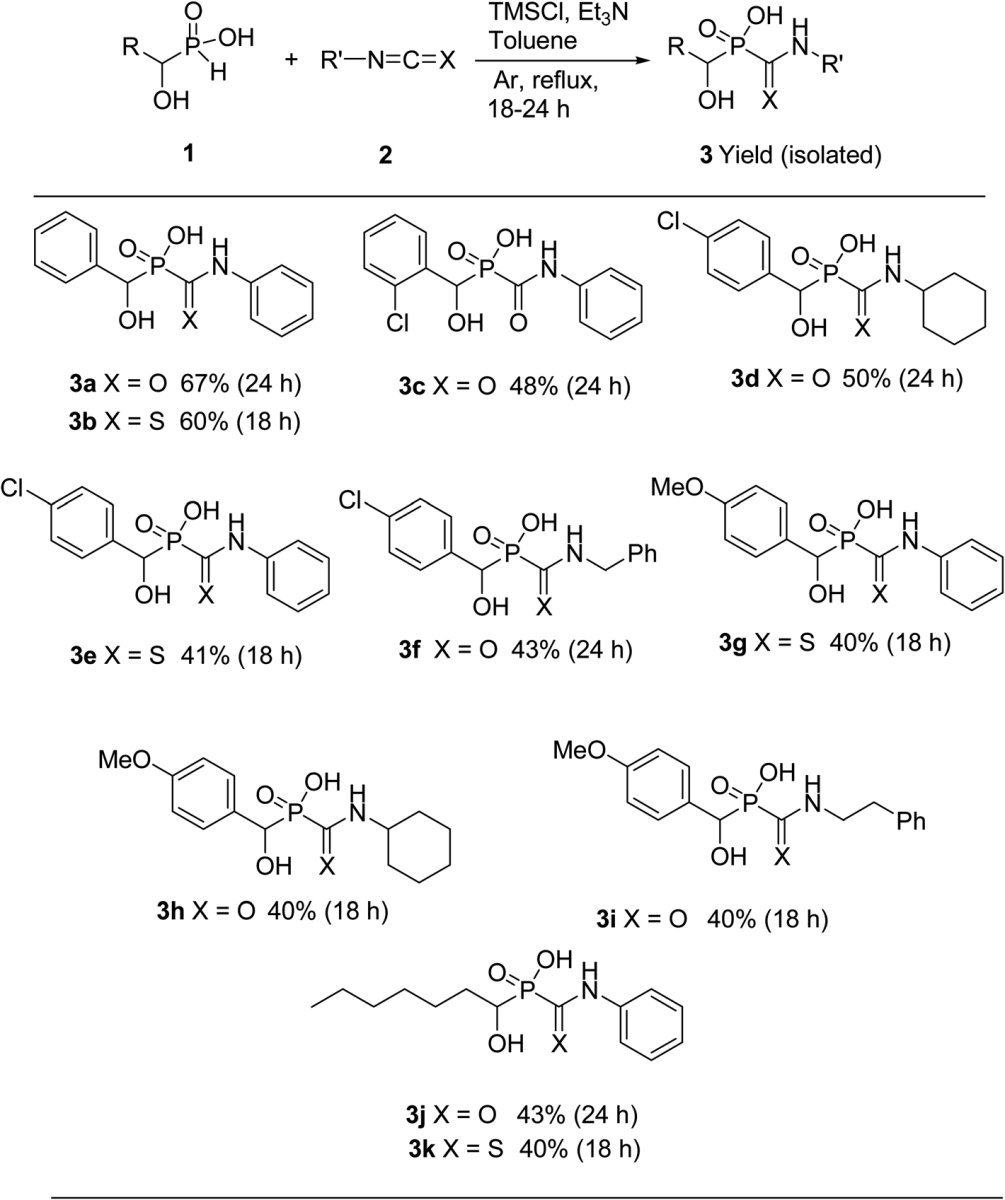

### Effect of compounds 3d and 3e on HEWL amyloid fibril formation

**Fig. 1:**
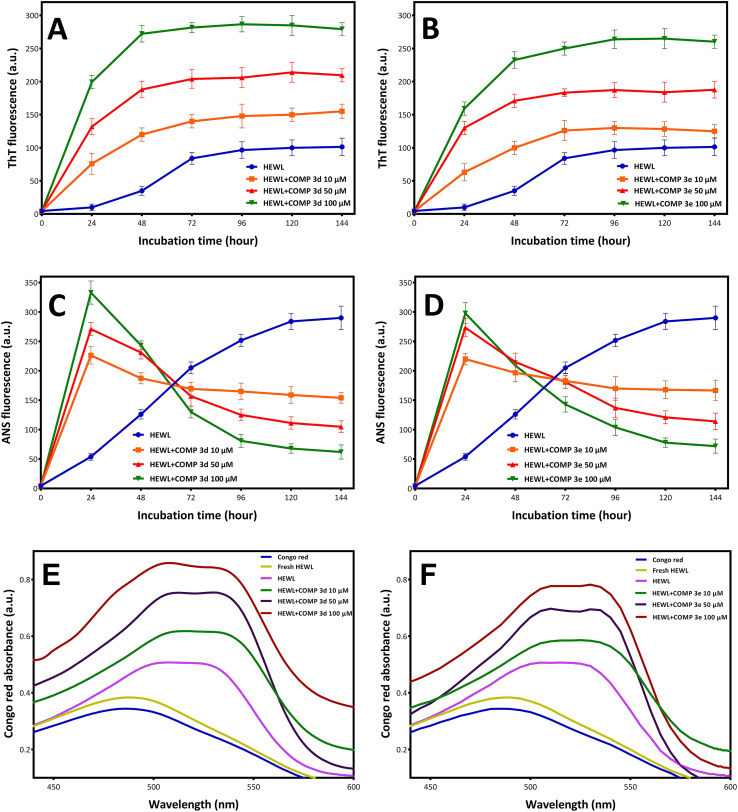
Effect of compounds 3d and 3e on the HEWL amyloid fibrillation. Protein samples (100 μM) were incubated under amyloidogenic conditions without or with increasing concentrations of compound 3d and 3e for 6 days. (A and B) Kinetics of HEWL fibrillation indicated by increasing fluorescence intensity of ThT at specified concentrations of compounds 3d and 3e, respectively. (C and D) Changes in the surface hydrophobicity of HEWL indicated by increasing fluorescence intensity of ANS at specified concentrations of compounds 3d and 3e, respectively. (E and F) Changes in the Congo red binding absorption spectra of HEWL at specified concentrations of compounds 3d and 3e, respectively. Every test was conducted three times and the average was used.

The synthesis and purity of hydroxycarbamoyl phosphinic acid derivatives were confirmed by a range of techniques including NMR, LC-MS, and HPLC (ESI†). To evaluate the potential of new synthesized compounds on modulating fibrillation of HEWL, two compounds 3d and 3e were selected randomly. The fibrillation kinetics of HEWL in the presence of different concentrations (10, 50, and 100 μM) of compounds 3d and 3e was assessed using ThT fluorescence assay, showing a notable dose-dependent increase in ThT fluorescent intensity compared to the control group (Fig. 1A and B).

Interestingly, the presence of both compounds resulted in disappearance of nucleation phase and increasing the rate of growth phase in a concentration-dependent manner (Fig. 1A and B). These observations indicate that both compounds 3d and 3e have the capacity to modulate fibrillogenesis of HEWL by shortening of the nucleation phase leading to acceleration of HEWL self-assembly process. Based on these observations, we may suggest that both compounds likely target partially unfolded species or small oligomers, but not monomeric species, of HEWL formed during the nucleation phase. The capacity of different compounds in targeting various intermediates (including monomers, partially unfolded species, small oligomers, and protofibrils) formed in the course of fibril formation is largely associated with their structural properties. For example, Xu *et al.*^49^ reported that the small molecule *YX*-I-1 (with relatively high aqueous solubility) can delay the aggregation of hIAPP by targeting monomers leading to prolongation of nucleation phase. On the other hand, the small molecule *YX*-A-1 (with low aqueous solubility) accelerates the assembly process of hIAPP by binding to oligomeric species formed during the nucleation phase. In accord with this, other studies have reported that small molecules with the ability to target hydrophobic regions of amyloidogenic species can accelerate fibrillogenesis by favoring hydrophobic interactions.^16^ These findings suggest that the structural properties of various compounds (including their hydrophobicity/hydrophilicity) may be an important parameter that determine their interactions with protein, as well as, the possible mechanism of action. As shown in Table 1, both 3d and 3e compounds contain aromatic rings showing high levels of hydrophobicity. Accordingly, we propose that the ability of compounds 3d and 3e to target and bind to hydrophobic regions, exposed during the initial steps of HEWL aggregation, may underlies the mechanism by which these compounds promote fibrillogenesis of HEWL.

To further corroborate this proposition, changes on the fluorescence emission of ANS was examined for the incubated samples. ANS is a fluorescent dye that shows low fluorescence quantum yield in water, but when binds to hydrophobic clusters its fluorescence intensity increases significantly,^50–52^ and can be used for characterization of proteins aggregates relevant to neurodegenerative diseases.^53^ As shown in Fig. 1C and D, for protein samples incubated with compounds 3d and 3e, a quick and notable rise in ANS fluorescence intensity was observed, followed by a significant decrease in its fluorescence emission. A possible explanation for this observation may be related to high hydrophobicity of 3d and 3e compounds and their capacity to target partially unfolded species, with solvent-exposed hydrophobic regions, formed during the initial stages of aggregation. On the other hand, binding of compounds 3d and 3e can increase surface hydrophobicity required for promoting association of hydrophobic regions of HEWL oligomers leading to acceleration of amyloid fibril formation. Upon growth of oligomers and formation of mature fibrils, the lateral interactions between fibrillar structures can result in the formation of fibrillar bundles of HEWL which may restrict the access of ANS to hydrophobic regions and result in the decreased fluorescence of dye (Fig. 1C and D).

Congo red (CR) is a diazo dye that selectively binds to fibrillar structures with a high proportion of β-sheet content, and frequently utilizes as an alternative technique for amyloid fibril detection.^54^ As depicted in Fig. 1E and F, incubation under amyloidogenic condition resulted in an increase in CR absorbance with a red shift, an indicative of amyloid fibril formation. Treatment with compounds 3d and 3e notably increased CR absorbance dose-dependently, with the appearance of a second shoulder peak at around 540 nm (Fig. 1E and F), indicating a strong binding affinity between CR and HEWL fibrils and signifying the presence of substantial amounts of amyloid fibrils.^55^

To further confirm the promoting effect of tested compounds, the concentration of 6 day-old amyloid fibrils produced in the absence and presence of increasing concentrations of compounds 3d and 3e was determined according to the protocol described by Meratan *et al.*^41^ As shown in Table 2, treatment with both compounds 3d and 3e has resulted in a significant enhancement in amyloid fibril concentration in a dose-dependent manner.

**Table 2:** Effect of compounds 3d and 3e on the formation of HEWL amyloid fibrils. Protein samples were incubated under amyloidogenic conditions without or with increasing concentrations of compound 3d and 3e for 6 days followed by centrifugation at 21 000*g* for 30 min. Then, the concentration of supernatant (corresponding to oligomers/protofibrils) and pellet (corresponding to amyloid fibrils) was determined as described in the Materials and methods section

	Supernatant (μM)	Pellet (μM)
HEWL	68.74 ± 0.78	31.26 ± 1.56
HEWL + COMP 3d 10 μM	51.29 ± 1.65	48.71 ± 1.14
HEWL + COMP 3d 50 μM	38.66 ± 2.22	61.34 ± 0.89
HEWL + COMP 3d 100 μM	23.45 ± 1.40	76.55 ± 2.04
HEWL + COMP 3e 10 μM	56.78 ± 2.25	43.22 ± 1.18
HEWL + COMP 3e 50 μM	44.82 ± 1.46	55.18 ± 1.71
HEWL + COMP 3e 100 μM	32.38 ± 1.90	67.62 ± 1.92

**Fig. 2:**
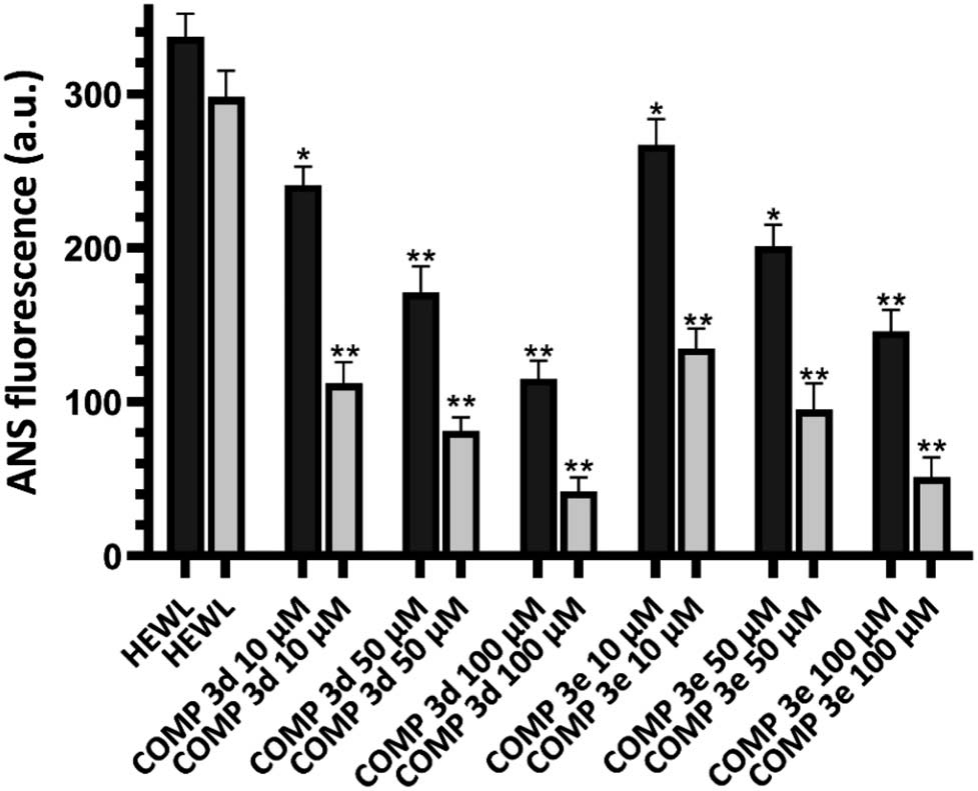
Effect of increasing concentrations of compounds 3d and 3e on the surface hydrophobicity of HEWL aggregates incubated for 6 days. The incubated solutions were centrifuged at 21 000*g* for 30 min and changes in the surface hydrophobicity of supernatant (dark bars) and pellet (white bars) was measured by ANS fluorescence assay. *p < 0.05, **p < 0.01, significantly different from control samples.

While for the control sample the concentration of HEWL fibrils was 31.26 μM, the presence of 100 μM of compounds 3d and 3e significantly increased the concentration of HEWL aggregates to 76.55 μM and 67.62 μM, respectively (Table 2). Fig. 2 shows the surface hydrophobicity of supernatant (corresponding to oligomeric species/protofibrils) and pellet (corresponding to amyloid fibrils/insoluble aggregates) of 6 day-old aggregates, measured by ANS fluorescence assay.

While for control sample, both supernatant and pellet exhibited a high level of ANS fluorescence intensity, a dose-dependent decrease in the surface hydrophobicity of supernatant and pellet was observed for the samples treated with compounds 3d and 3e. Furthermore, the extent of ANS fluorescence decrease was more significant for pellet corresponding to amyloid aggregates (Fig. 2). These results suggest that the presence of compounds 3d and 3e not only accelerates the rate of amyloid fibrillation, but also leads to the formation of mature fibrils with decreased surface hydrophobicity, which are in accord with results presented in Fig. 1.

**Fig. 3:**
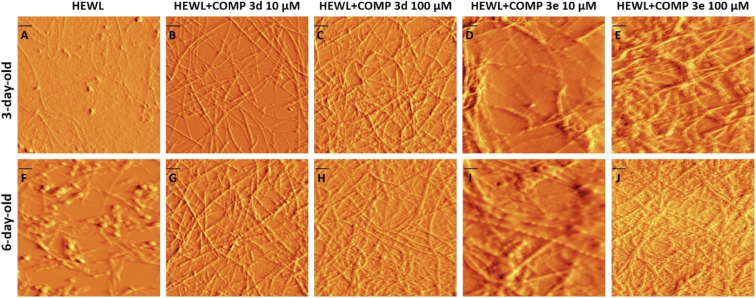
AFM images of HEWL incubated under amyloidogenic conditions without or with increasing concentrations of compounds 3d and 3e for 3 and 6 days. The scale bar represents 100 nm.

In the next step, the morphology of protein aggregates formed alone or in the presence of various concentrations of compounds 3d and 3e, at two different time intervals of 3 and 6 days, was analyzed using AFM imaging. Fig. 3A–E show the AFM images of protein samples incubated alone or in the presence of increasing concentrations of compounds 3d or 3e for 3 days (corresponding to the end of growth phase of control sample), indicating the appearance of well-formed mature fibrils only for the samples treated with compounds. For control sample (Fig. 3A), we observed a mixture of globular intermediate and protofibrils. On the other hand, acceleration of HEWL fibril formation mediated by compounds 3d and 3e has decreased the concentration of HEWL soluble oligomers and protofibrils.

To further corroborate promoting effect of compounds 3d and 3e, the AFM images of aggregates were acquired after 6 days, corresponding to the plateau phase of control sample (Fig. 1). As depicted in Fig. 3G–J, incubation of protein samples in the presence of compounds 3d and 3e for 6 days resulted in the formation of more crowded, well-formed fibrillar structures. For control samples, we observed a mixture of fibrils and amorphous aggregates (Fig. 3F). Based on the results obtained by ThT and ANS fluorescence assays, CR binding measurement, and AFM analysis, we can conclude that both compounds 3d and 3e are able to modulate conformation of HEWL oligomers, thereby increasing solvent-exposed hydrophobic regions of amyloidogenic species leading to acceleration of HEWL fibrillogenesis. These results are in accord with previous studies indicating that accelerators of amyloid fibrillation predominantly interact with species showing high level of surface hydrophobicity formed during the lag phase.^16,49^

### Effect of compounds 3d and 3e on the cytotoxicity of HEWL aggregates

**Fig. 4:**
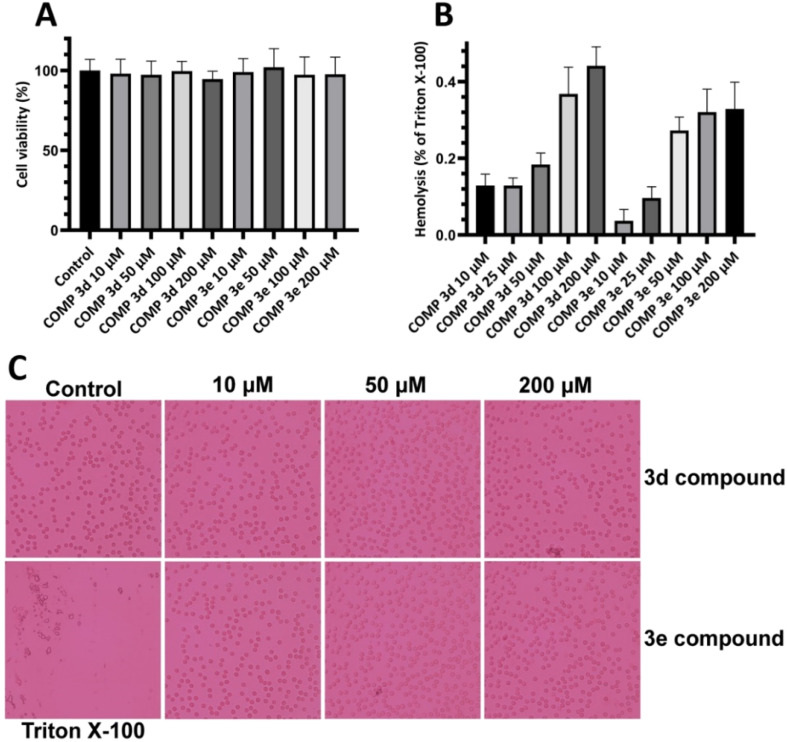
Effect of increasing concentrations of compounds 3d and 3e on (A) viability of SH-SY5Y cells and (B) erythrocyte membrane integrity indicating no significant cytotoxicity. (C) Optical microscopy images of erythrocytes incubated with increasing concentrations of compounds 3d and 3e. Further details are provided in the Materials and methods section.

First, we performed MTT-based cell viability assay to evaluate the possible cytotoxicity of varying concentrations of compounds 3d and 3e ranging from 10 to 200 μM. Our results didn't reveal any notable cytotoxicity when compared to the control cells (Fig. 4A).

**Fig. 5:**
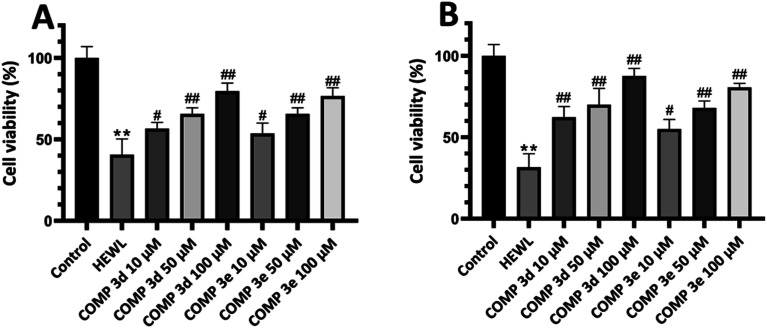
Effect of compounds 3d and 3e on the HEWL amyloid cytotoxicity evaluated by MTT assay. (A and B) Cytotoxicity evaluation of HEWL aggregates produced in the absence or presence of increasing concentrations of compounds 3d and 3e after 3 and 6 days, respectively. **p < 0.01, significantly different from control cells. #*p* < 0.05, ##*p* < 0.01, significantly different from cells exposed only to HEWL amyloid fibrils.

To further confirm these results and evaluate their effects on biological membranes, the effects of compounds 3d and 3e on the integrity of erythrocyte membrane was investigated. As shown in Fig. 4B, treatment of erythrocytes with increasing concentrations of tested compounds didn't lead to any membrane permeabilization. Optical microscopy images of treated samples further confirmed the non-toxicity of compounds 3d and 3e (Fig. 4C). In the next step, the cytotoxicity of HEWL amyloidogenic species, produced after 3- and 6 days incubation in the absence or presence of various concentrations of compounds 3d or 3e, was examined. In accord with our recent report,^56^ 10 μM protein aggregates was used for cytotoxicity experiments. According to the results showed in Fig. 5A, 3 day-old amyloid aggregates aged in the presence of tested compounds exhibited a concentration-dependent increase in cell viability compared to protein samples incubated alone. Similar results were obtained for 6 day-old aggregates (Fig. 5B), indicating diminished cytotoxicity of aggregates produced in the presence of compounds 3d and 3e.

**Fig. 6:**
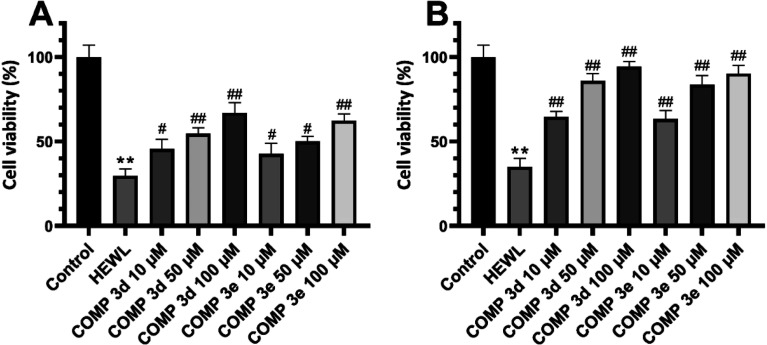
Cytotoxicity evaluation of (A) supernatant and (B) pellet of HEWL aggregates incubated for 6 days in the absence or presence of increasing concentrations of compounds 3d and 3e. **p < 0.01, significantly different from control cells. #*p* < 0.05, ##*p* < 0.01, significantly different from cells exposed only to HEWL amyloid fibrils.

Fig. 6 shows the MTT-based cell viability assay of supernatant and pellet of 6 day-old aggregates incubated without or with various concentrations of tested compounds, indicating the increased viability of cells. This reduced cytotoxicity may be correlate with diminished hydrophobicity of HEWL oligomers and mature fibrils formed in the presence of varying concentrations of compounds 3d and 3e (Fig. 2).

Moreover, the extent of protection was more pronounced in samples treated with pellet containing mature amyloid fibril (Fig. 6B), with lower content of surface hydrophobicity (Fig. 2). Based on the cell viability results, we may suggest that acceleration of HEWL fibrillogenesis by tested compounds has resulted in the formation of aggregates with reduced cytotoxicity.

Increasing body of evidence indicates that cytotoxicity associated with amyloid fibrils is dependent not only on the chemical composition and surface properties of amyloid fibrils, but also their physical attributes such as physical dimensions and surface area are suggested to play important roles.^57,58^ For instance, Xue *et al.*^59^ reported a direct correlation between reduced size of fibrils and their ability to disrupt membrane integrity and cause cell death. In another study, Zhang *et al.*^60^ found that the uptake and cellular toxicity of α-synuclein fibrils showed a reverse correlation with the size of amyloid fibrils; larger fibrils caused lower cytotoxicity. Accordingly, we may propose that formation of large amyloid fibrils in the present of tested compounds has decreased their capacity to interact with cell membrane and cause cytotoxicity in SH-SY5Y cells (Fig. 5). In addition to physical features, surface hydrophobicity is considered as another crucial factor in the process of fibril formation and cytotoxicity.^61^ Therefore, the decreased cytotoxicity of structures produced in the presence of tested compounds may attribute to their diminished surface hydrophobicity (Fig. 1C and D). On the other hand, the significant cytotoxicity of control samples may be related to the presence of globular intermediate and protofibrils (Fig. 3A and F) with high levels of surface hydrophobicity (Fig. 1C and D), as main toxic species in the course of protein fibrillation.^62–64^ In accord with this conclusion, Kreiser *et al.* reported that the cytotoxicity of oligomeric intermediates is determined by their size and solvent-exposed hydrophobic patches, with higher levels of hydrophobicity and smaller sizes being associated with a greater ability to cause cellular dysfunction.^65^ This size- and surface hydrophobicity-dependent toxicity of amyloidogenic intermediates has been reported by several studies.^65–69^ Thus, we suggest that decreased cytotoxicity in protein samples produced in the presence of tested compounds may be related to their capacity in accelerating assembly process of HEWL leading to reduced concentration of toxic intermediates with the formation of thick, mature fibrils (Fig. 3) with limited hydrophobic patches (Fig. 1C and D).

### Characterization of HEWL-compounds 3d and 3e interaction

Fluorescence anisotropy and molecular docking were utilized to gain further insights into the interaction of compounds 3d and 3e with HEWL. First, fluorescence anisotropy was employed to investigate the interaction between compounds 3d and 3e with NR-labeled HEWL. As shown in Table 3, the fluorescence anisotropy value of NR-labeled HEWL in the absence of compounds is 0.4283 ± 0.0129.

**Table 3:** Anisotropy of NR-labeled HEWL before and after addition of various concentrations of compounds 3d and 3e

	Measured anisotropy
HEWL	0.4283 ± 0.0129
HEWL + COMP 3d 1 μM	0.3146 ± 0.0184
HEWL + COMP 3d 5 μM	0.2366 ± 0.0090
HEWL + COMP 3d 10 μM	0.1930 ± 0.0061
HEWL + COMP 3e 1 μM	0.2823 ± 0.0062
HEWL + COMP 3e 5 μM	0.2303 ± 0.0136
HEWL + COMP 3e 10 μM	0.2053 ± 0.0091

**Fig. 7:**
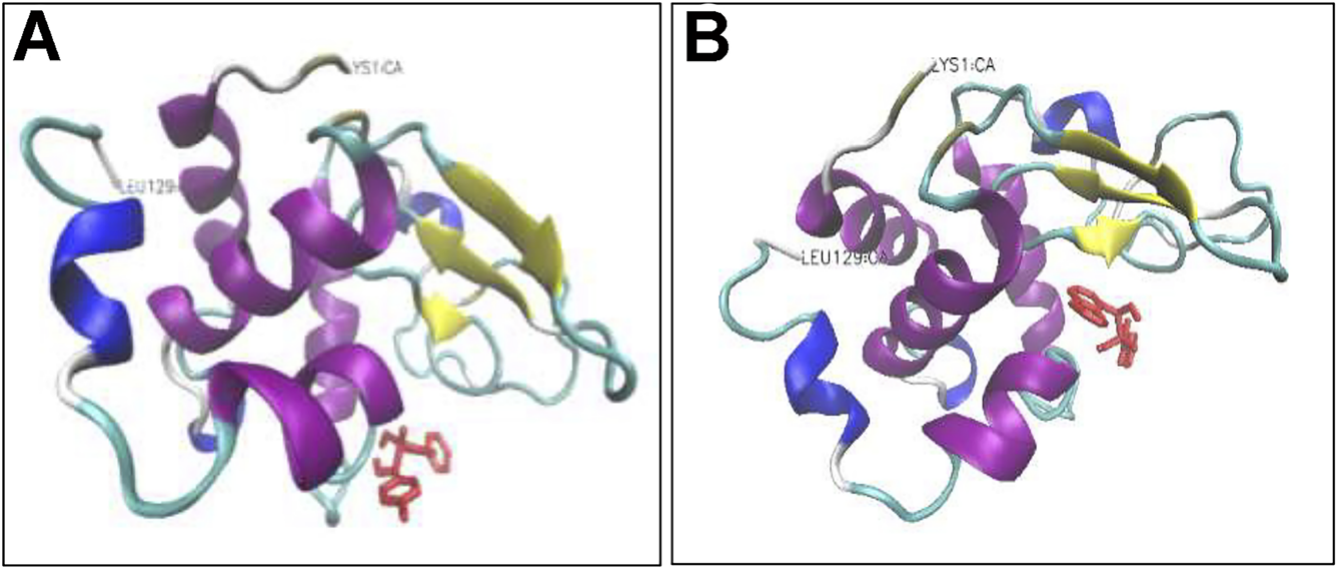
Binding modes of compounds 3d and 3e to HEWL. Compounds 3d (A) and 3e (B) are interacting with HEWL active site. The compounds are depicted in red in stick model. Protein backbone of HEWL is shown in cartoon model. The secondary structure of HEWL is depicted as follows: β-strand: yellow; α-helix: purple; 3/10 helix: blue; random coil: white; turn: cyan. The N- and C-termini of HEWL are displayed as LYS1 and LEU129, respectively.

Addition of either compounds 3d or 3e led to a dose-dependent decrease in the measured anisotropy, suggesting the interaction of tested compounds with protein. A possible explanation for this decreased anisotropy may be related to new conformation of protein induced by tested compounds, in which energy transfer occurs between two or more probes.^70^ To further characterize the interaction between HEWL and compounds 3d and 3e, molecular docking was performed. Through the docking runs, the best binding energy scores of −6.54 and −6.27 kcal mol^−1^ were obtained for the compounds 3d and 3e, respectively, when interacting with the α and β domains of HEWL active site^62,63^ (Fig. 7), indicating the ability of these compounds to bind HEWL.

**Fig. 8:**
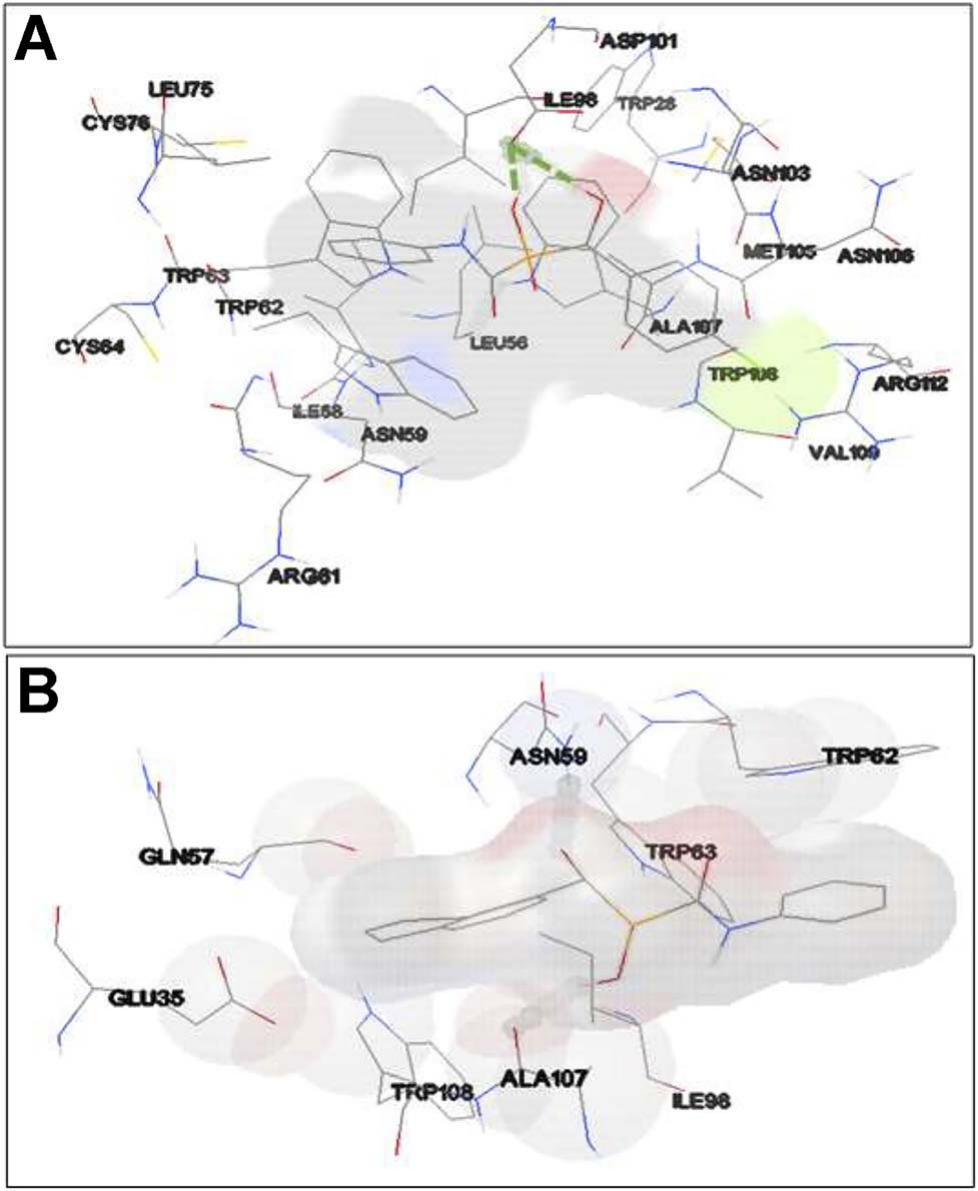
Binding site of compounds 3d and 3e within HEWL. HEWL residues surrounding compounds 3d (A) and 3e (B) are shown with numbers. Hydrogen bonds within 1 Å are represented as dashed green lines.

As shown in Fig. 8A, in the binding site, compound 3d is surrounded by nine residues of HEWL β-domain (Leu56, Ile58, Asn59, Arg61-Cys64, Leu75, and Cys76) and by nine residues of HEWL α-domain (Ile98, Asp101, Asn103, Met105-Val109, and Arg112).

The compound 3d also formed two hydrogen bonds with Ile98 of HEWL α-domain within the active site. Meanwhile, the binding site of compound 3e includes four residues of HEWL β-domain (Gln57, Asn59, Trp62, and Trp63) and three residues of HEWL α-domain (Ile98, Ala107, and Trp108) (Fig. 8B). In its binding site, the compound 3e made two hydrogen bonds with Asn59 and Ala107. The β-domain of HEWL has been reported as the aggregation-prone region of the protein for amyloid fibril formation.^71–73^ Interestingly, both compounds exhibited displacement in this aggregation-prone region, indicating the potency of these compounds to bind and modulate self-assembly process of HEWL. Based on the ThT and ANS data (Fig. 1), we have suggested that compounds 3d and 3e likely target soluble oligomers/protofibrils with increased solvent-exposed hydrophobic regions, produced in the initial steps of HEWL aggregation. Therefore, the absence of hydrophobic interactions between HEWL and compounds 3d and 3e (Fig. 8) may be attributed to the high levels of hydrophilicity of HEWL monomer utilized for the molecular docking studies. On the other hand, significant binding energy scores of −6.54 and −6.27 kcal mol^−1^ obtained for the compounds 3d and 3e, respectively (Fig. 7), suggest that the compounds have made efficient bindings with the crucial domains of the HEWL monomer and the binding were effectively remained until oligomers and protofibrils formed in the initial steps of HEWL self-assembly.

## Conclusion

**Scheme 3:**
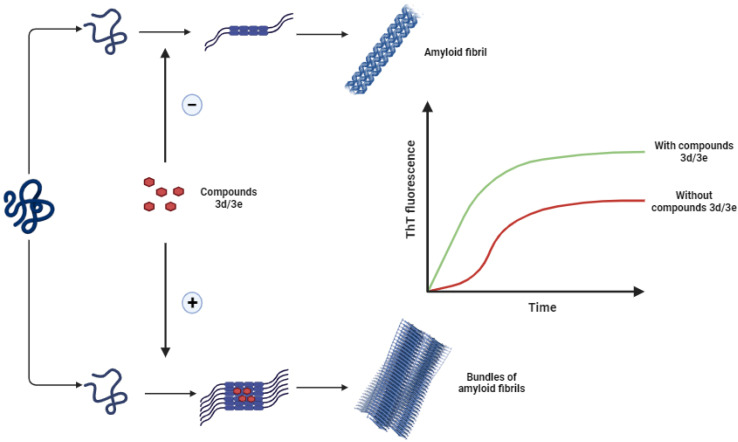
Schematic representation of proposed mechanism by which compounds 3d/3e modulate HEWL fibril formation leading to acceleration of assembly process of protein.

The main objective of the present study was to investigate the effect of some new synthesized 1-hydroxycarbamoyl phosphinic acids derivatives in the aggregation process of HEWL and to determine their possible mechanism of action. Herein, for the first time we showed the potency of compounds 3d and 3e to act as accelerator of HEWL amyloid fibrillation, determined by ThT and ANS fluorescence assays, CR binding measurement, and AFM analysis. Cytotoxicity and hemolysis assays showed decreased toxicity of structures produced in the presence of these compounds. Moreover, fluorescence anisotropy and molecular docking analysis indicated the interaction of compounds 3d and 3e with the aggregation-prone β-domain of HEWL thereby modulating protein aggregation. We suggest that through targeting hydrophobic regions of HEWL exposed in the initial steps of aggregation, compounds 3d and 3e can increase surface hydrophobicity of HEWL amyloidogenic species and promote the association and growth of toxic soluble oligomers leading to formation of large aggregates with mitigated toxicity (Scheme 3). The ability of compounds 3d and 3e to accelerate the formation of non-toxic mature fibrils while reducing the presence of toxic intermediates marks a promising avenue for future research and development of therapeutic approaches relating to neurodegenerative diseases.

## Data availability

The data that support the findings of this study are available from the corresponding author upon reasonable request.

## Author contributions

A. A. M. conceived of the project and designed the experiments; M. M. conducted experiments and wrote the primary draft; F. T. and Z. M. E. conducted fluorescence anisotropy and hemolysis assay; S.A. synthesized α-hydroxycarbamoyl phosphinic acids; M. G. and E. T. conducted HPLC analysis; T. Z. and Y. G. conducted LC-MS experiments; M.-B. E.-H. conducted molecular docking experiments; B. K. supervised synthesis of α-hydroxycarbamoyl phosphinic acids and analyzed NMR data; B. K. and A. A. M. supervised the project and edited the manuscript; all authors read the manuscript and commented on it.

## Conflicts of interest

The authors declare that they have no conflict of interest.

## Supplementary Material

RA-014-D4RA02969K-s001
