# Supplementary material for: Inhibition of cytotoxic self-assembly of HEWL through promoting fibrillation by new synthesized α-hydroxycarbamoylphosphinic acids
Source: RSC Adv. 2024 Oct 1;14(42):31227–42. doi: 10.1039/d4ra02969k (PMC11443501; doi:10.1039/d4ra02969k)
Supplement: RA-014-D4RA02969K-s001 [file RA-014-D4RA02969K-s001.pdf]

Supplementary Information (SI) for RSC Advances.
This journal is © The Royal Society of Chemistry 2024

Supporting Information

Inhibition of Cytotoxic Self-assembly of HEWL Through Promoting Fibrillation by New Synthesized α-Hydroxycarbamoylphosphinic Acids

Mohsen Mahdavimehr[a], Babak Kaboudin[b*], Saied Alaie[b], Farimah Tondkar[a], Zahra Mahmoudi Eshkaftaki[a], Mohammad-Bagher Ebrahim-Habibi[c], Mojtaba Ghashghaee[b], Elham Tahmasebi[b], Tianjian Zhang[d], Yanlong Gu[d], Ali Akbar Meratan[a*]

[1] Department of Biological Sciences, Institute for Advanced Studies in Basic Sciences (IASBS), Zanjan, 45137-66731, Iran

[2] Department of Chemistry, Institute for Advanced Studies in Basic Sciences (IASBS), Zanjan 45137-66731, Iran

[3] Microbiology and Biotechnology Group, Research Institute of Petroleum Industry, Tehran, Iran

[4]School of Chemistry and Chemical Engineering, Huazhong University of Science & Technology, Wuhan 430074, China

[*] Corresponding authors

Babak kaboudin; E-mail: kaboudin@gmail.com

Ali Akbar Meratan; E-mail: a.meratan@iasbs.ac.ir; a.meratan@gmail.com

Table of Contents

NMR Spectra for the compounds 3a-3n **S1-S33**

Figure S1: ^{1}H NMR Spectra of 3a

Figure S2: ^{13}C NMR Spectra of 3a

Figure S3: ^{31}P NMR Spectra of 3a

Figure S4: ^{1}H NMR Spectra of 3b

Figure S5: ^{13}C NMR Spectra of 3b

Figure S6: ^{31}P NMR Spectra of 3b

Figure S7: ^{1}H NMR Spectra of 3c

Figure S8: ^{13}C NMR Spectra of 3c

Figure S9: ^{31}P NMR Spectra of 3c

Figure S10: ^{1}H NMR Spectra of 3d

Figure S11: ^{13}C NMR Spectra of 3d

Figure S12: ^{31}P NMR Spectra of 3d

Figure S13: ^{1}H NMR Spectra of 3e

Figure S14: ^{13}C NMR Spectra of 3e

Figure S15: ^{31}P NMR Spectra of 3e

Figure S16: ^{1}H NMR Spectra of 3f

Figure S17: ^{13}C NMR Spectra of 3f

Figure S18: ^{31}P NMR Spectra of 3f

Figure S19: ^{1}H NMR Spectra of 3g

Figure S20: ^{13}C NMR Spectra of 3g

Figure S21: ^{31}P NMR Spectra of 3g

Figure S22: ^{1}H NMR Spectra of 3h

Figure S23: ^{13}C NMR Spectra of 3h

Figure S24: ^{31}P NMR Spectra of 3h

Figure S25: ^{1}H NMR Spectra of 3i

Figure S26: ^{13}C NMR Spectra of 3i

Figure S27: ^{31}P NMR Spectra of 3i

Figure S28: ^{1}H NMR Spectra of 3j

Figure S29: ^{13}C NMR Spectra of 3j

Figure S30: ^{31}P NMR Spectra of 3j

Figure S31: ^{1}H NMR Spectra of 3k

Figure S32: ^{13}C NMR Spectra of 3k

Figure S33: ^{31}P NMR Spectra of 3k

LC-MS Spectra for the compounds 3a-3k **S34-S44**

Figure S34: LCMS of 3a

Figure S35: LCMS of 3b

Figure S36: LCMS of 3c

Figure S37: LCMS of 3d

Figure S38: LCMS of 3e

Figure S39: LCMS of 3f

Figure S40: LCMS of 3g

Figure S41: LCMS of 3h

Figure S42: LCMS of 3i

Figure S43: LCMS of 3j

Figure S44: LCMS of 3k

Figure S45: **HPLC-UV chromatograms of 50 mg L^{-1} solutions of the compounds**

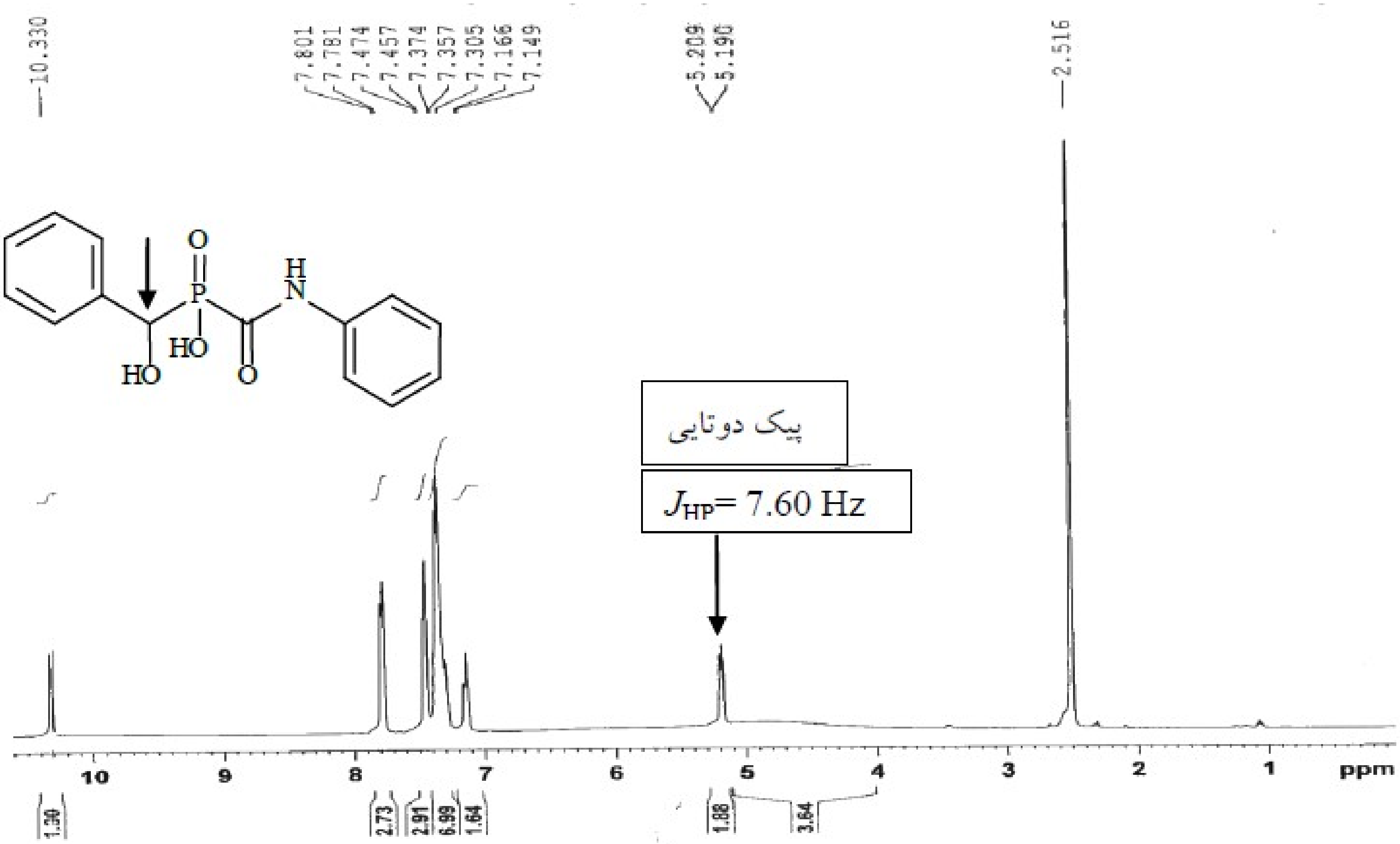

Figure S1: ^{1}H NMR Spectra of 3a

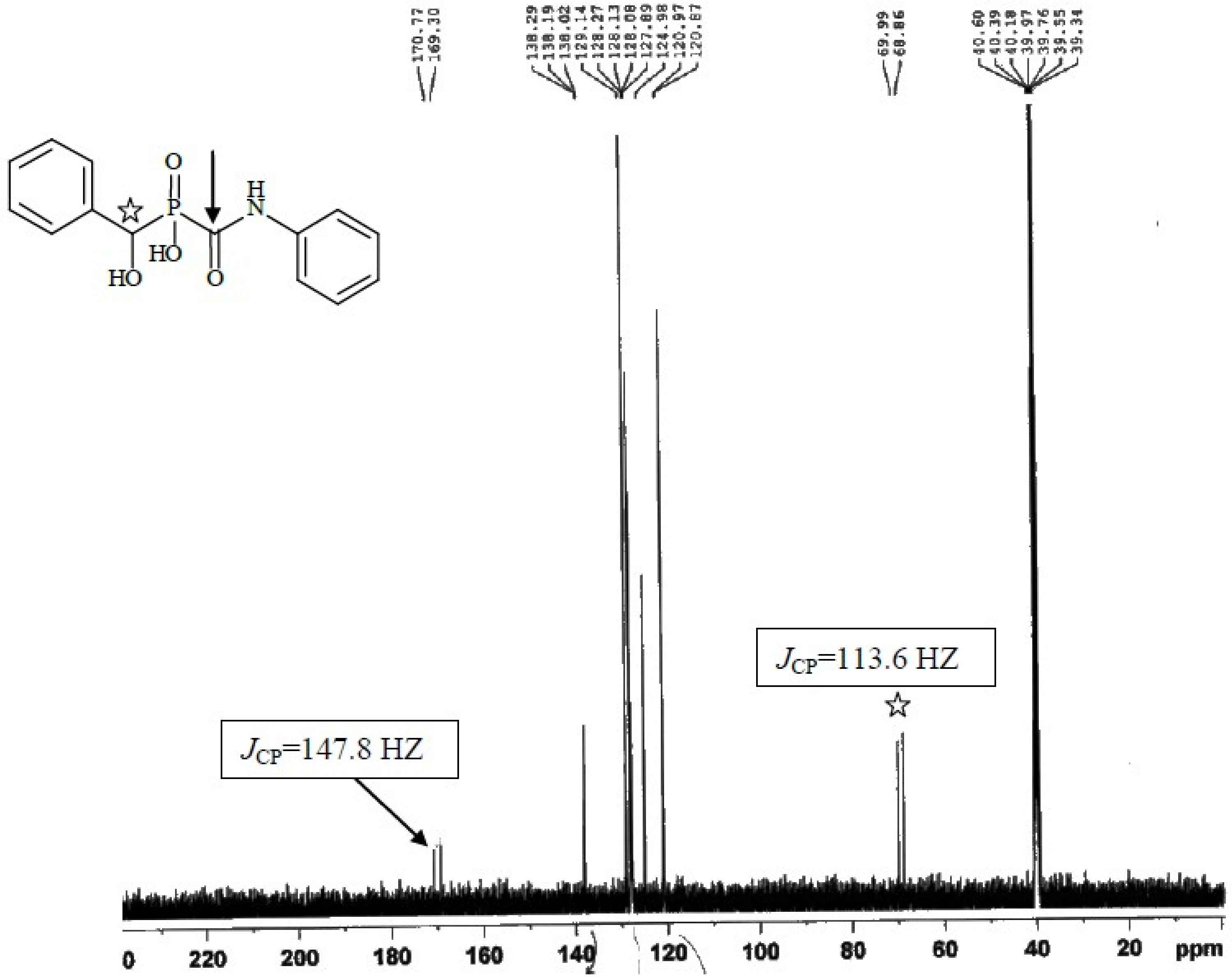

Figure S2: ^{13}C NMR Spectra of 3a

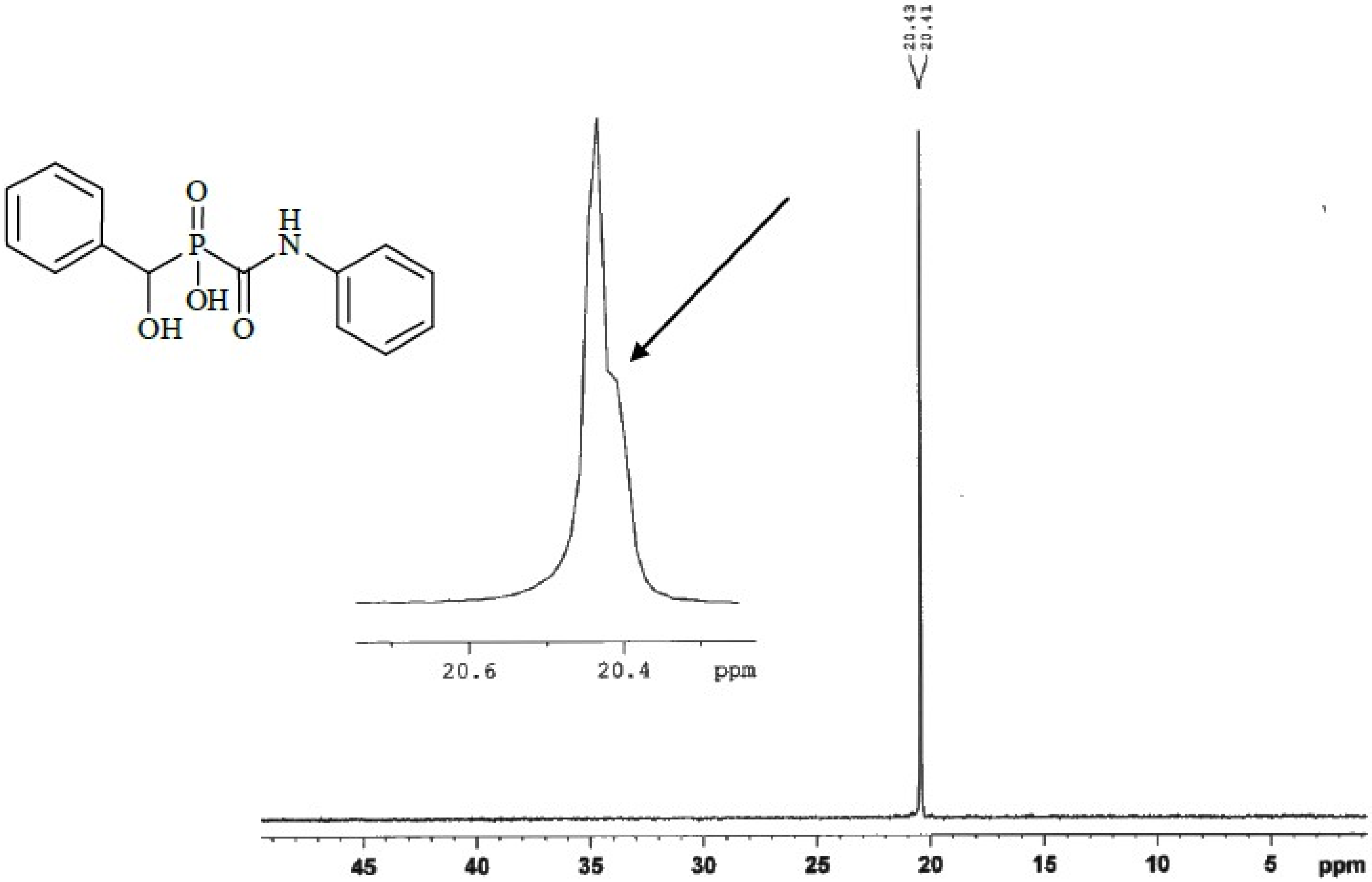

Figure S3: ^{31}P NMR Spectra of 3a

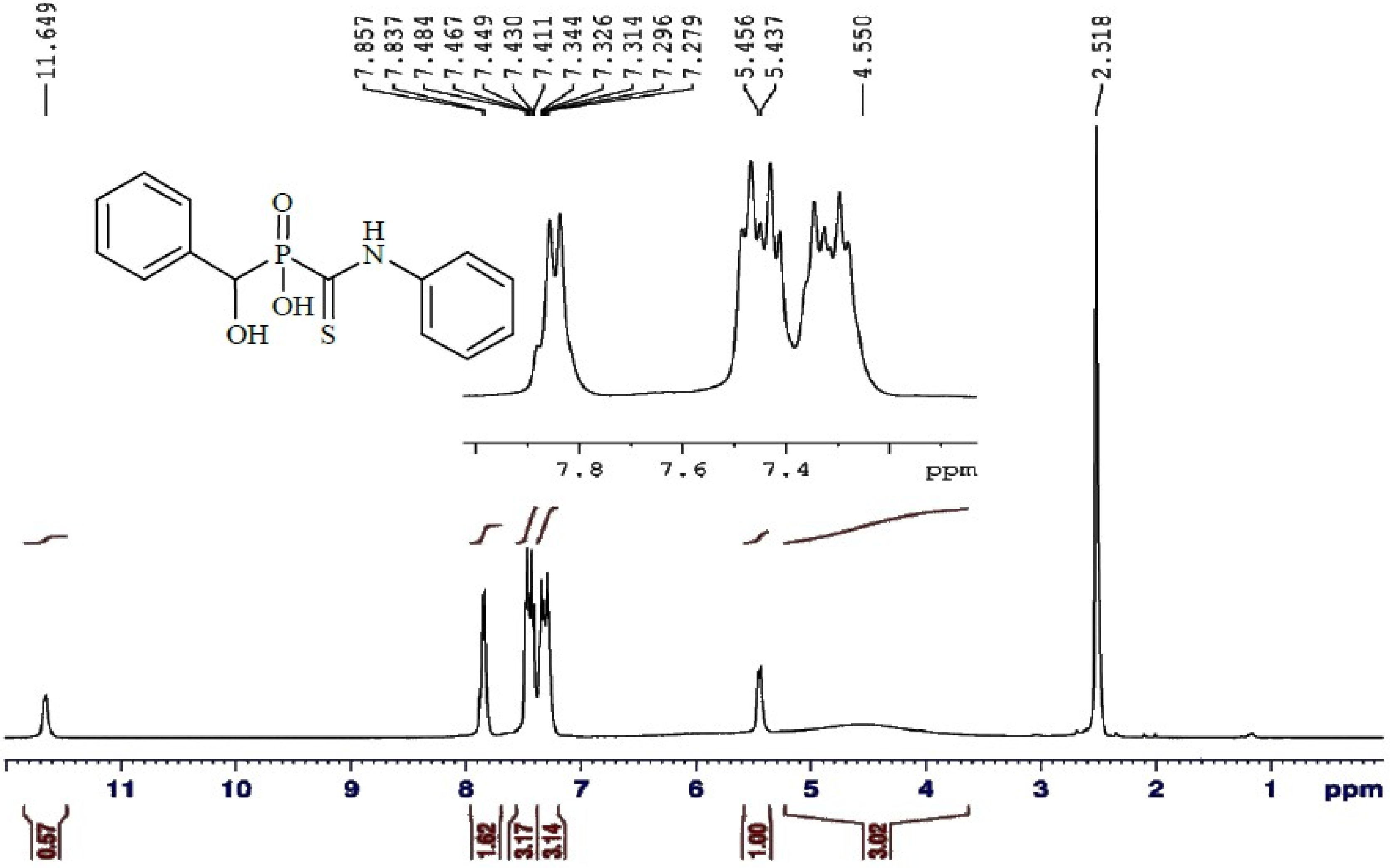

Figure S4: ^{1}H NMR Spectra of 3b

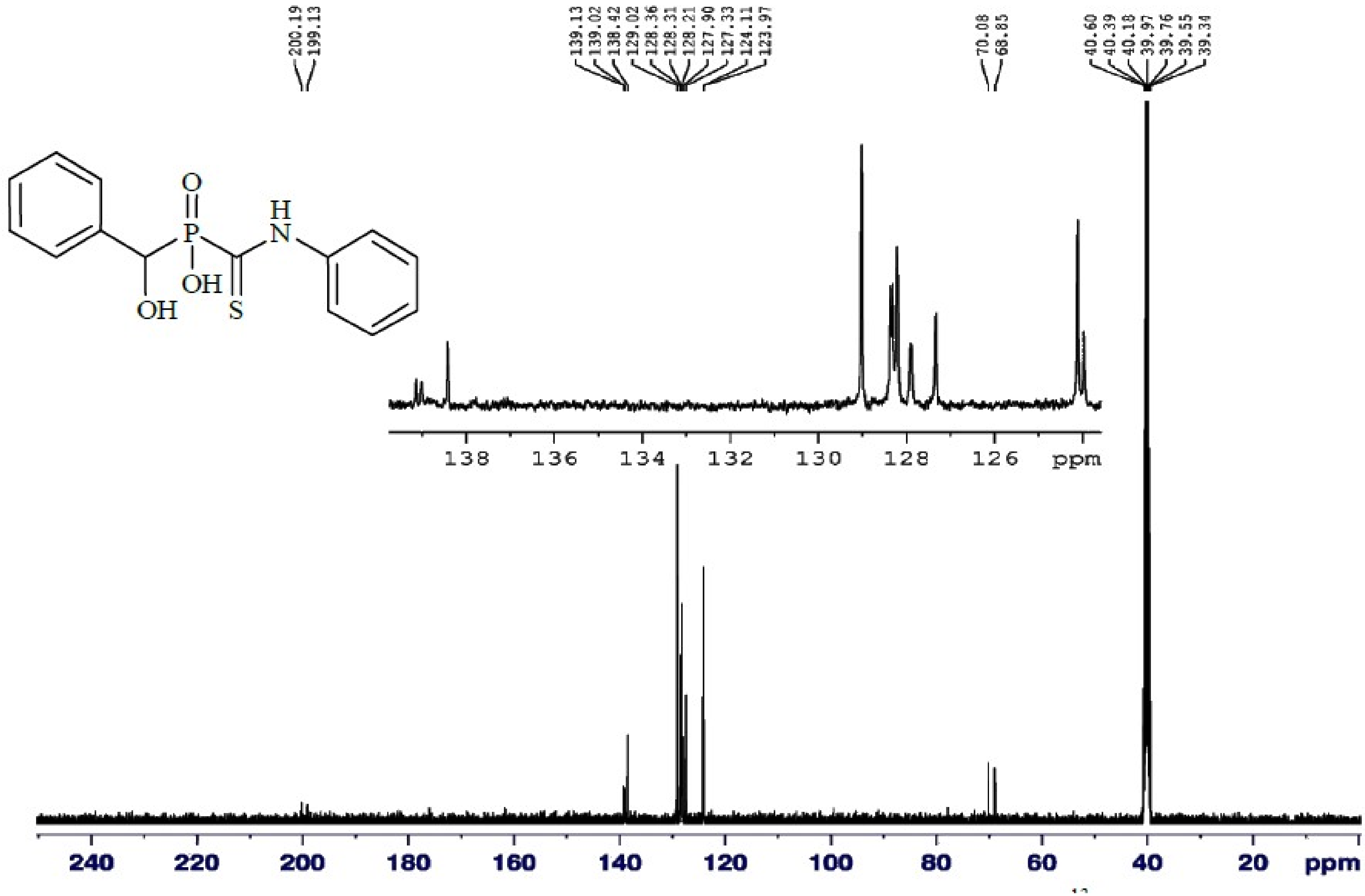

Figure S5: ^{13}C NMR Spectra of 3b

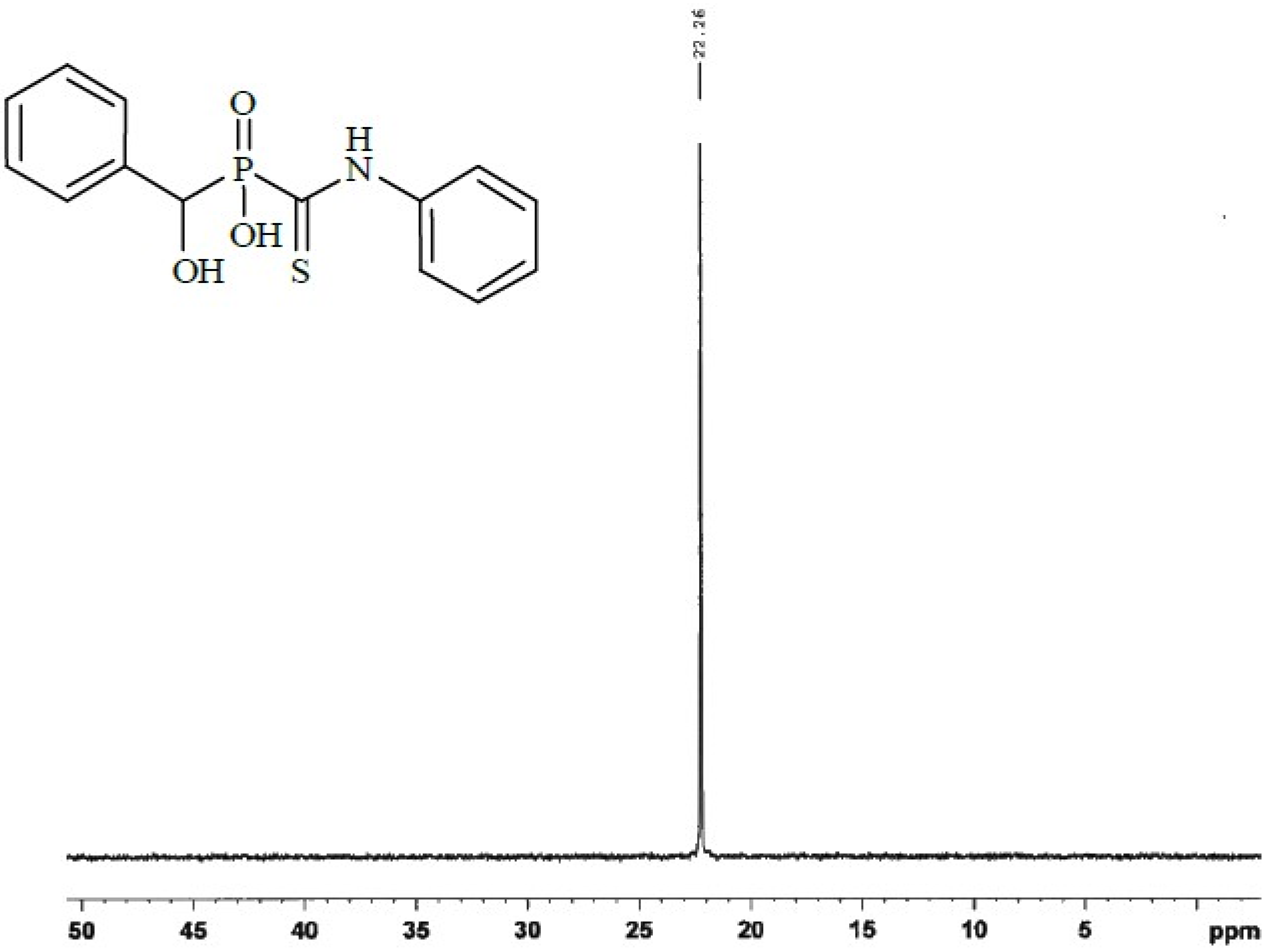

Figure S6: ^{31}P NMR Spectra of 3b

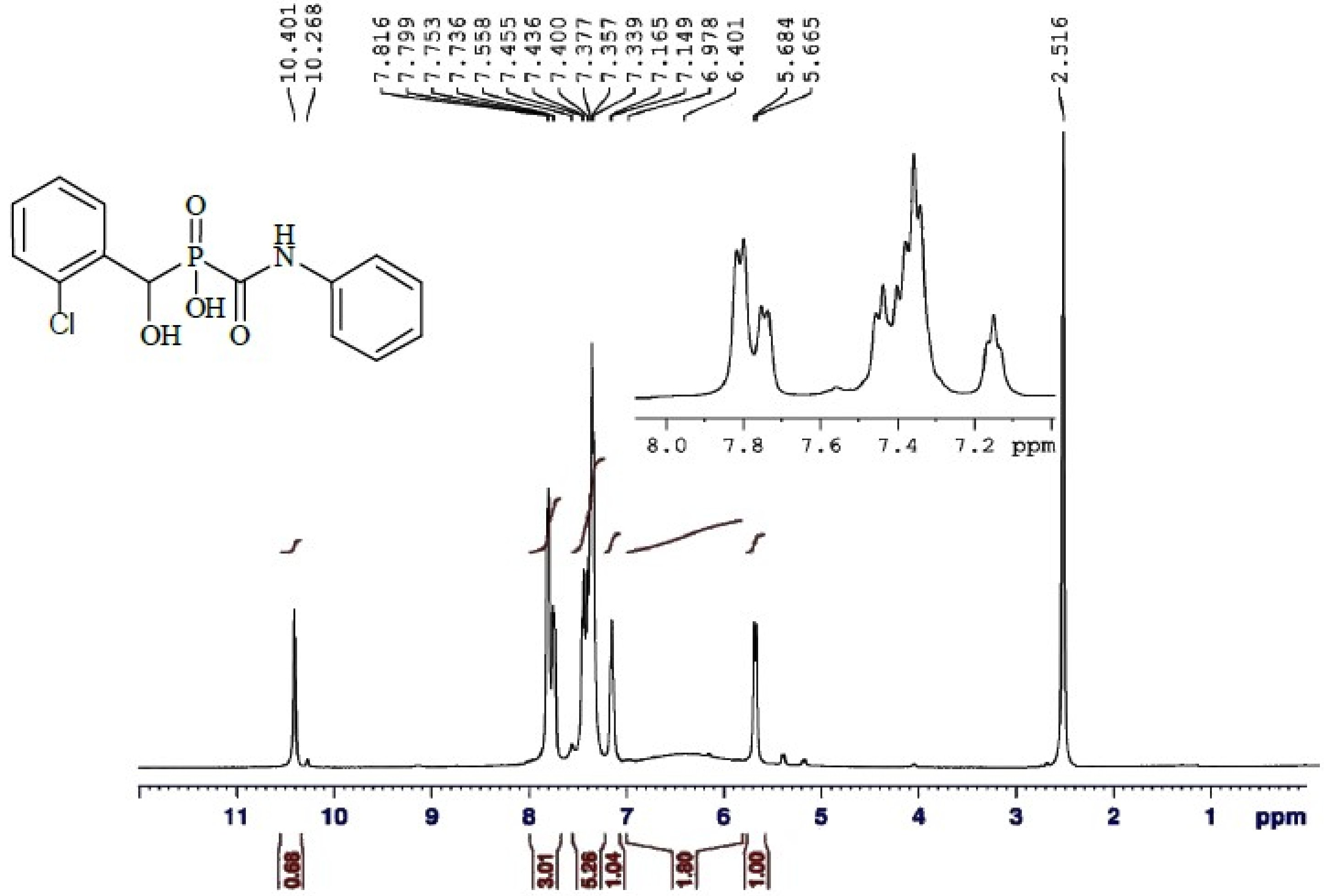

Figure S7: ^{1}H NMR Spectra of 3c

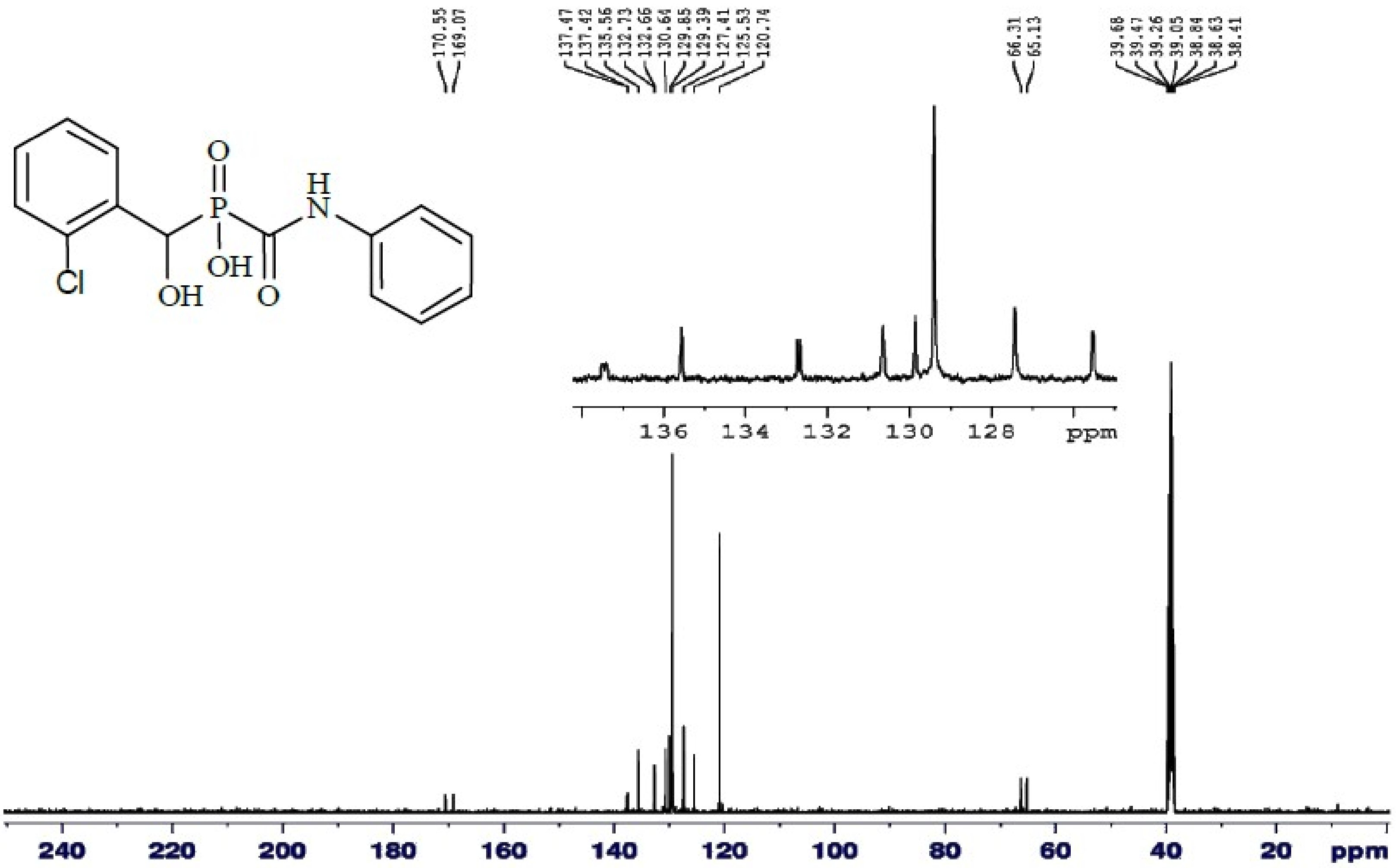

Figure S8: ^{13}C NMR Spectra of 3c

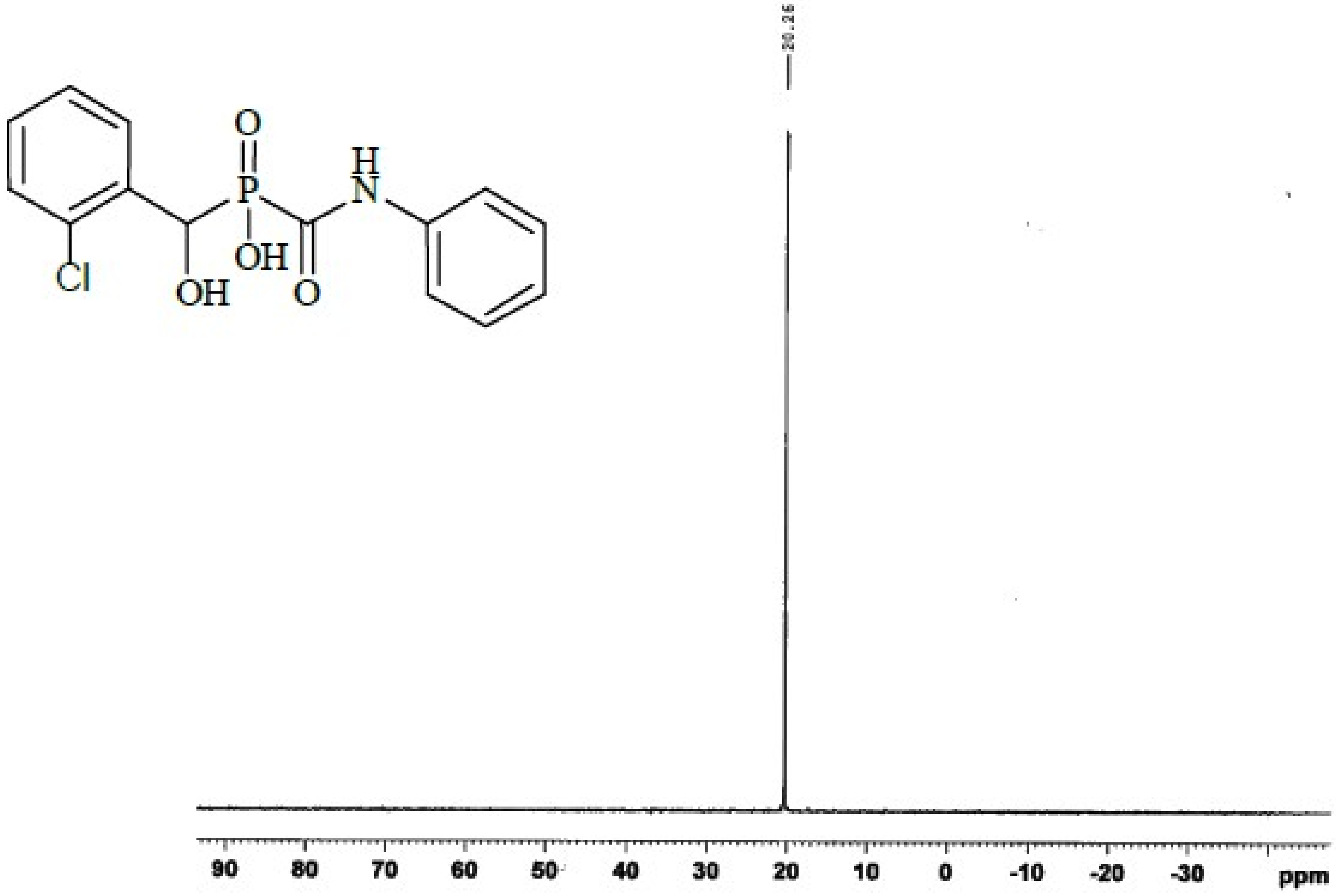

Figure S9: ^{31}P NMR Spectra of 3c

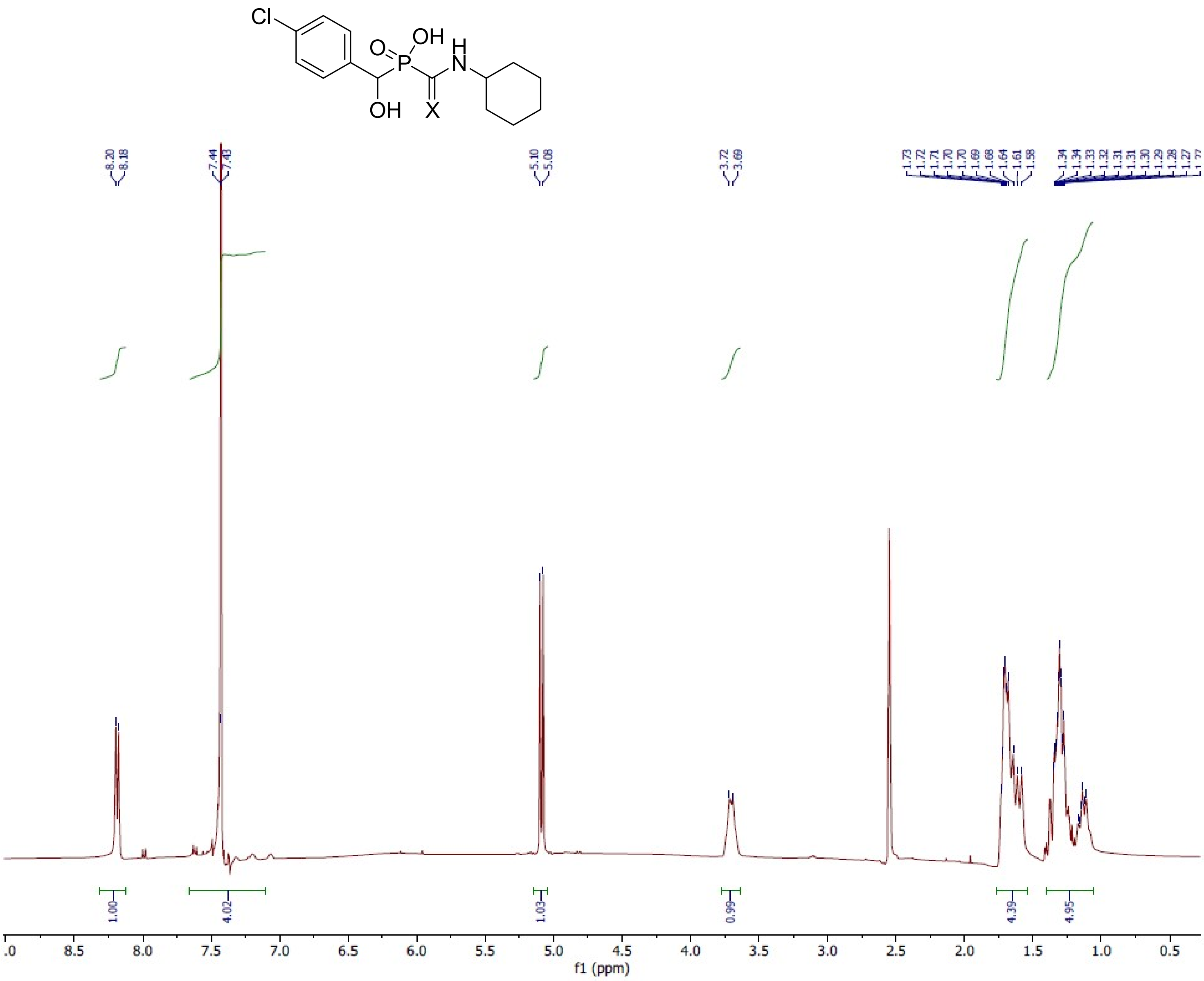

Figure S10: ^{1}H NMR Spectra of 3d

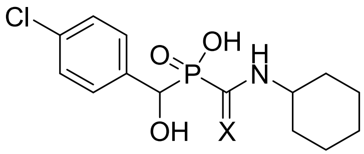

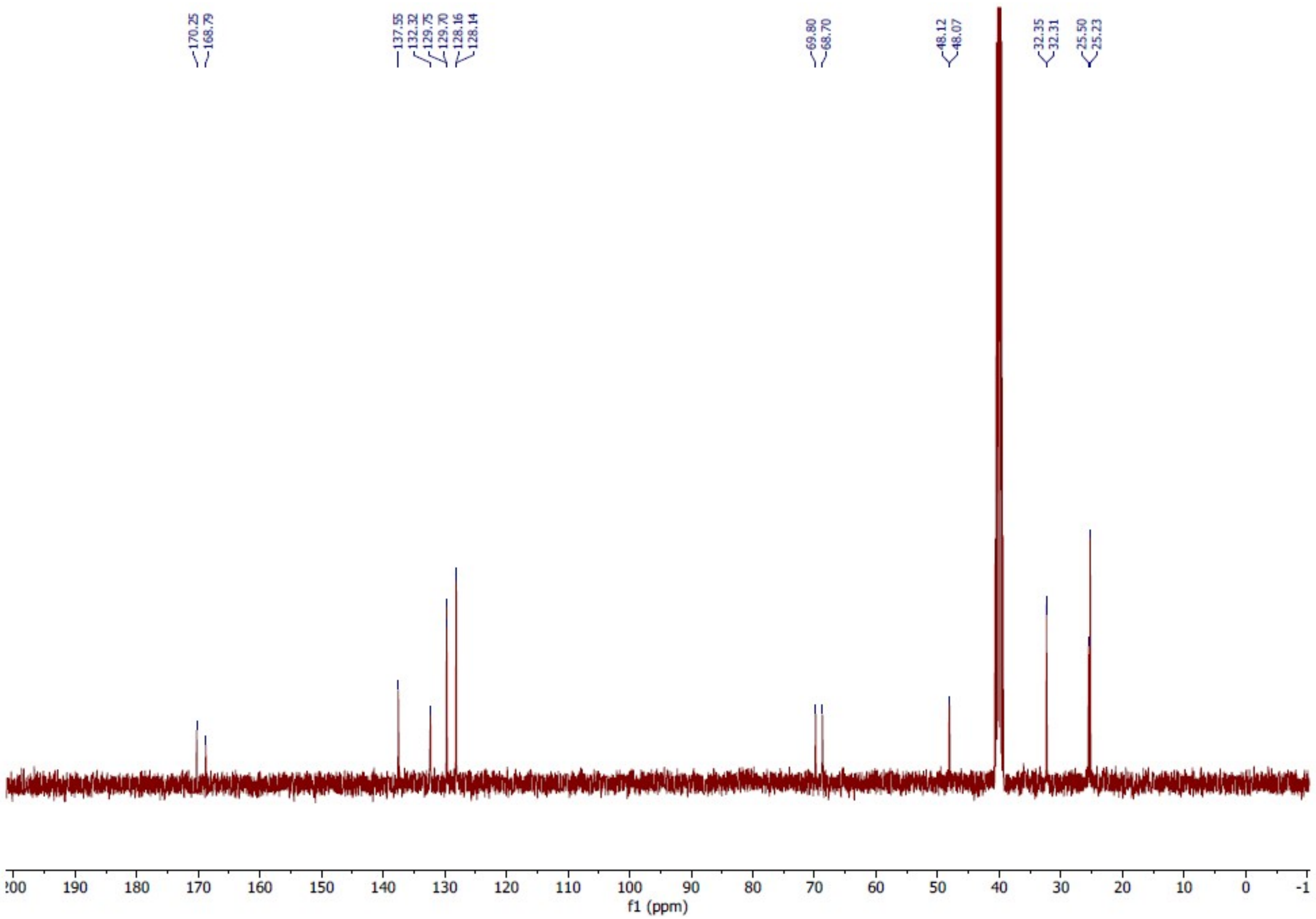

Figure S11: ^{13}C NMR Spectra of 3d

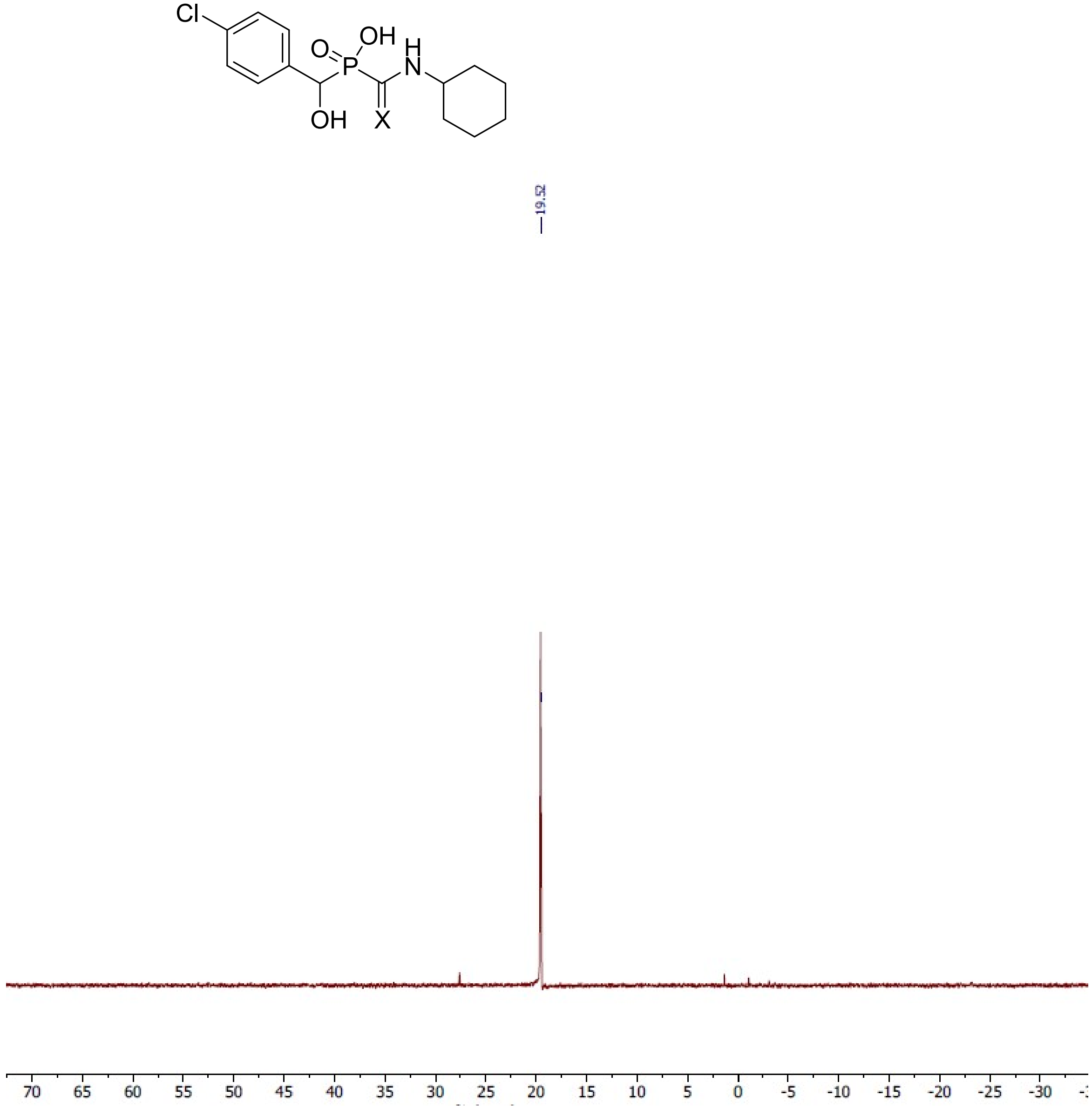

Figure S12: ^{31}P NMR Spectra of 3d

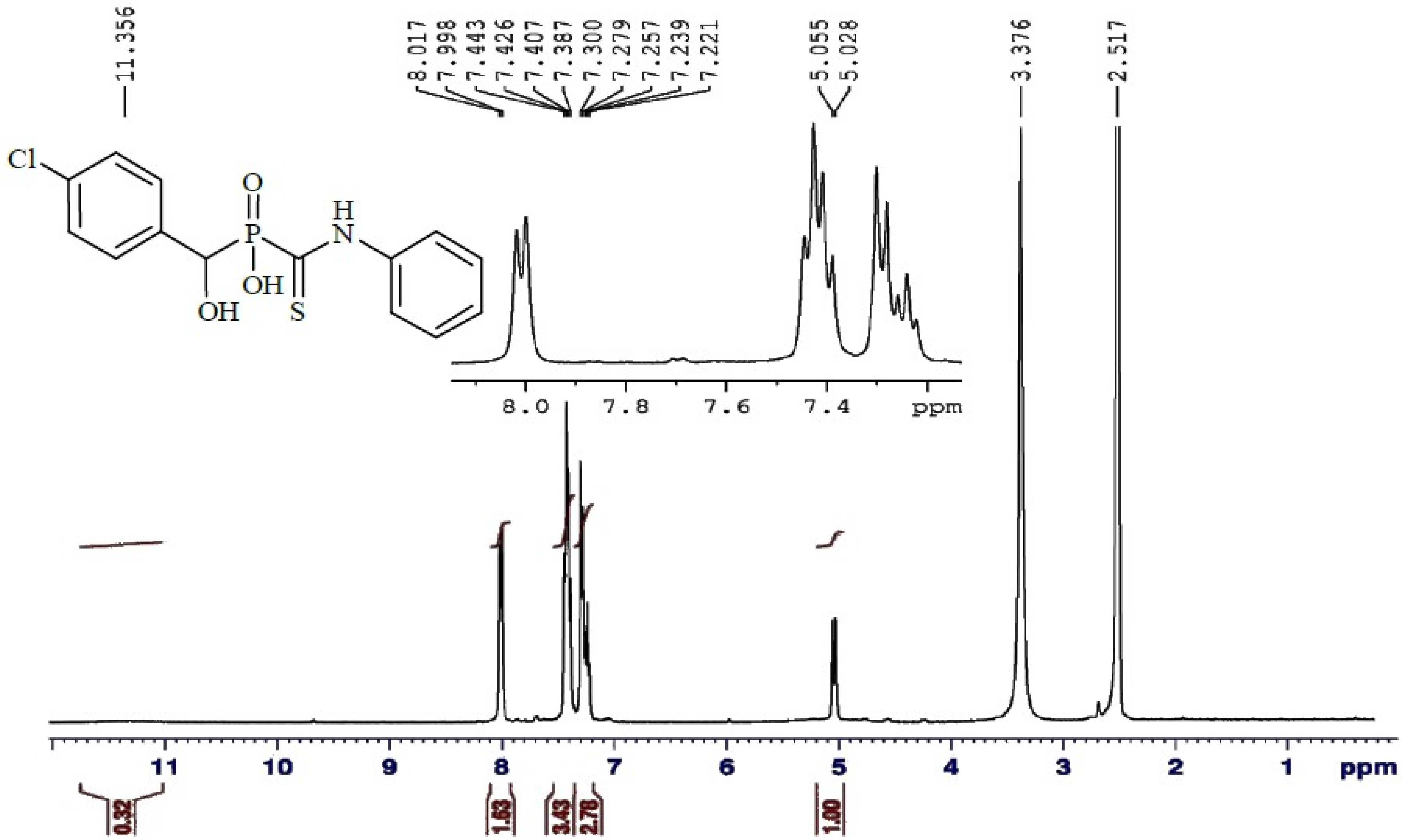
11.356
8.017
7.998
7.443
7.426
7.407
7.387
7.300
7.279
7.257
7.239
7.221
5.055
5.028
3.376
2.517
Cl
O
P
OH
OH
H
N
S
8.0
7.8
7.6
7.4
ppm
11
10
9
8
7
6
5
4
3
2
1
ppm
0.32
1.63
3.43
2.76
1.00

Figure S13: ^{1}H NMR Spectra of 3e

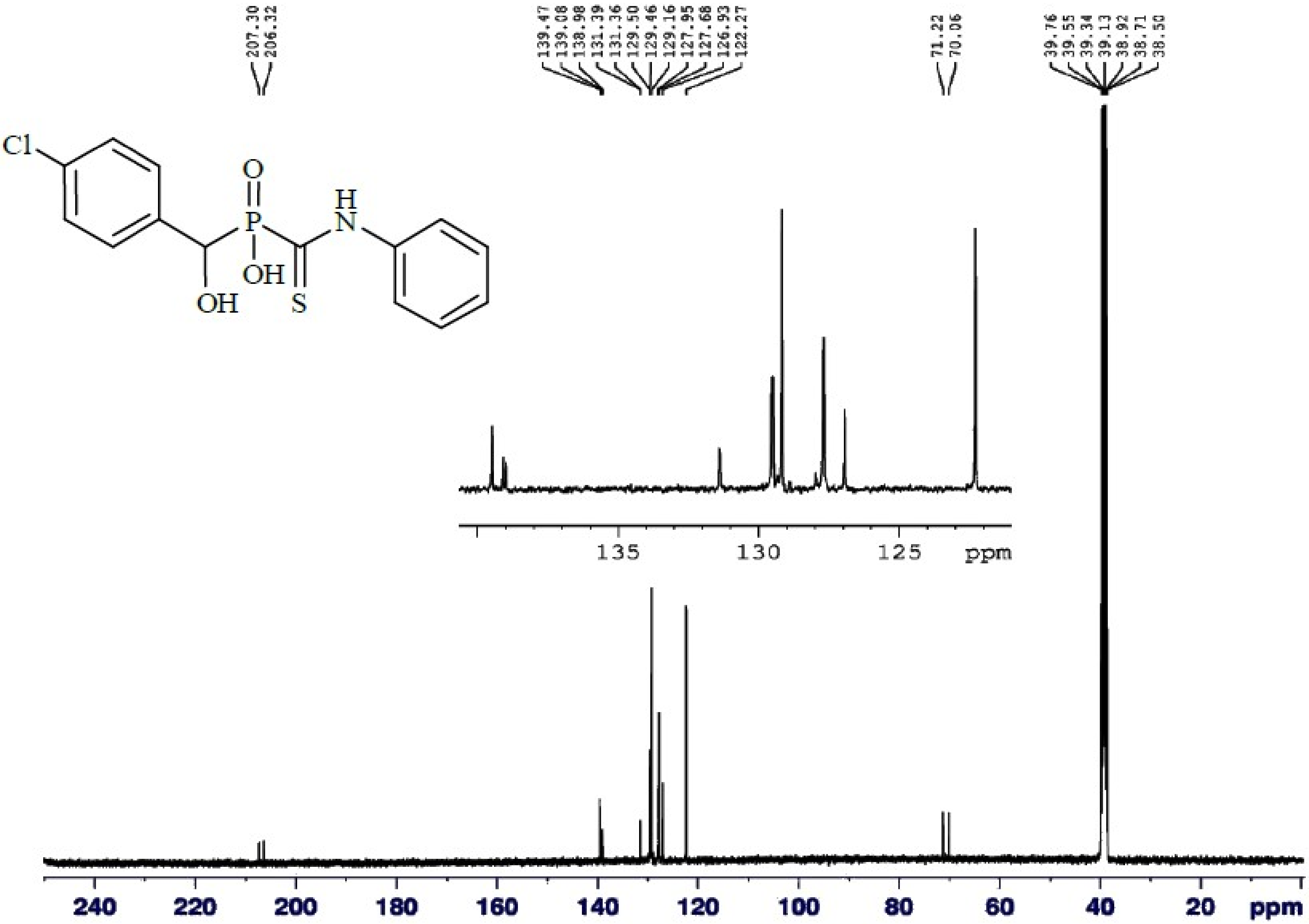

Figure S14: ^{13}C NMR Spectra of 3e

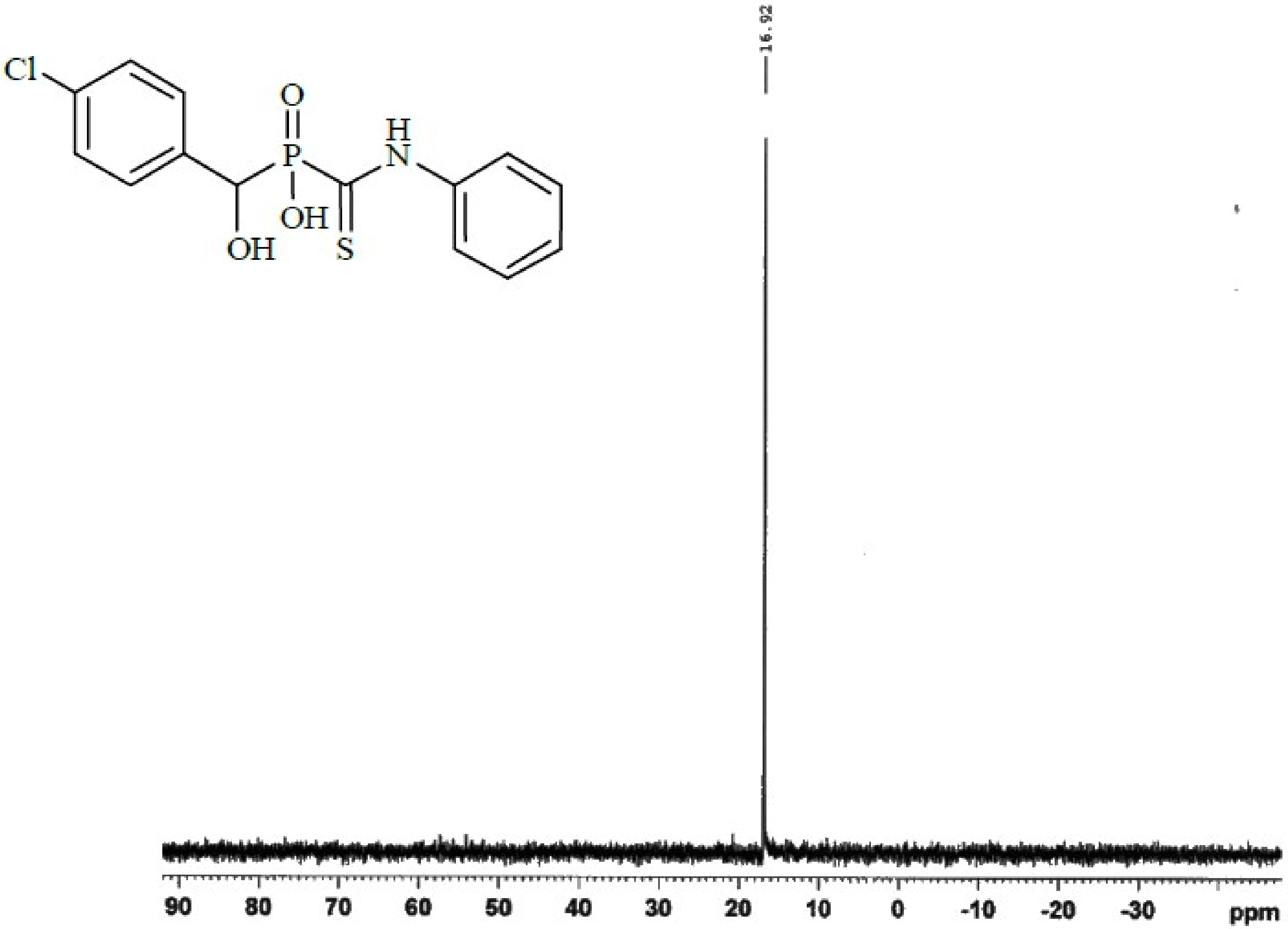

Figure S15: ^{31}P NMR Spectra of 3e

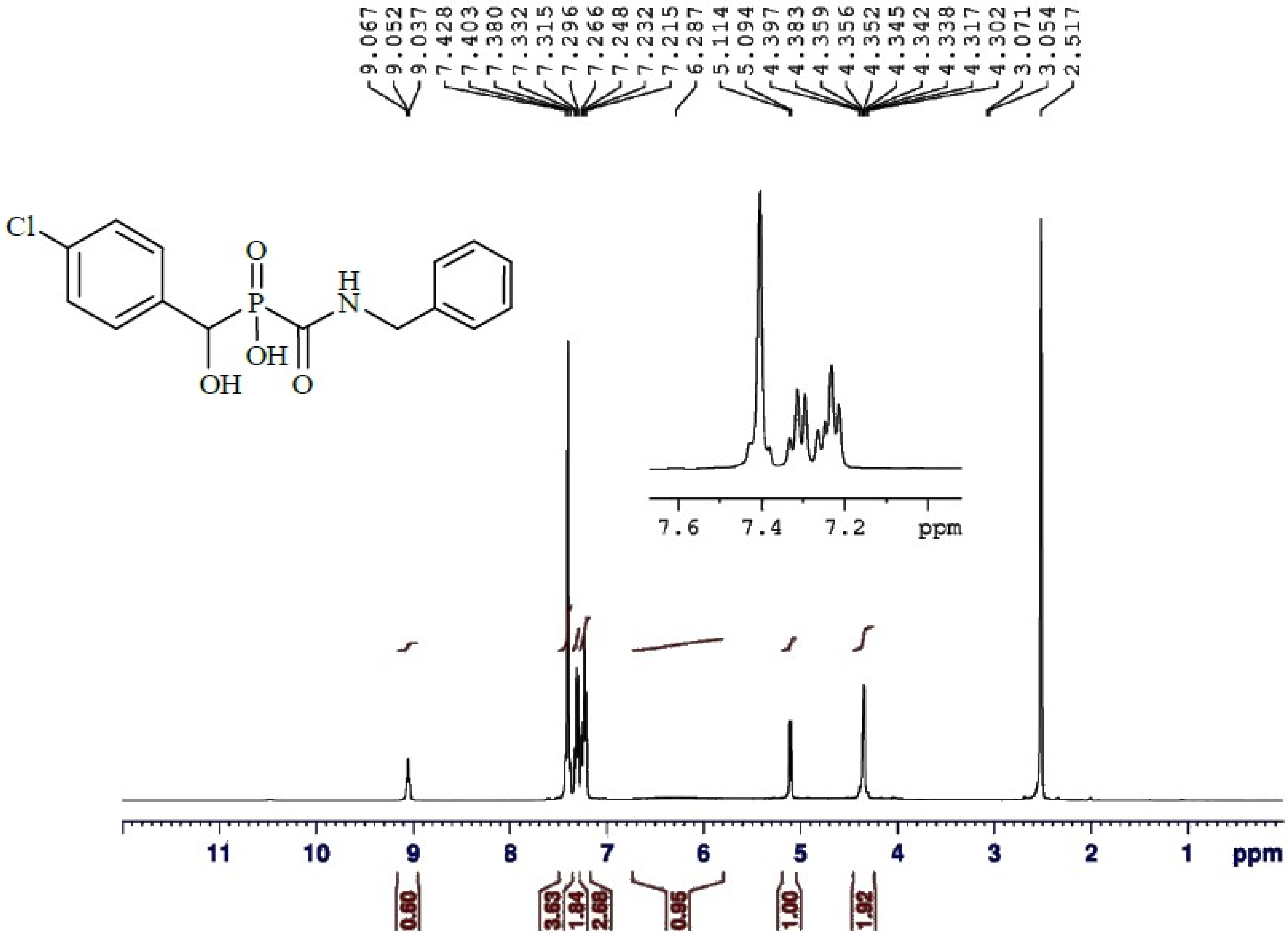

Figure S16: ^{1}H NMR Spectra of 3f

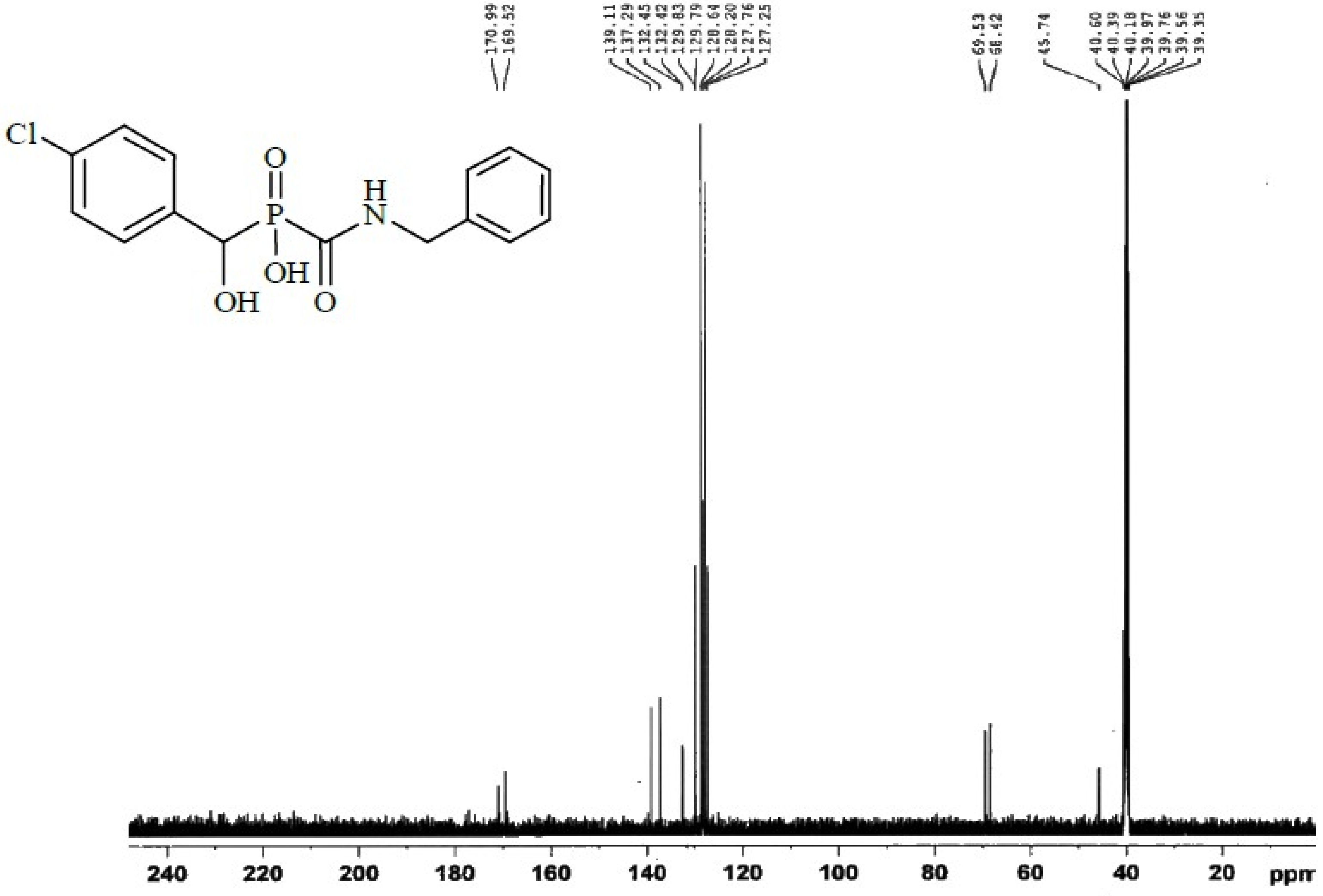

Figure S17: ^{13}C NMR Spectra of 3f

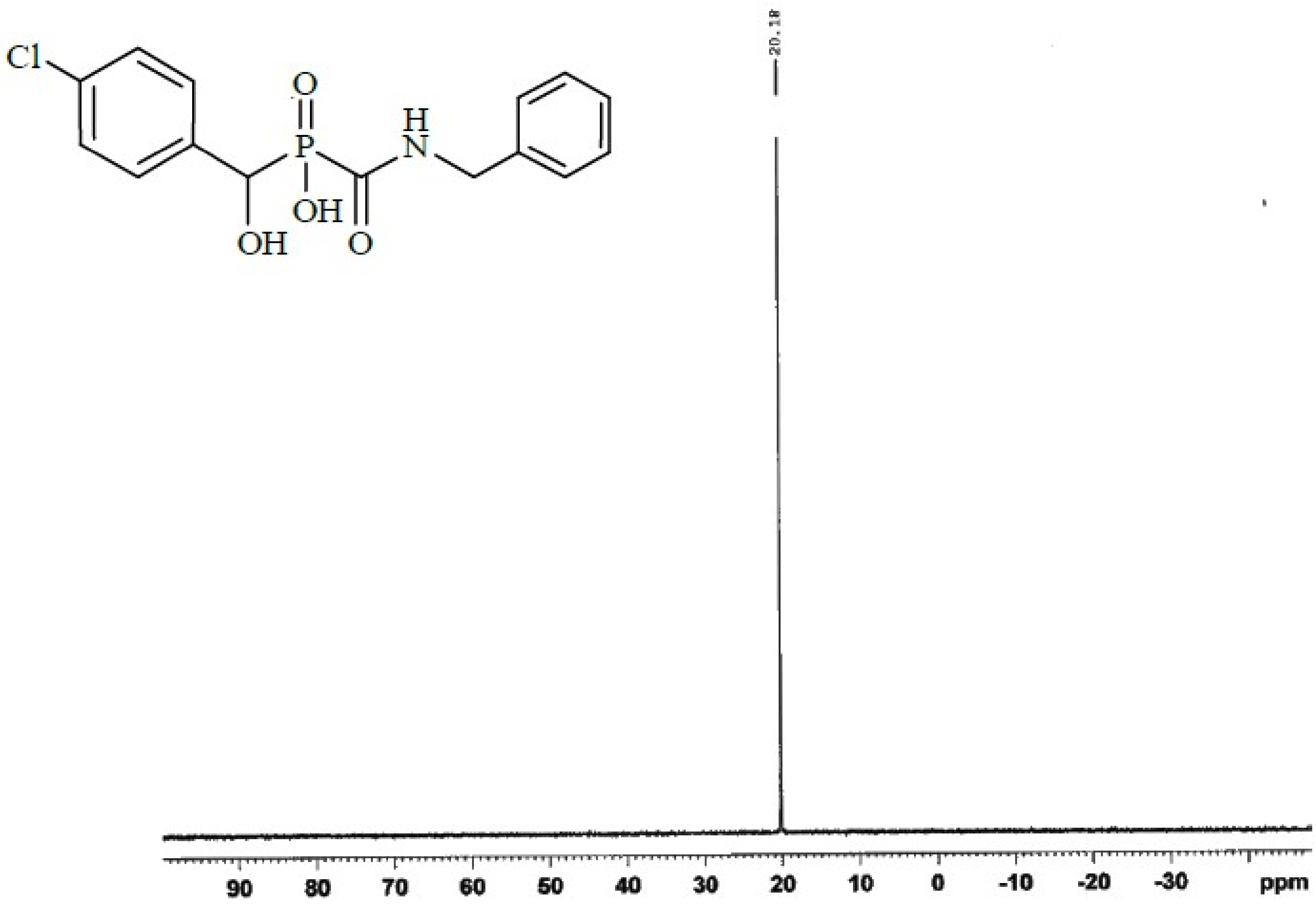

Figure S18: ^{31}P NMR Spectra of 3f

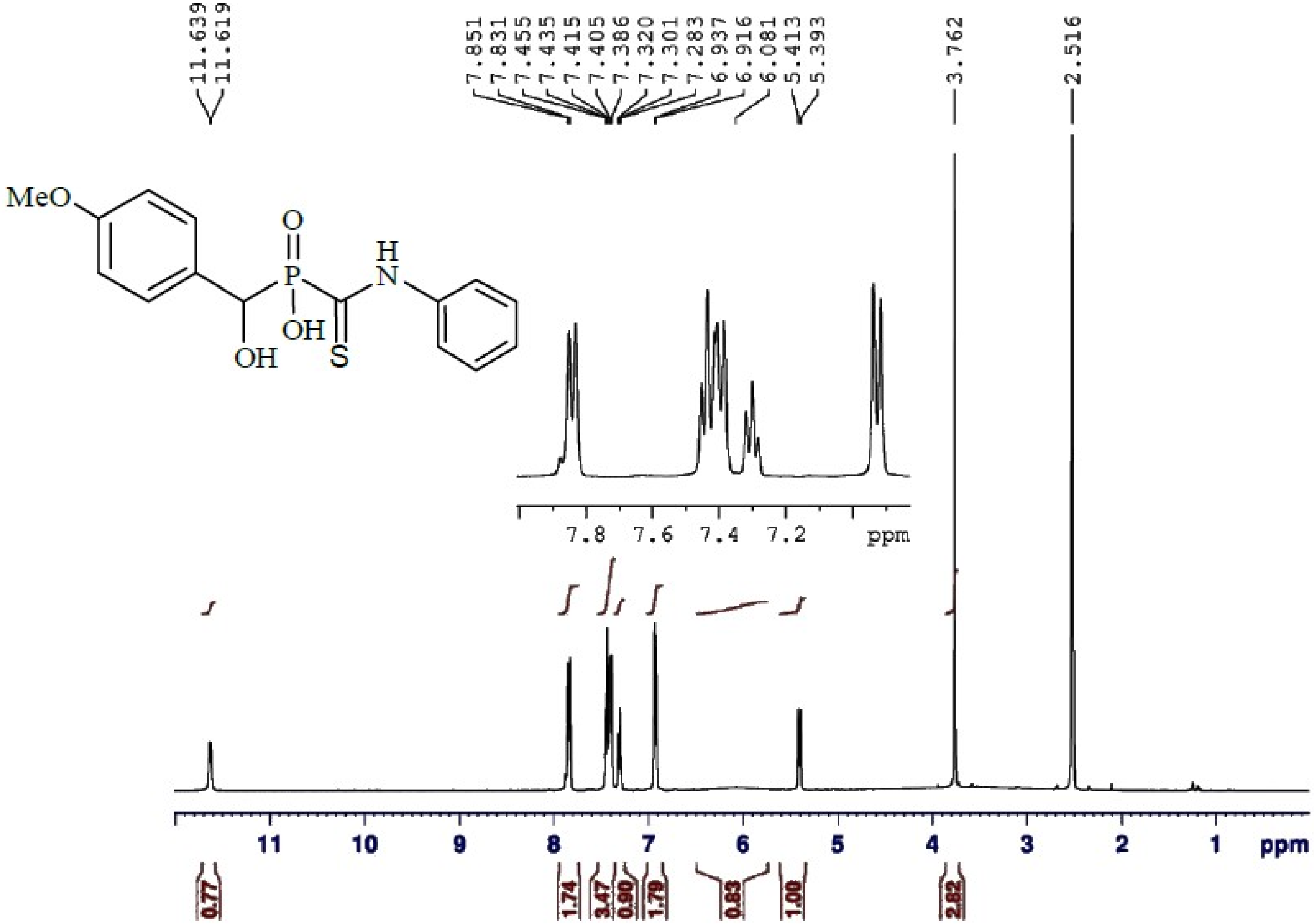

Figure S19: ^{1}H NMR Spectra of 3g

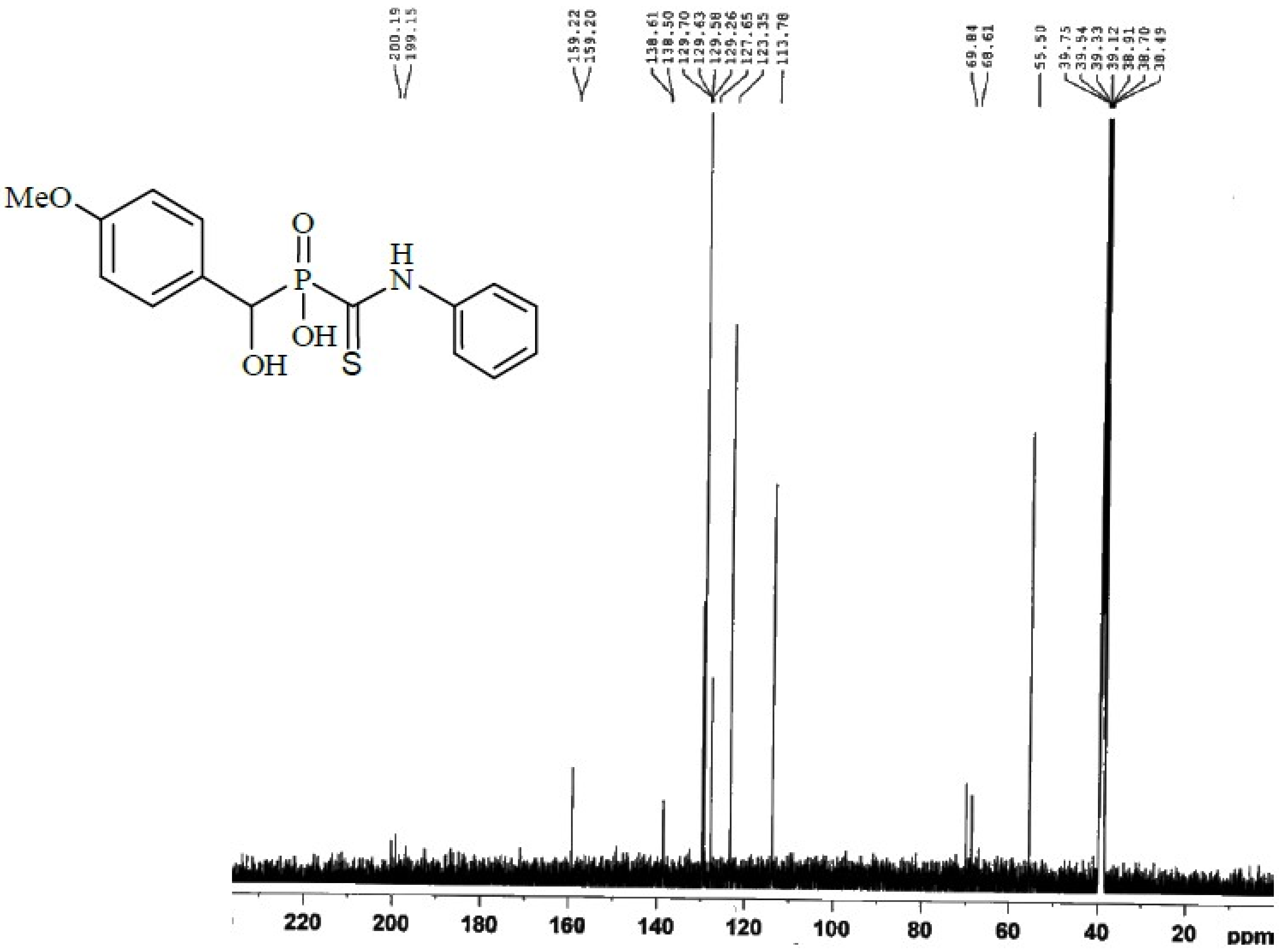

Figure S20: ^{13}C NMR Spectra of 3g

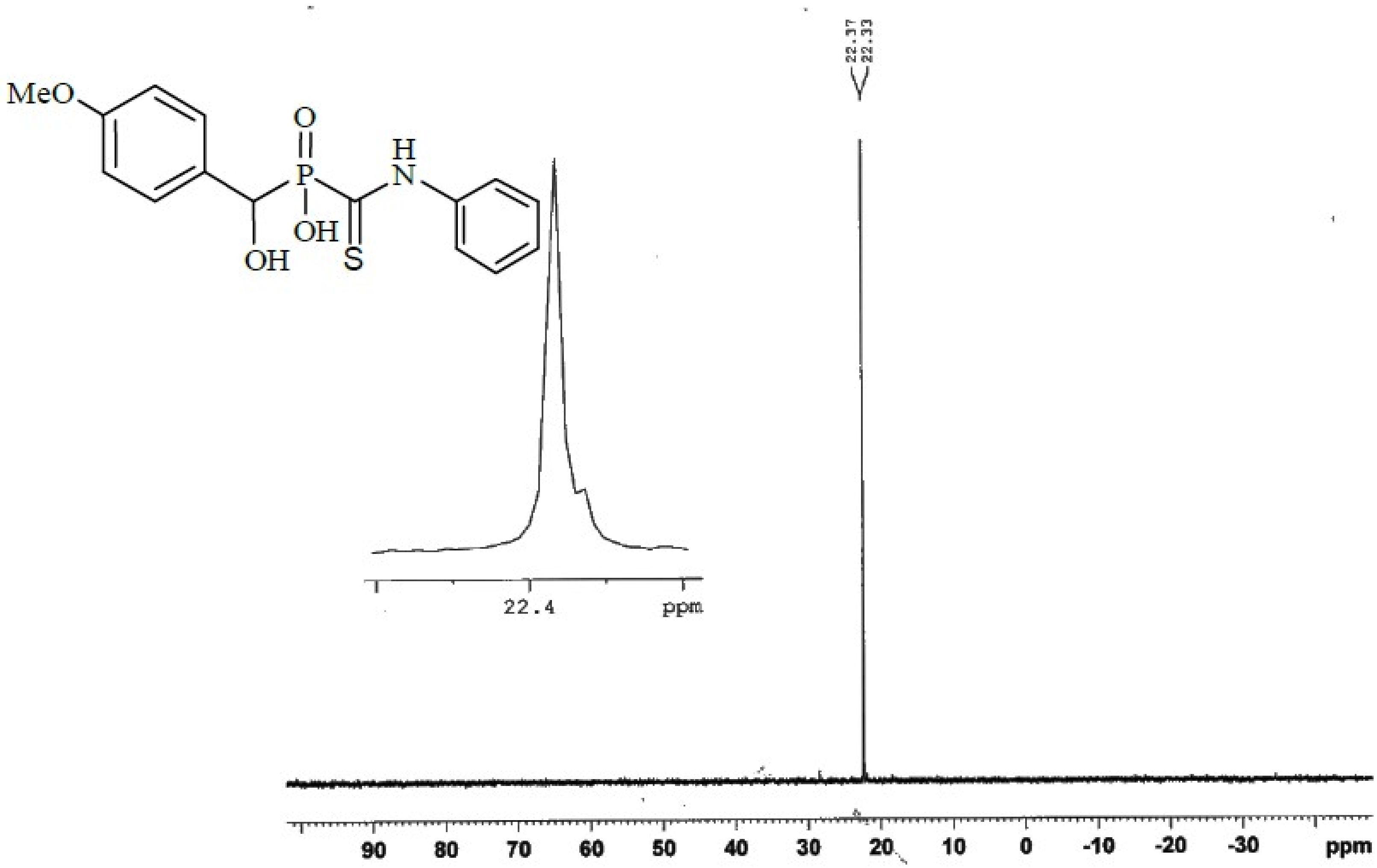

Figure S21: ^{31}P NMR Spectra of 3g

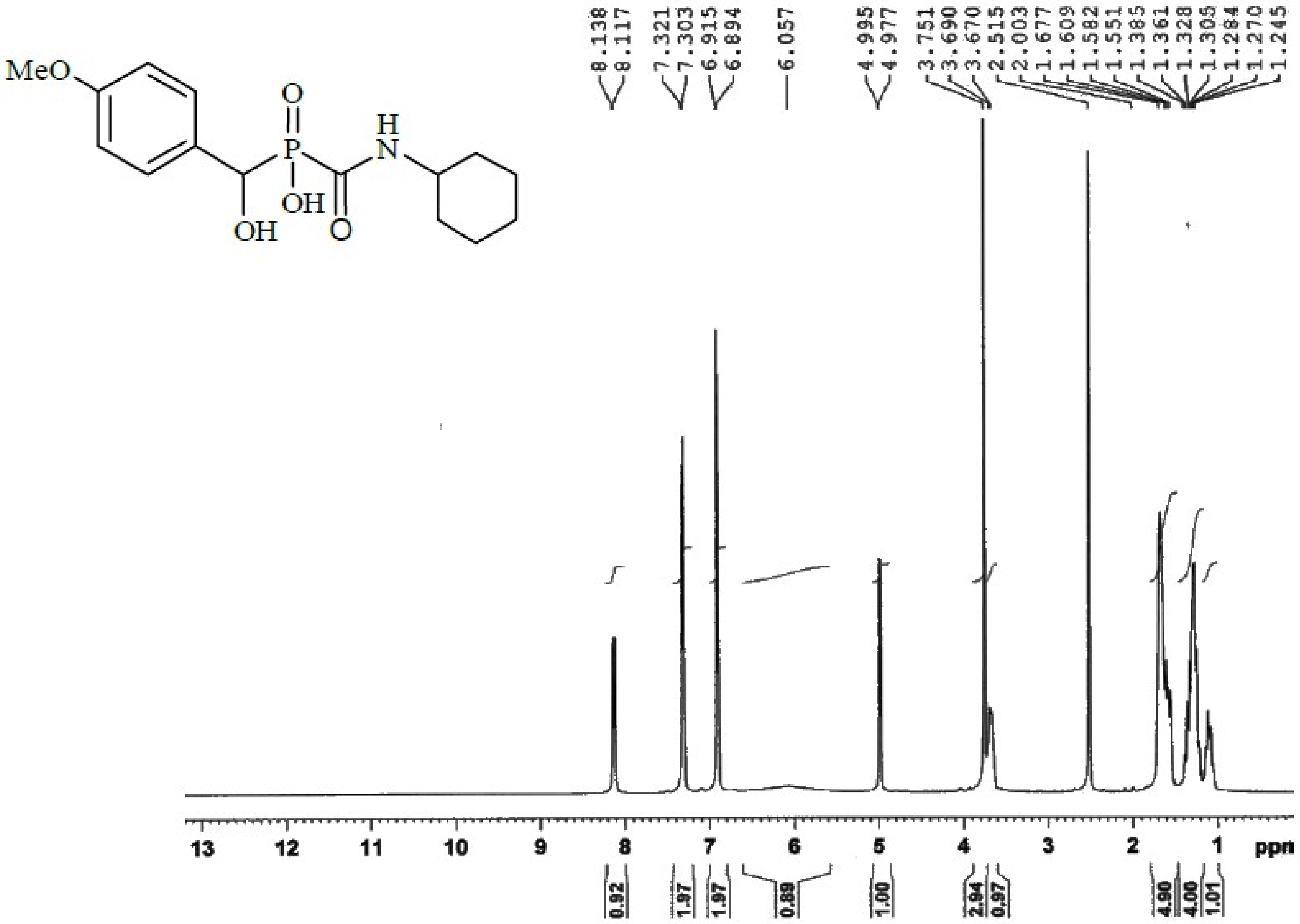

Figure S22: ^{1}H NMR Spectra of 3h

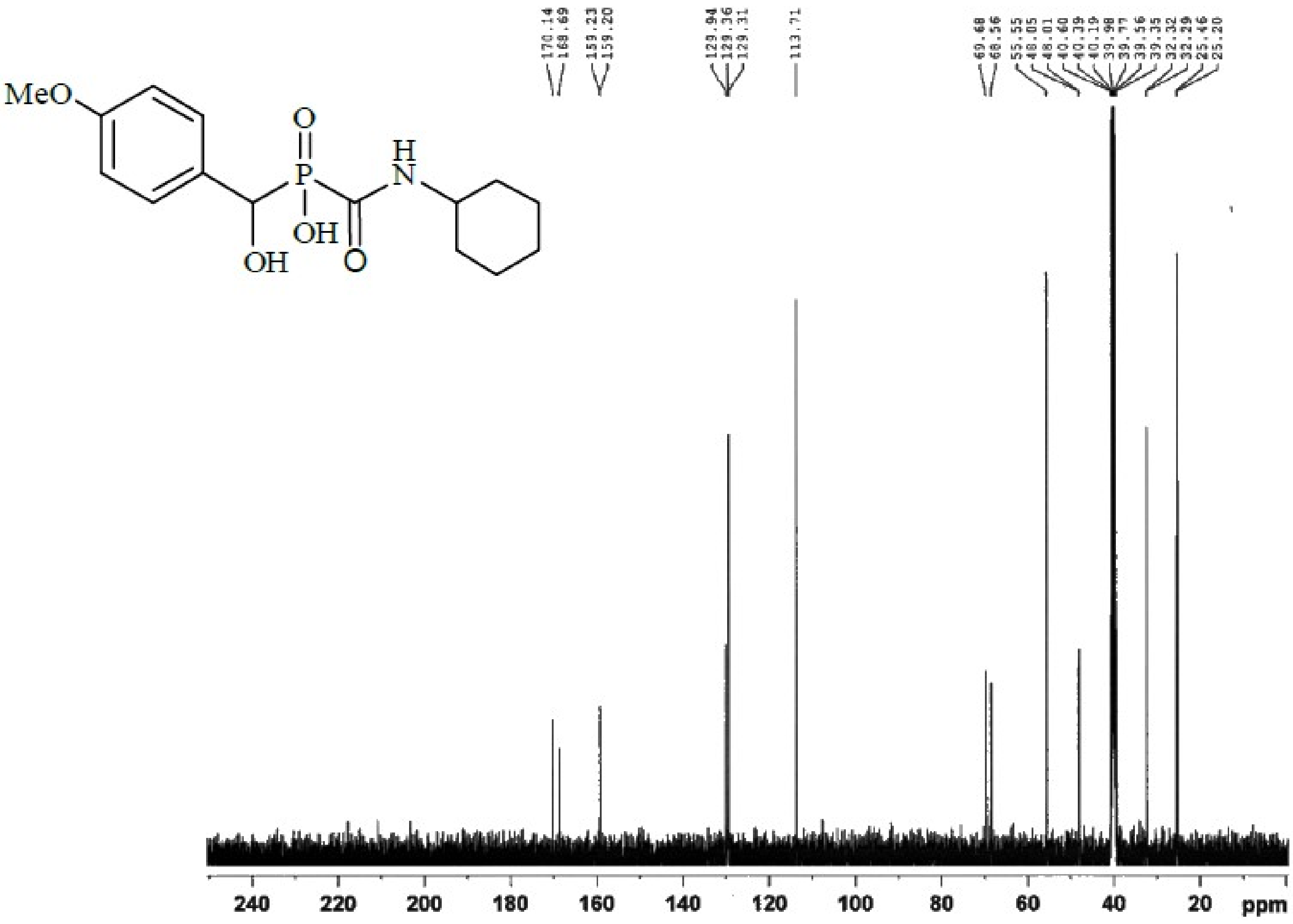

Figure S23: ^{13}C NMR Spectra of 3h

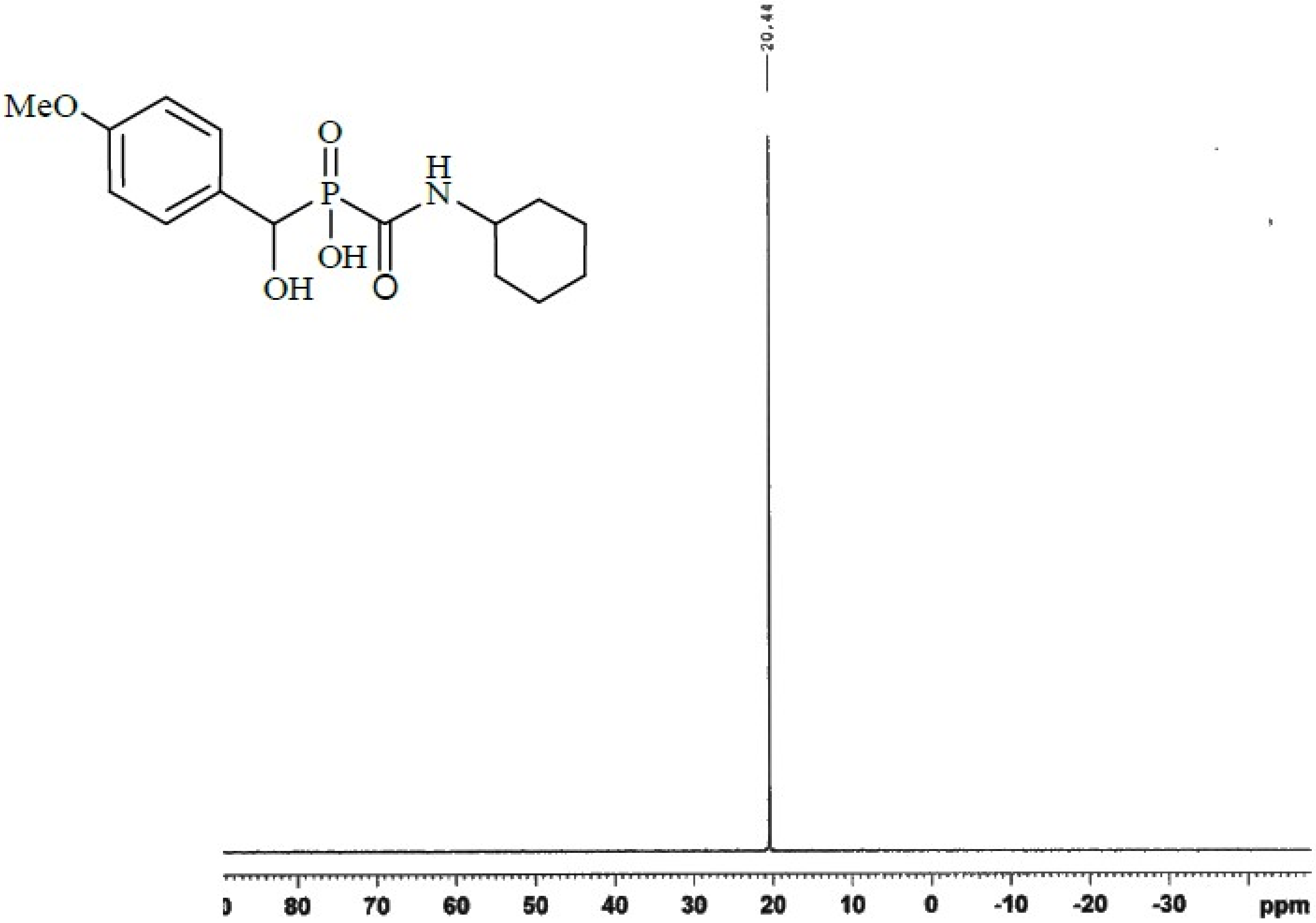

Figure S24: ^{31}P NMR Spectra of 3h

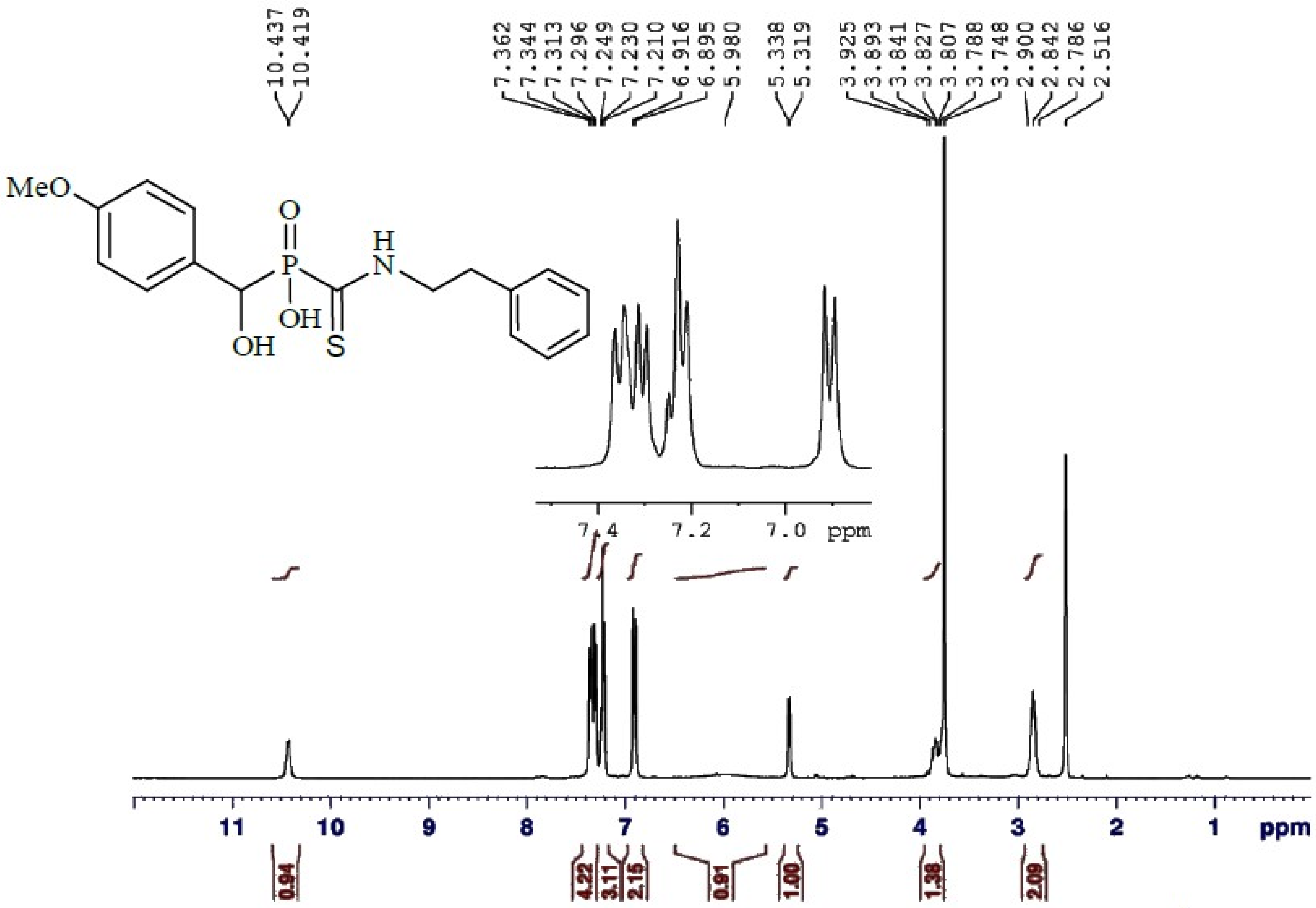

Figure S25: ^{1}H NMR Spectra of 3i

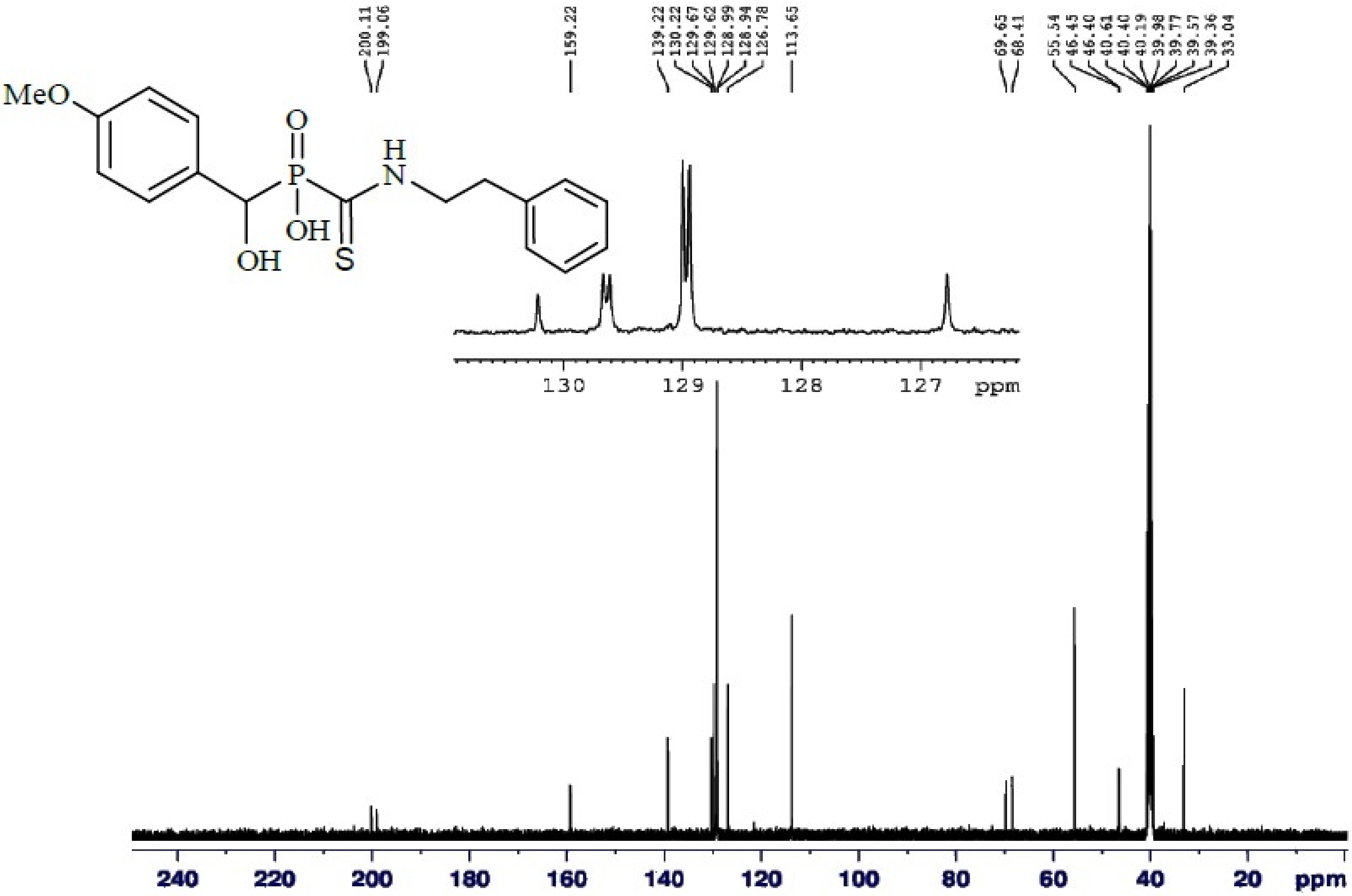

Figure S26: ^{13}C NMR Spectra of 3i

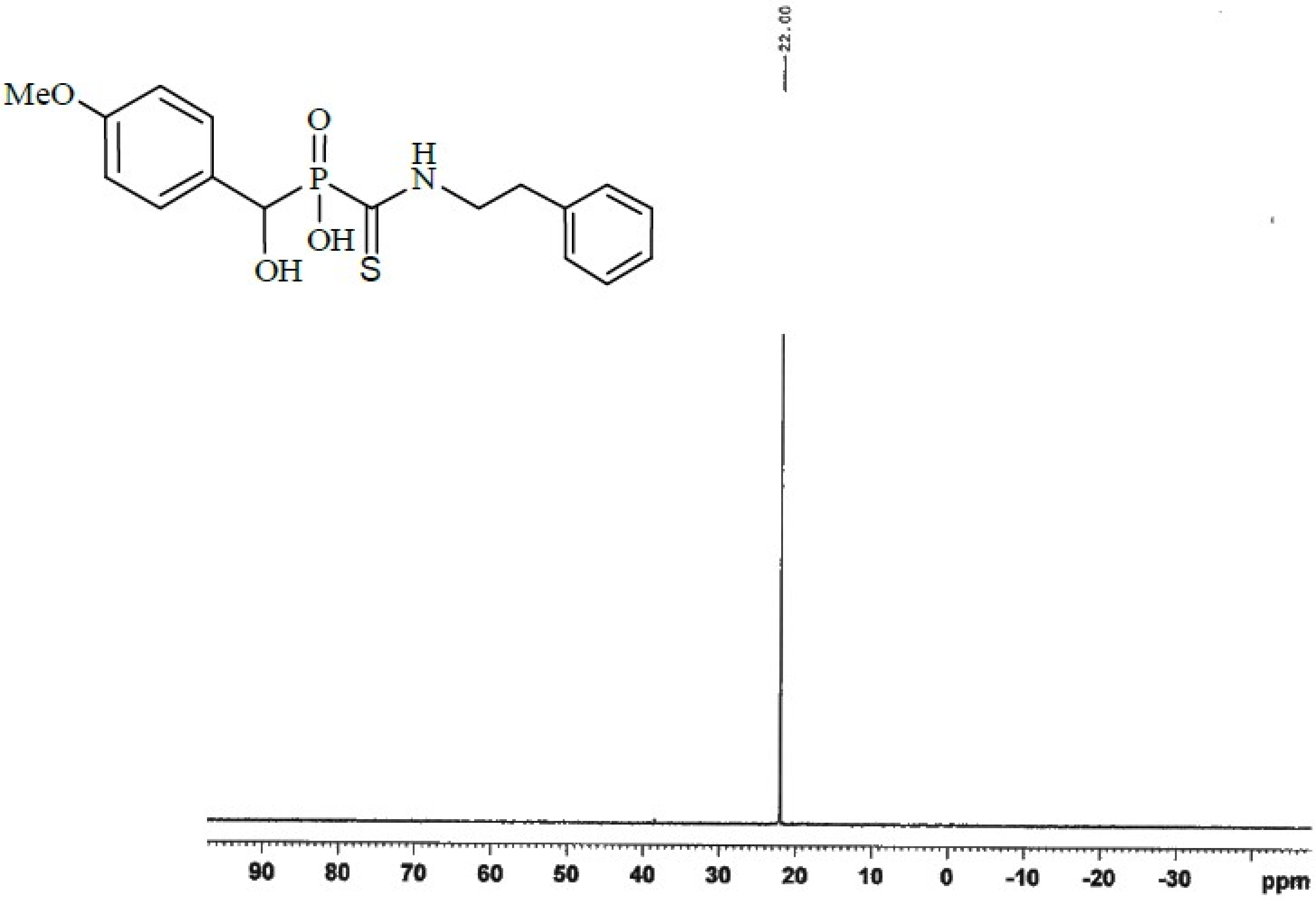

Figure S27: ^{31}P NMR Spectra of 3i

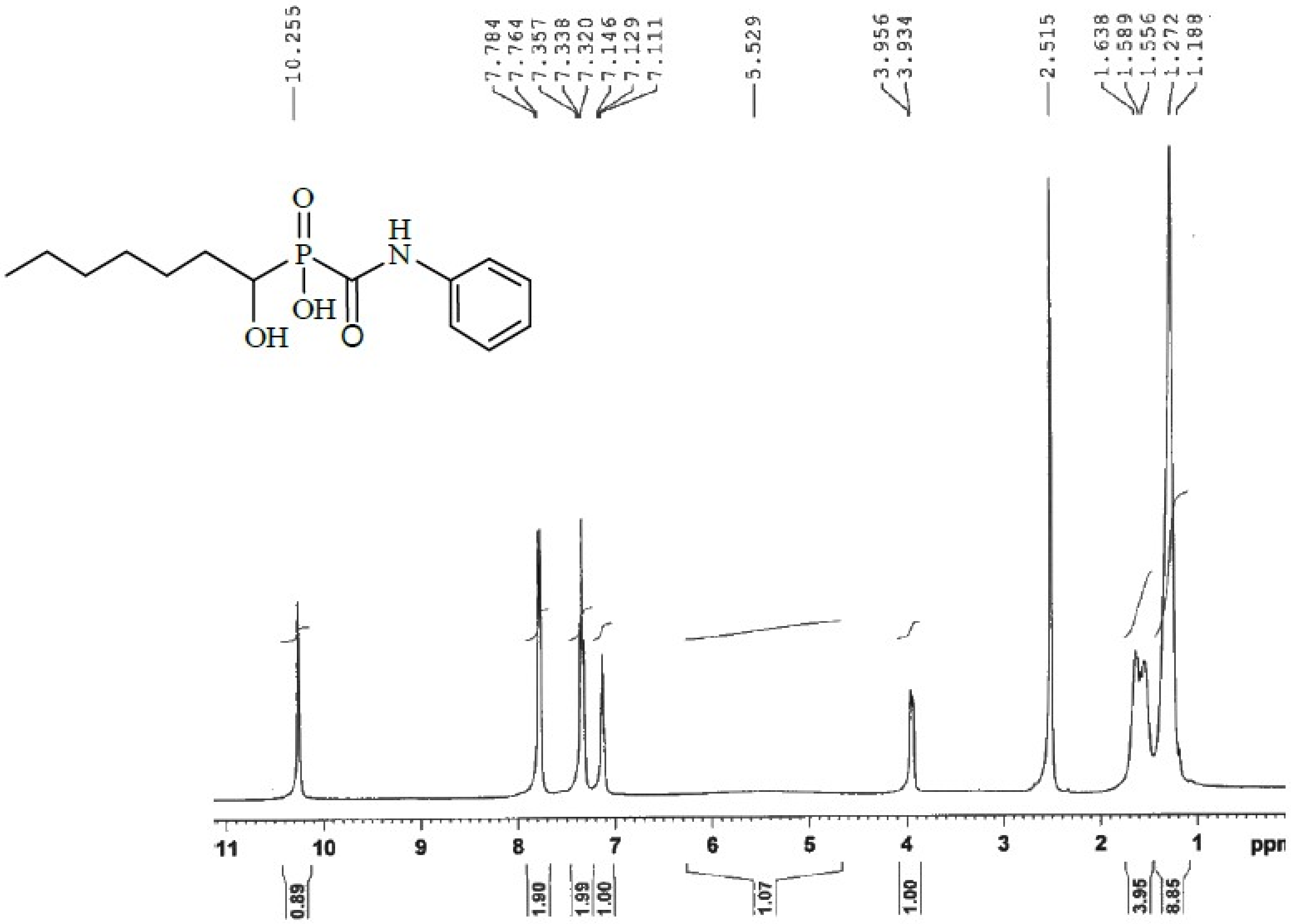

Figure S28: ^{1}H NMR Spectra of 3j

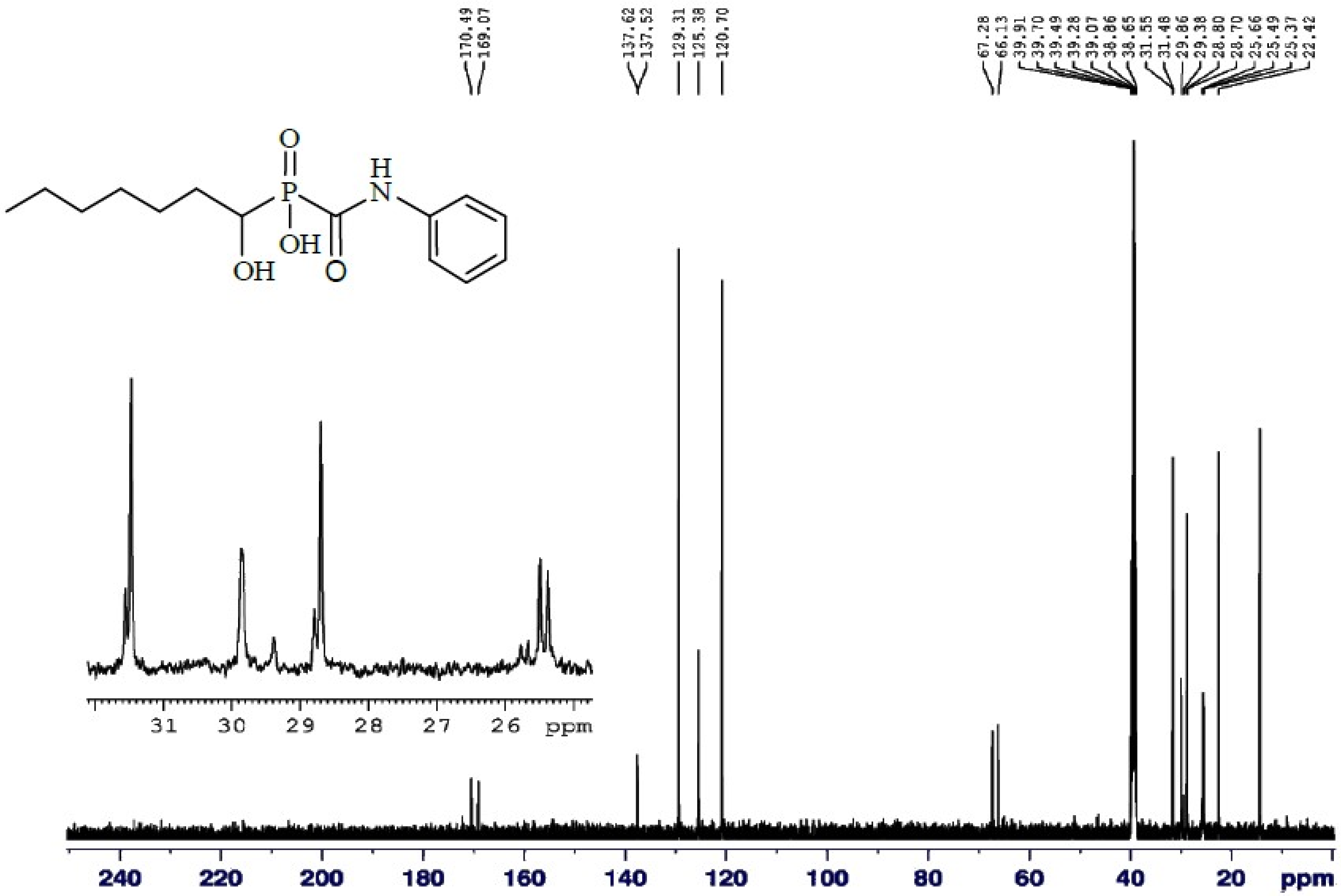

Figure S29: ^{13}C NMR Spectra of 3j

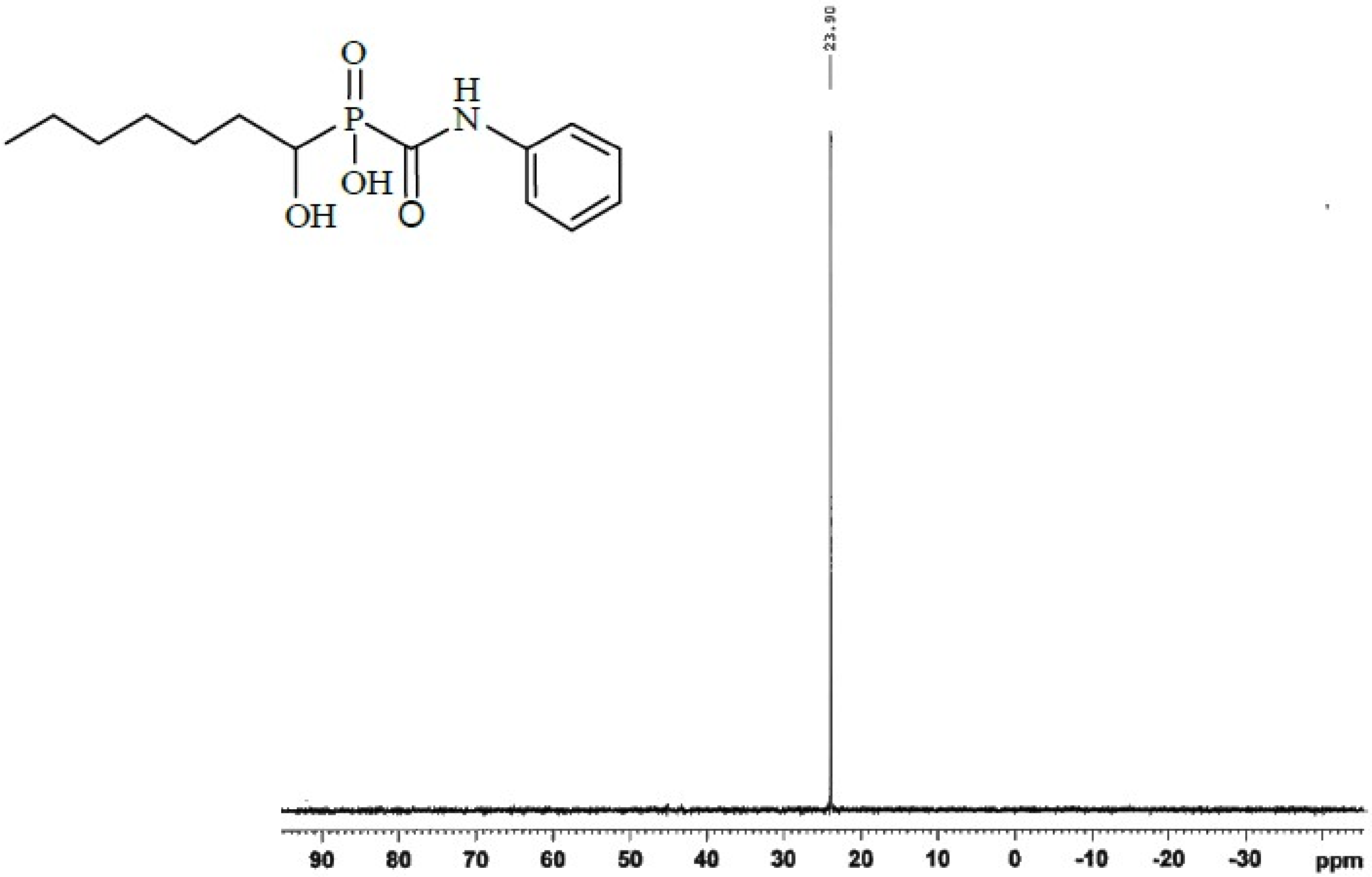

Figure S30: ^{31}P NMR Spectra of 3j

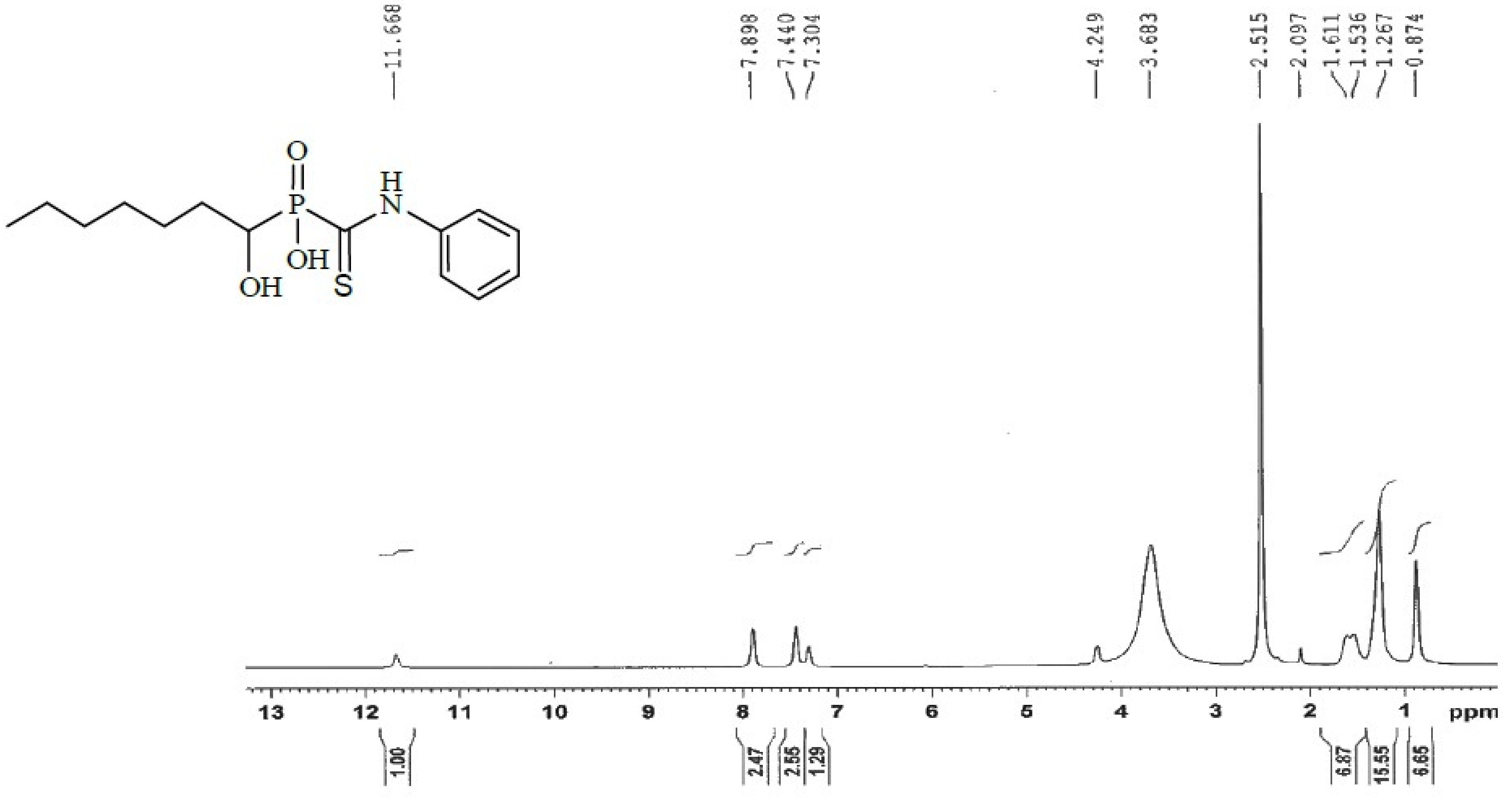

Figure S31: ^{1}H NMR Spectra of 3k

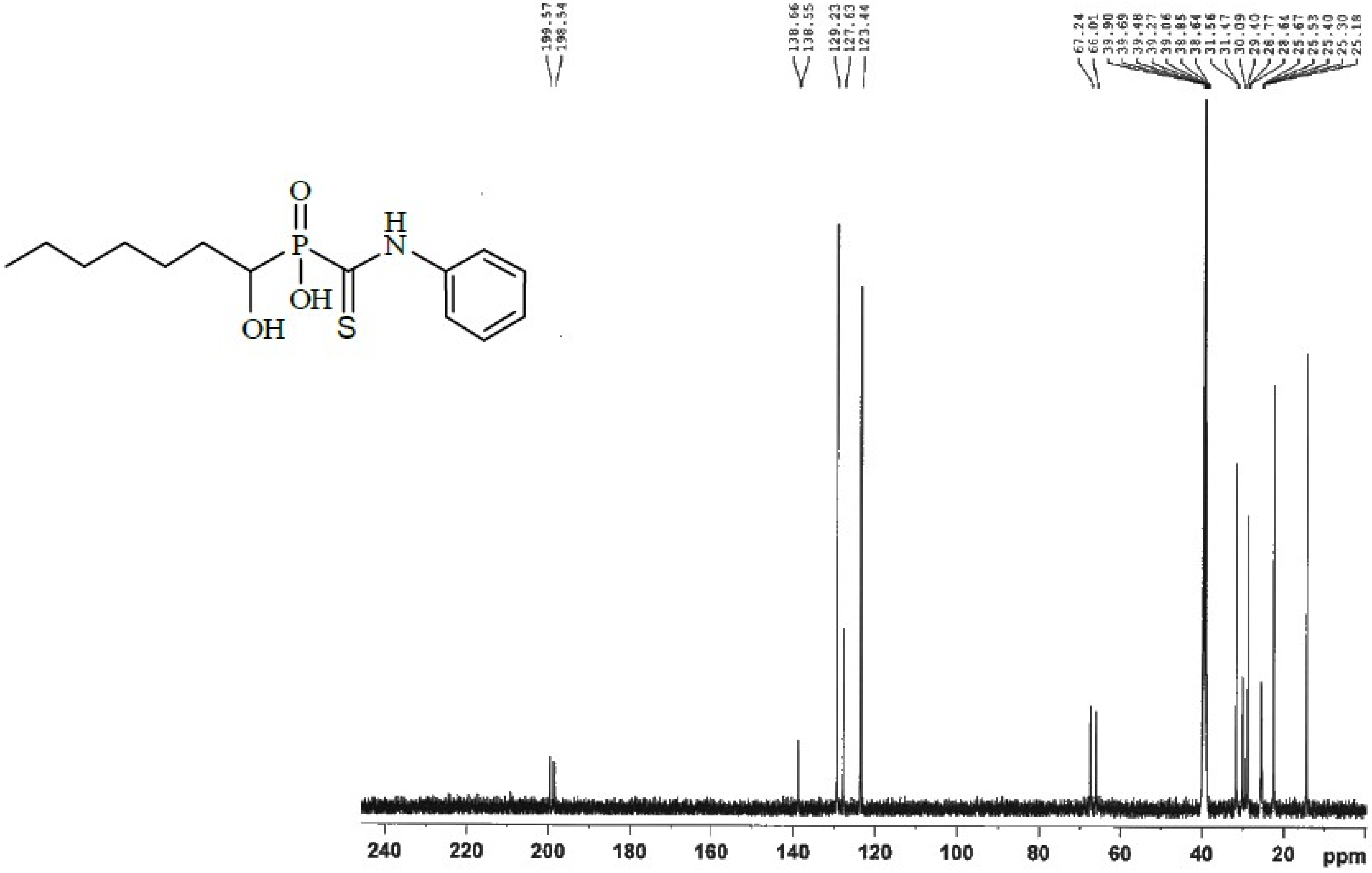

Figure S32: ^{13}C NMR Spectra of 3k

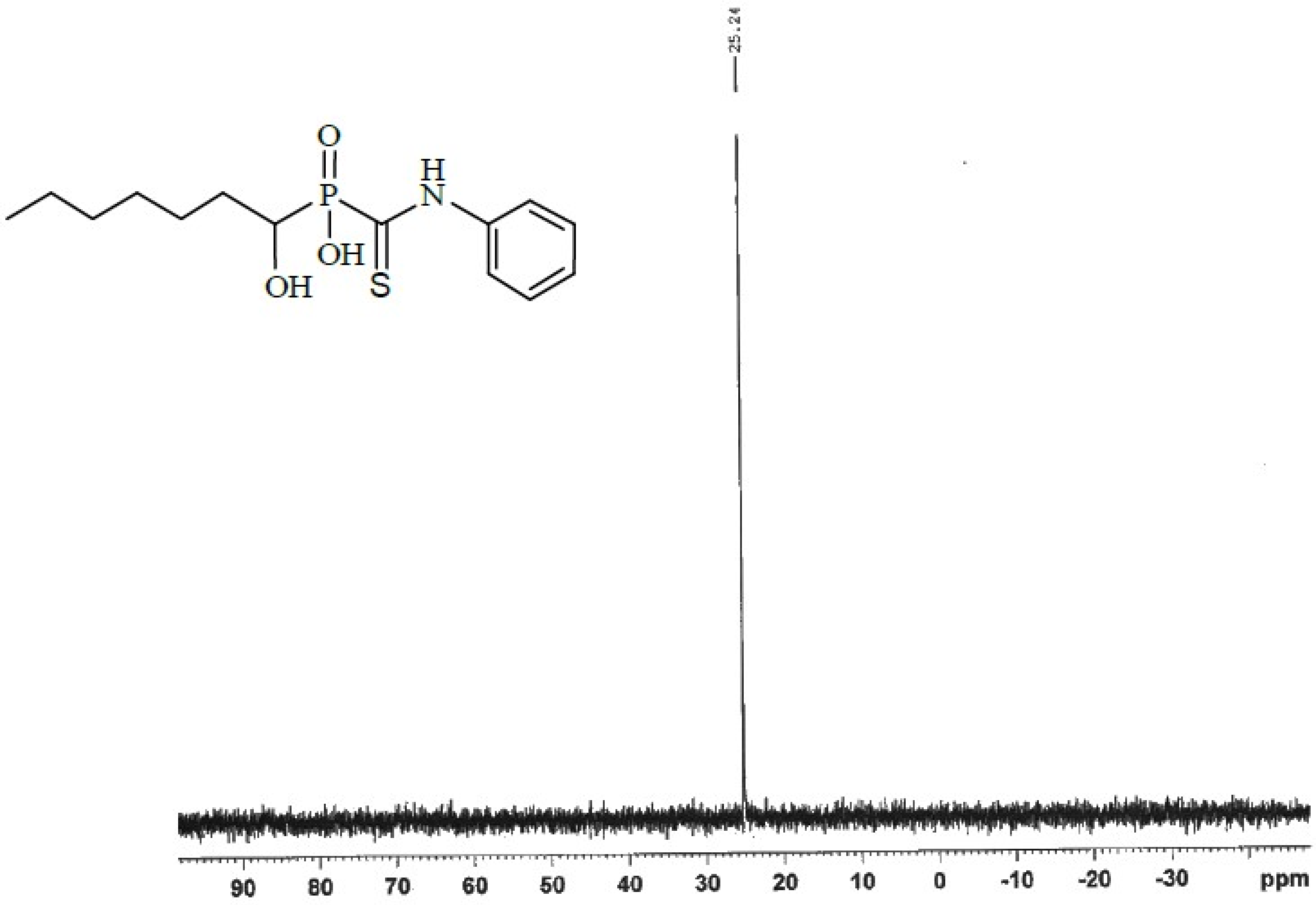

Figure S33: ^{31}P NMR Spectra of 3k

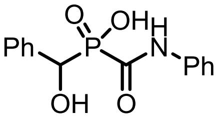

3a

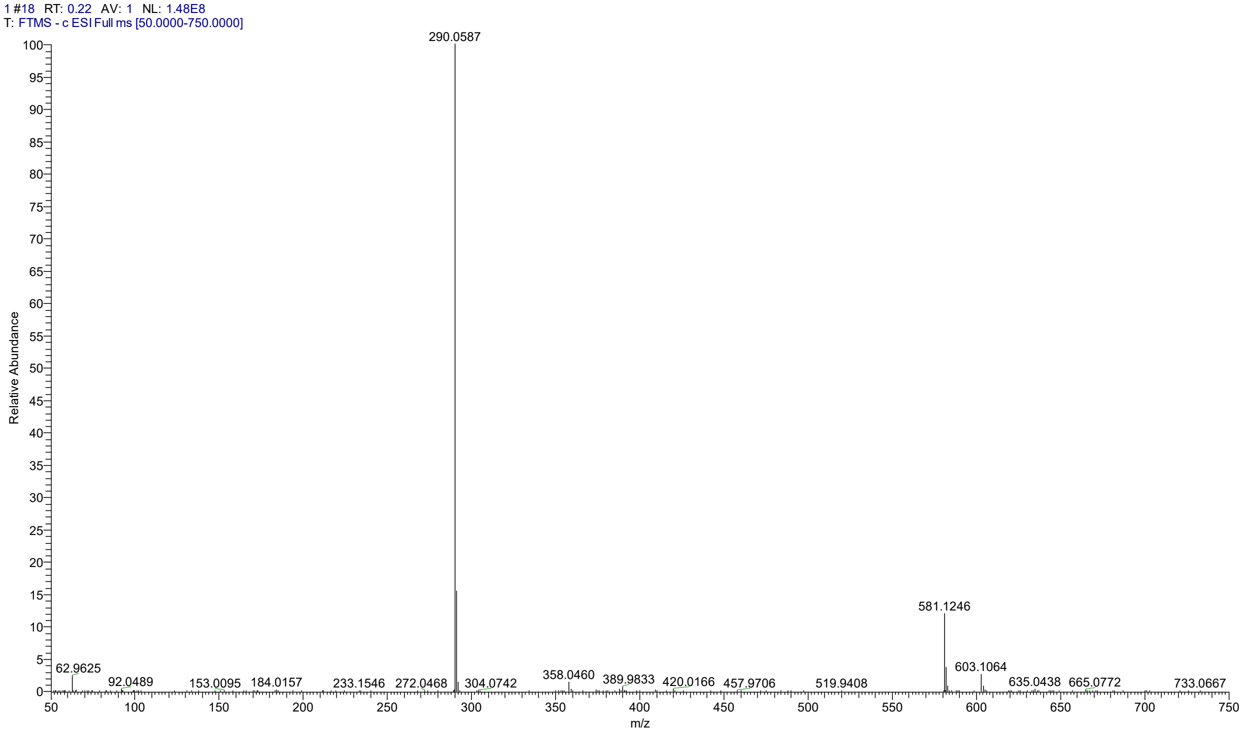

Figure S34: LCMS of 3a

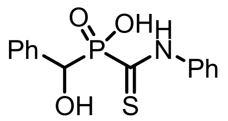

3b

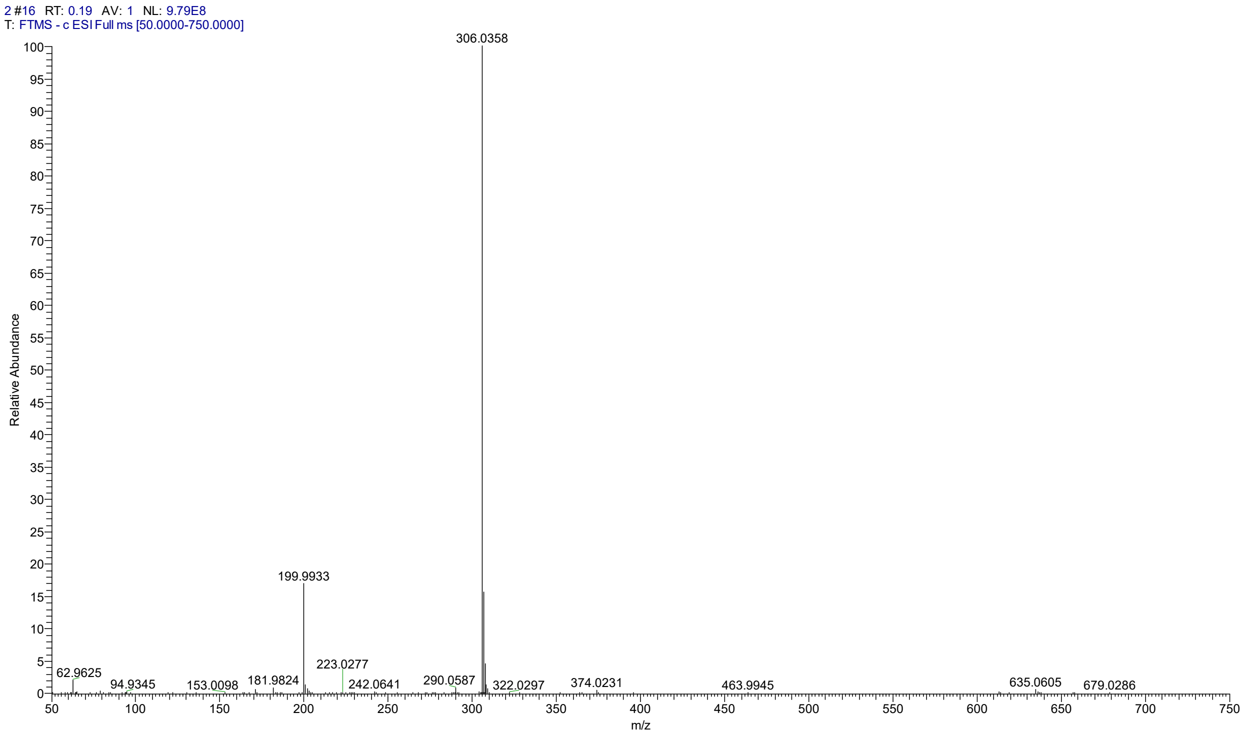

Figure S35: LCMS of 3b

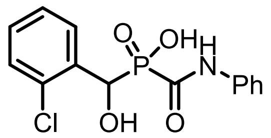

3c

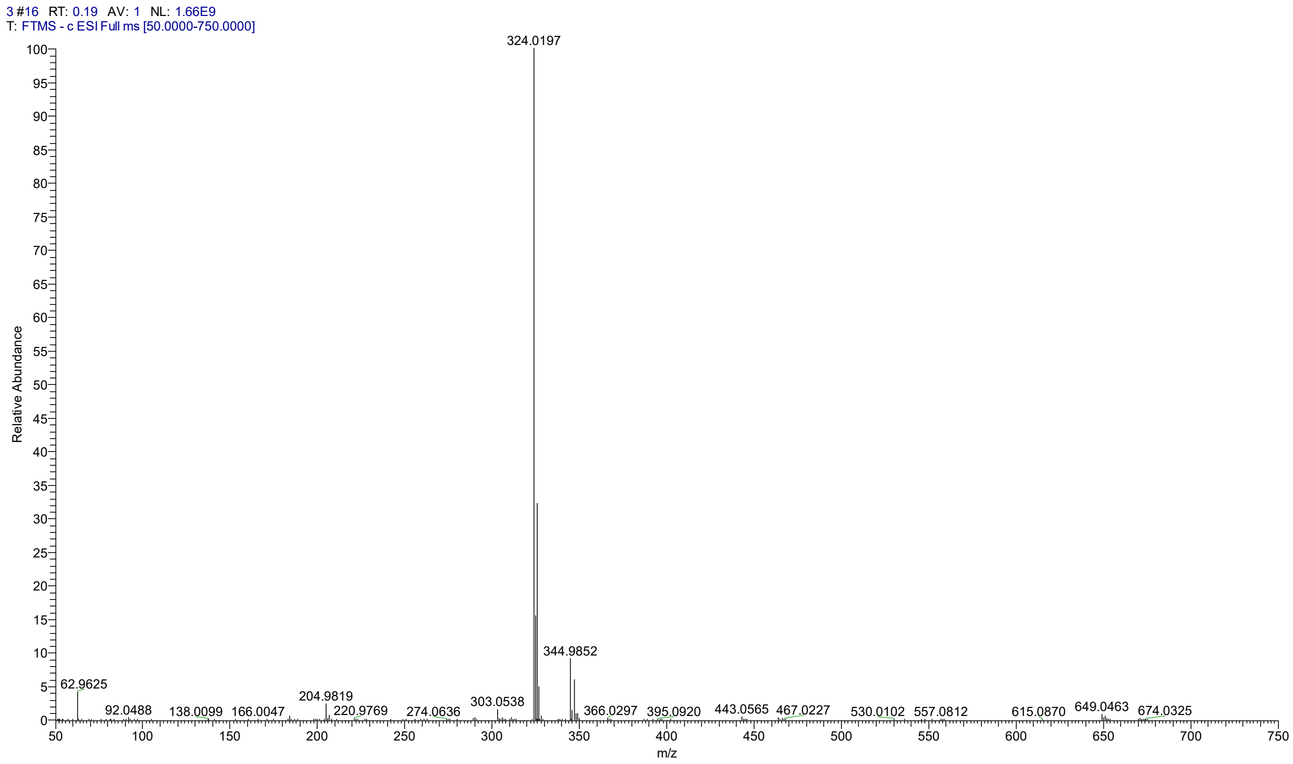

Figure S36: LCMS of 3c

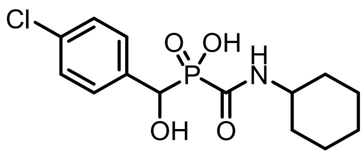

3d

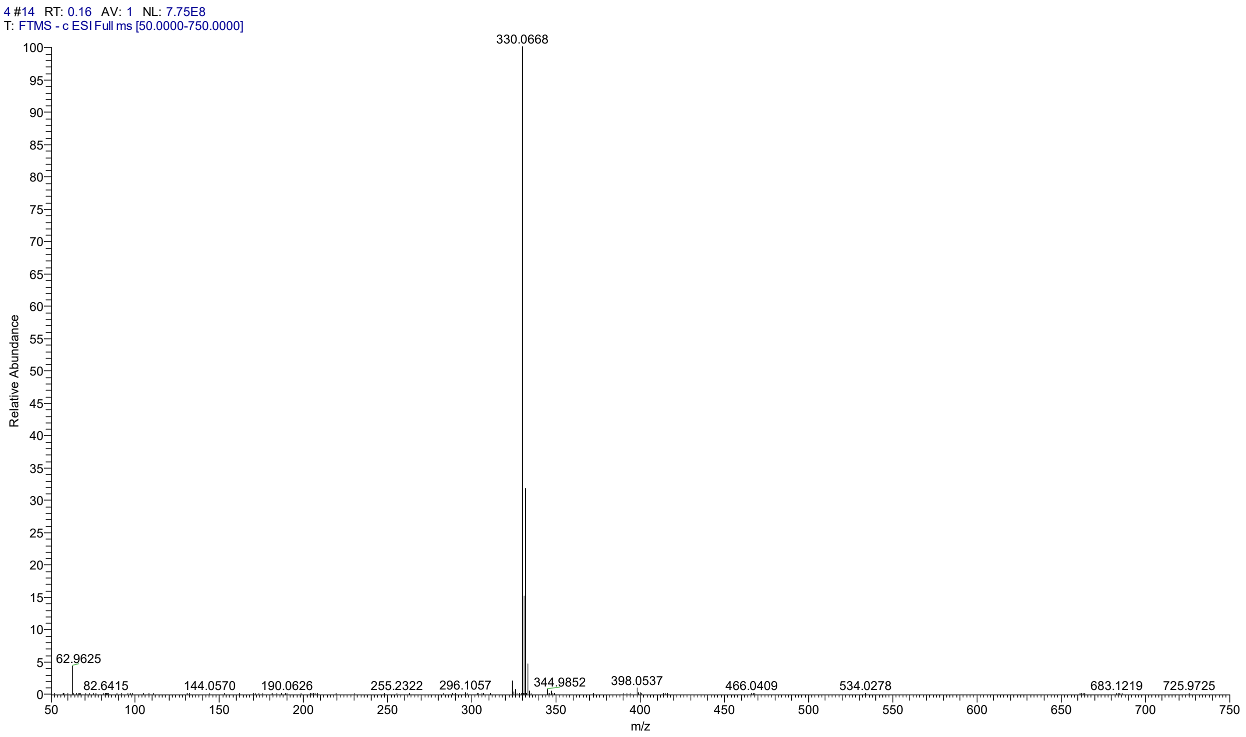

Figure S37: LCMS of 3d

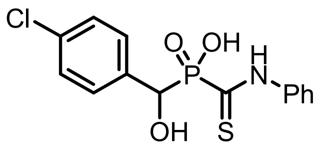

3e

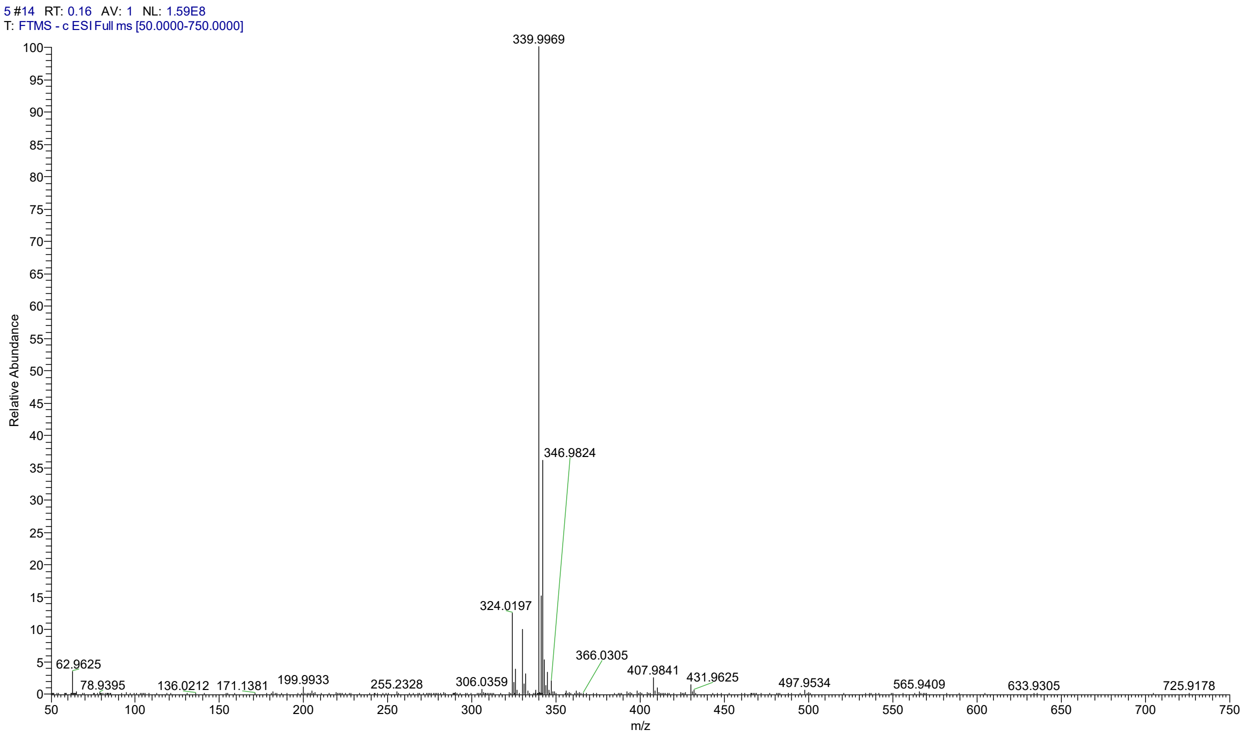

Figure S38: LCMS of 3e

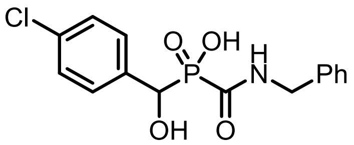

3f

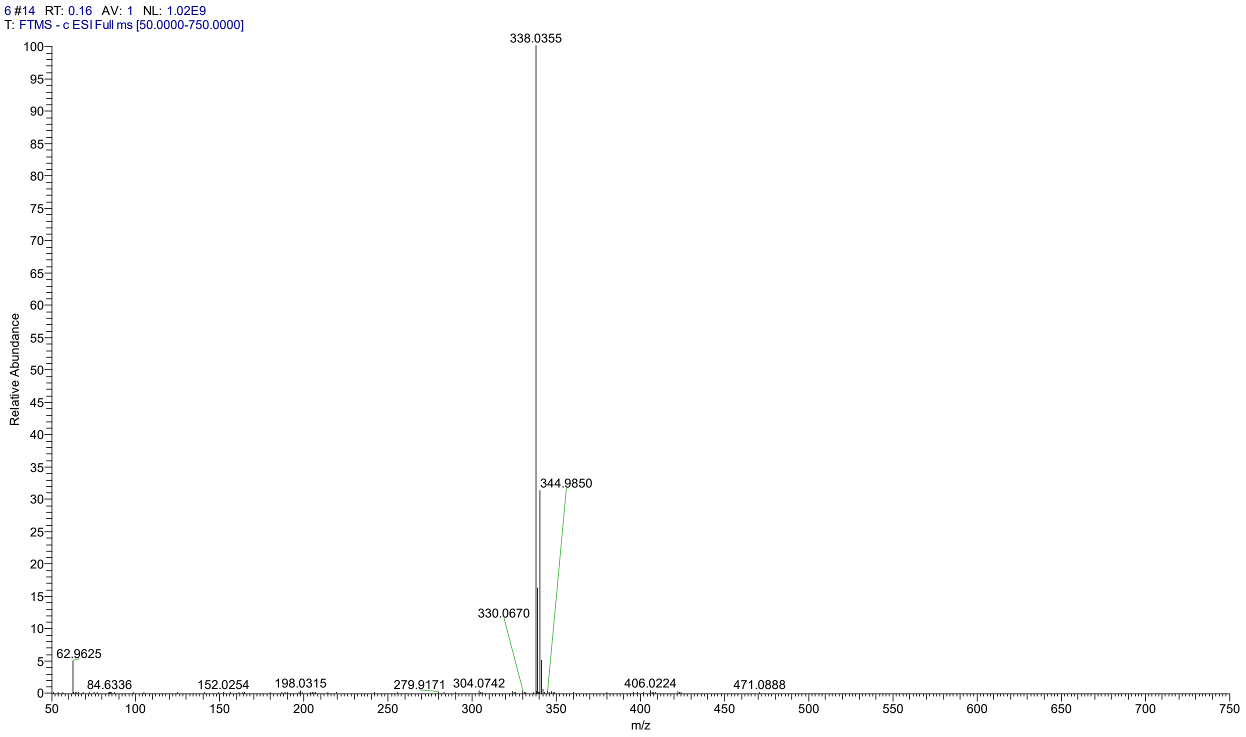

Figure S39: LCMS of 3f

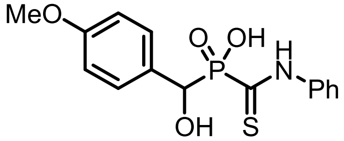

3g

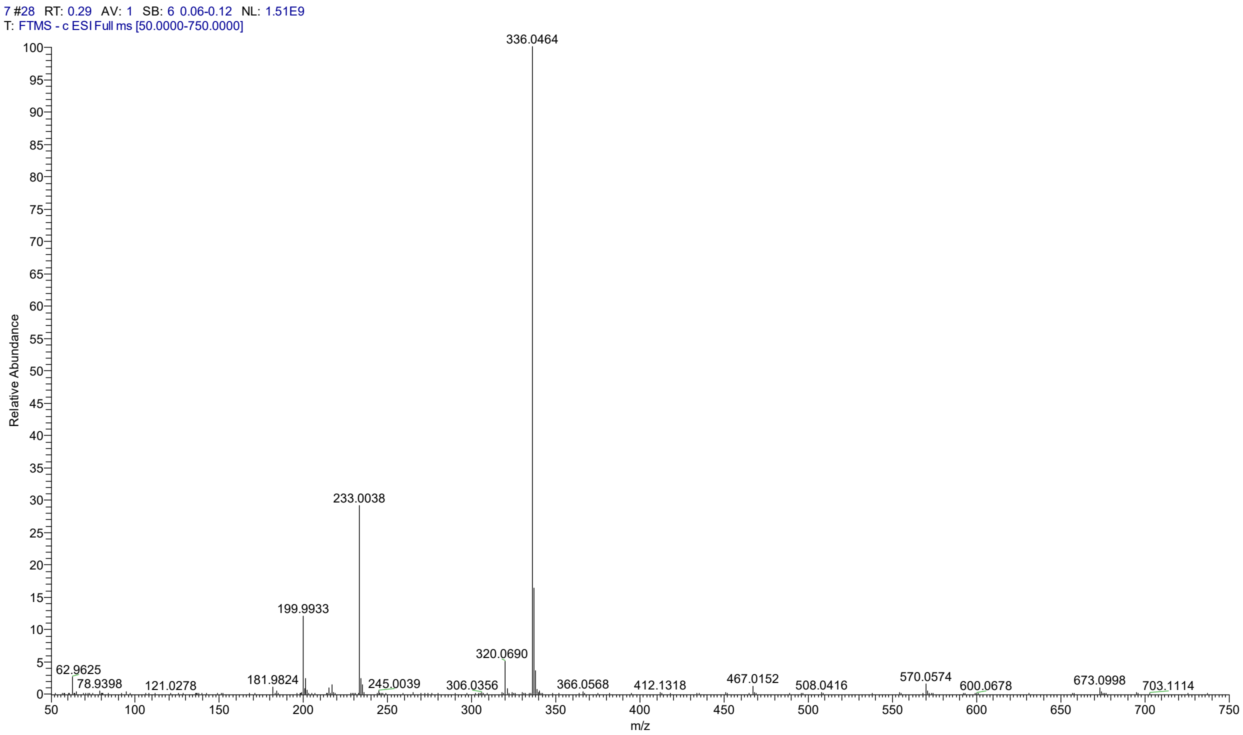

Figure S40: LCMS of 3g

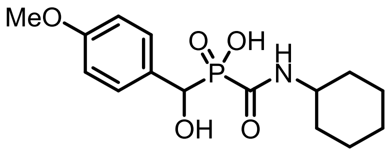

3h

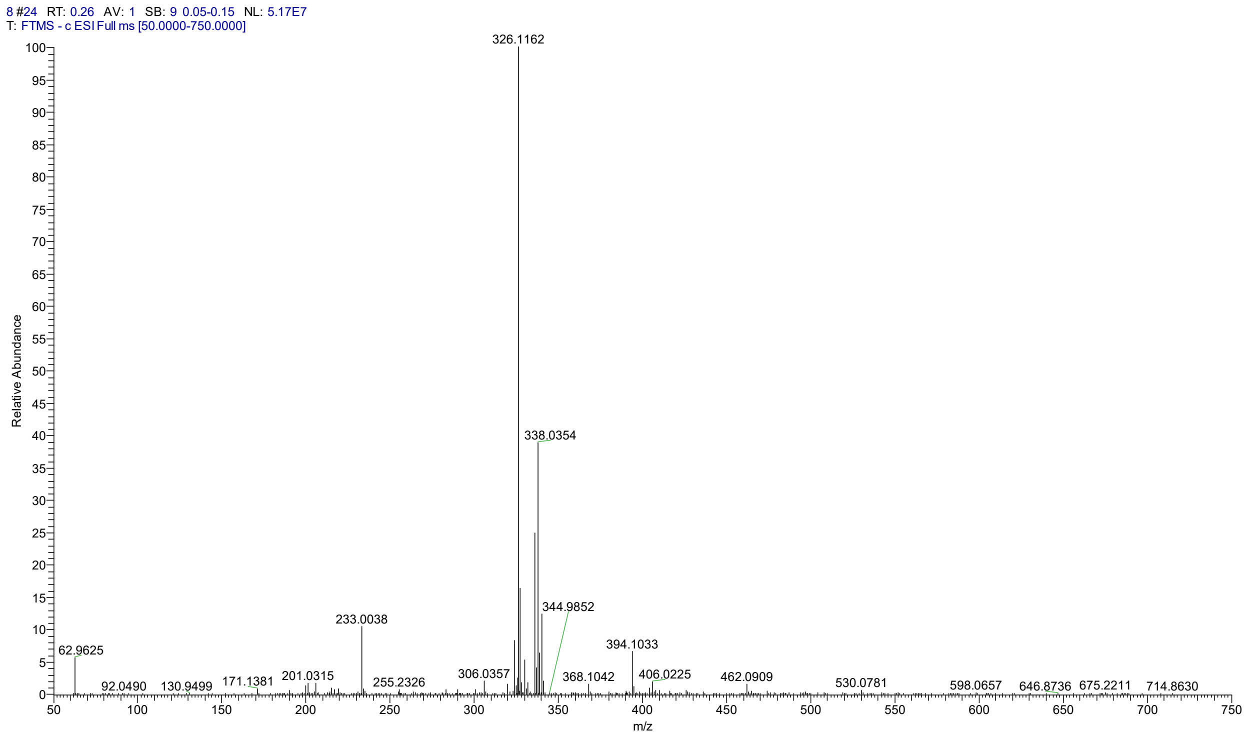

Figure S41: LCMS of 3h

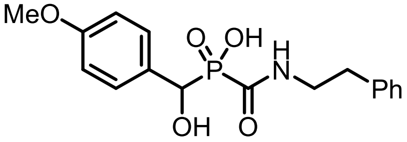

3i

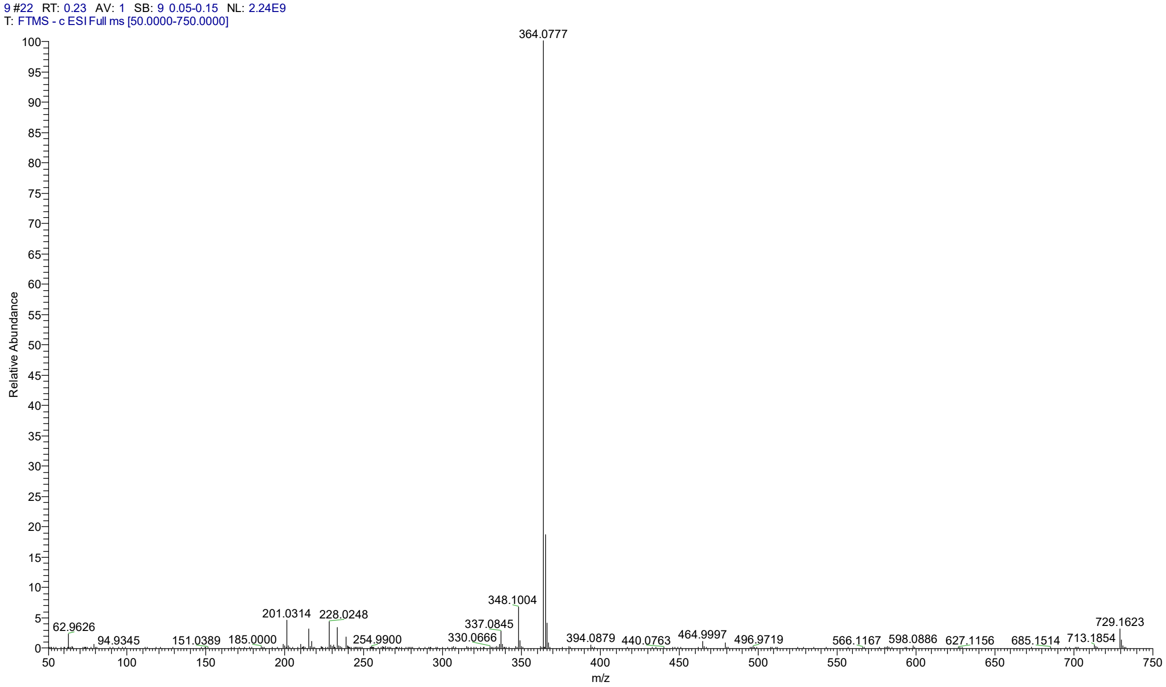

Figure S42: LCMS of 3i

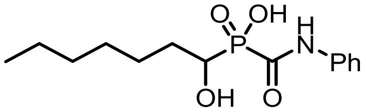

3j

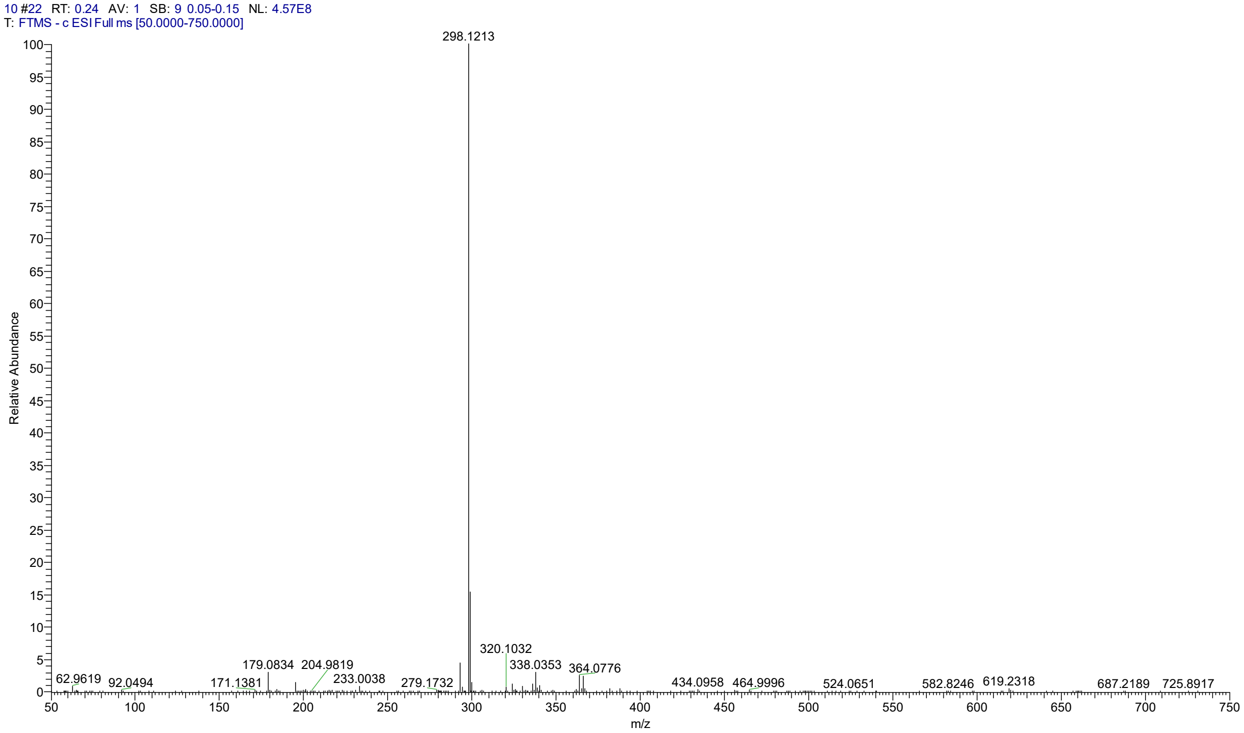

Figure S43: LCMS of 3j

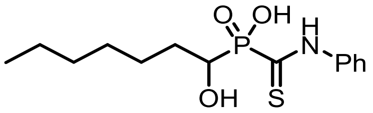

3k

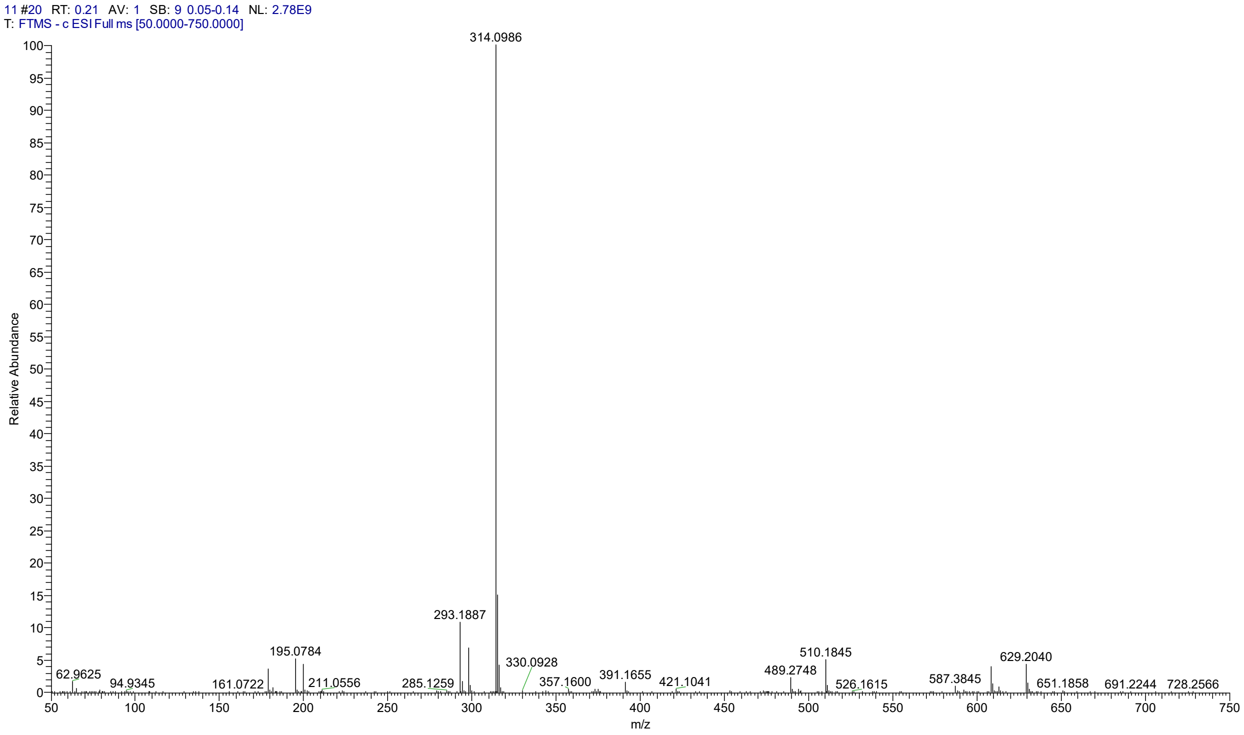

Figure S44: LCMS of 3k

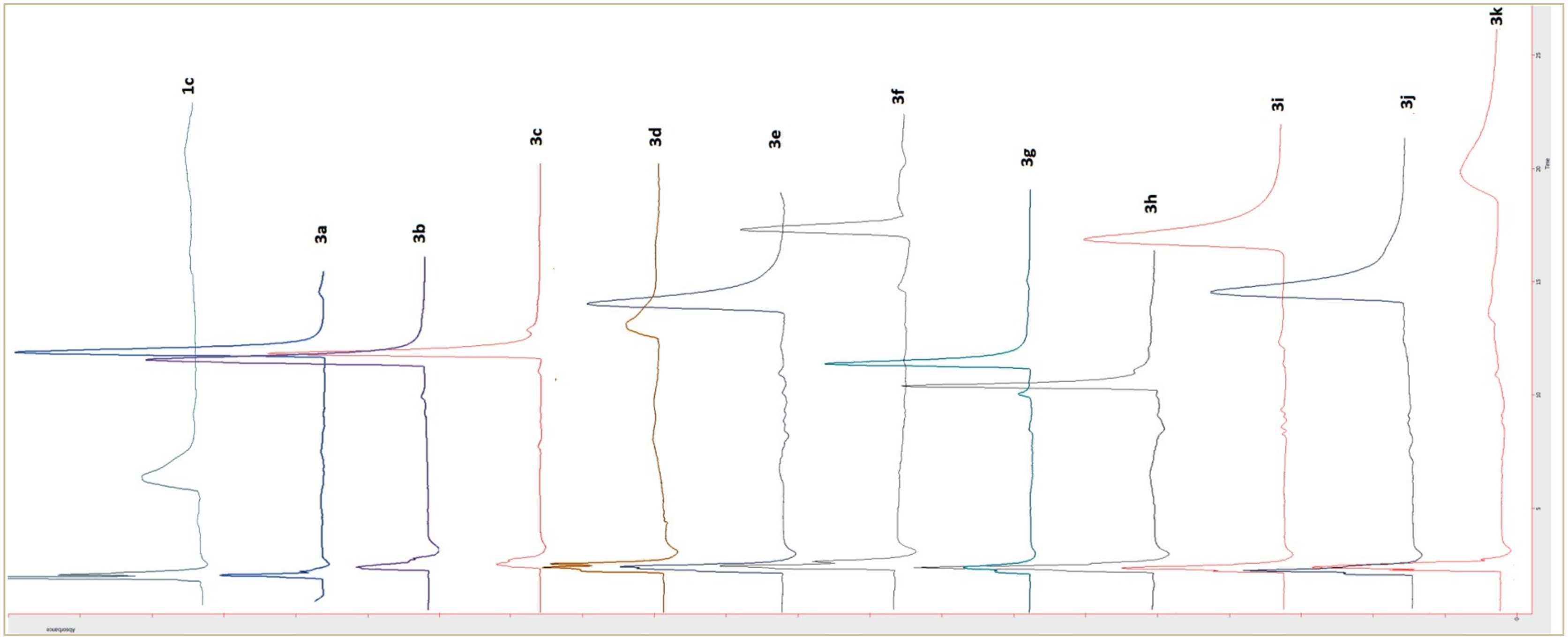

Figure S45: HPLC-UV chromatograms of 50 mg L^{-1} solutions of the compounds
